# Pseudomonas aeruginosa Regulatory Protein AnvM Controls Pathogenicity in Anaerobic Environments and Impacts Host Defense

**DOI:** 10.1128/mBio.01362-19

**Published:** 2019-07-23

**Authors:** Yingchao Zhang, Chuan-min Zhou, Qinqin Pu, Qun Wu, Shirui Tan, Xiaolong Shao, Weitong Zhang, Yingpeng Xie, Rongpeng Li, Xue-jie Yu, Rui Wang, Liang Zhang, Min Wu, Xin Deng

**Affiliations:** a Department of Biomedical Sciences, City University of Hong Kong, Hong Kong, People’s Republic of China; b Department of Biomedical Sciences, University of North Dakota, Grand Forks, North Dakota, USA; c State Key Laboratory of Virology, School of Health Sciences, Wuhan University, Wuhan, People’s Republic of China; d Key Laboratory of Biotechnology for Medicinal Plants of Jiangsu Province, Jiangsu Normal University, Xuzhou, Jiangsu, People’s Republic of China; College of Veterinary Medicine, Cornell University

**Keywords:** AnvM, *Pseudomonas aeruginosa*, anaerobic

## Abstract

Pseudomonas aeruginosa, one of the most common pathogens in hospital-acquired infections, is tightly controlled by a multilayered regulatory network, including the quorum sensing system (QS), the type VI secretion system (T6SS), and resistance to host immunity. We found that the P. aeruginosa 3880 (PA3880) gene, which encodes an unknown protein, acts as a regulator of anaerobic metabolism in response to oxidative stress and virulence in P. aeruginosa. More than 30 PA3880 homologs were found in other bacterial genomes, indicating that PA3880 is widely distributed in the *Bacteria* kingdom as a highly conserved gene. Deletion of the PA3880 gene changed the expression levels of more than 700 genes, including a group of virulence genes, under both aerobic and anaerobic conditions. To further study the mechanisms of PA3880-mediated regulation in virulence, we utilized a bacterial two-hybrid assay and found that the PA3880 protein interacted directly with QS regulator MvfR and anaerobic regulator Anr. Loss of the PA3880 protein significantly blunted the pathogenicity of P. aeruginosa, resulting in increased host survival, decreased bacterial burdens, reduced inflammatory responses, and fewer lung injuries in challenged mice hosts. Mechanistically, we found that Cys44 was a critical site for the full function of PA3880 in influencing alveolar macrophage phagocytosis and bacterial clearance. We also found that AnvM directly interacted with host receptors Toll-like receptor 2 (TLR2) and TLR5, which might lead to activation of the host immune response. Hence, we gave the name AnvM (anaerobic and virulence modulator) to the PA3880 protein. This characterization of AnvM could help to uncover new targets and strategies to treat P. aeruginosa infections.

## Abstract (precis)

Infections by Pseudomonas aeruginosa, one of the most frequently isolated human pathogens, can create huge financial burdens. However, knowledge of the molecular mechanisms involved in the pathogenesis of P. aeruginosa remains elusive. We identified AnvM as a novel regulator of virulence in P. aeruginosa. Deletion of *anvM* altered the expression levels of more than 700 genes under aerobic and anaerobic conditions, including quorum sensing system genes and oxidative stress resistance genes. AnvM directly interacted with MvfR and Anr, thus regulating their downstream genes. More importantly, AnvM directly bound to TLR2 and TLR5, which turn on the host immune response. These findings provide insights into the significance of AnvM homologs in pathogenic bacteria and suggest a potential drug target against bacterial infection.

## INTRODUCTION

Pseudomonas aeruginosa is an opportunistic pathogen that causes serious infections in the immunocompromised population (1). Worldwide, it is estimated that more than 10% of hospital infections are caused by P. aeruginosa (2). An important feature of this pathogen is that it has high intrinsic and acquired resistance to many antibiotics, which makes it both difficult to treat and a model pathogen for evaluating bacterial pathogenicity and drug resistance (3, 4).

The pathogenicity of P. aeruginosa mainly depends on a group of virulence determinants, including alginate, rhamnolipids, and a group of cytotoxic proteins (5–9). The expression of these factors is precisely regulated by a complex regulatory network, including quorum sensing (QS) systems, type III secretion systems (T3SS), and type VI secretion systems (T6SS) (10, 11). Although more than 20 transcription factors (such as LasR, RhlR, PqsR, RsaL, VqsM, GacA, RpoN, CdpR, VqsR, and ExsA) or enzymes have been characterized as playing important roles in regulating this network, the molecular regulatory mechanisms remain largely unknown (10, 11).

P. aeruginosa needs to overcome the high concentration of reactive oxygen species (ROS) before successfully infecting host cells. In recent years, studies have found several transcription factors and enzymes that are directly oxidized by ROS during oxidative stress, including OxyR, OhrR, Anr, MgrA, SarZ, MexR, OspR, LasR, ExsC, ArcA, and GAPDH (glyceraldehyde-3-phosphate dehydrogenase) (12–16). In the presence of ROS, the thiol groups of specific cysteines on these proteins are oxidized to either sulfonic acid or disulfide bonds, altering the conformation of the proteins and leading to significant functional changes. Ultimately, ROS changes the expression levels of downstream genes that are controlled by these virulence proteins, leading to reduced pathogenicity and increased resistance to antibiotics. These ROS-sensing proteins can be used as targets for the development of a new generation of antimicrobial agents (12).

To comprehensively identify all ROS-sensing cysteines in P. aeruginosa, we used a powerful chemical biological approach: isotopic tandem orthogonal proteolysis–activity-based protein profiling (isoTOP-ABPP) (15, 17). In previous work, we found 80 cysteines that are highly sensitive to oxidative stress. The most sensitive cysteine is Cys44 (sensitivity ratio = 0.09) in the functional unknown protein P. aeruginosa 3880 (PA3880). In the current study, we attempted to fully characterize this protein and found that it plays important roles in response to oxidative stress and virulence and to host response. Therefore, we named PA3880 AnvM, for anaerobic and virulence modulator. Our report demonstrates that AnvM is a novel virulence regulator and provides insights into AnvM-mediated pathogenesis and host interactions in a mouse model.

## RESULTS

### AnvM-like proteins are distributed in a wide range of bacterial species.

**FIG 1:**
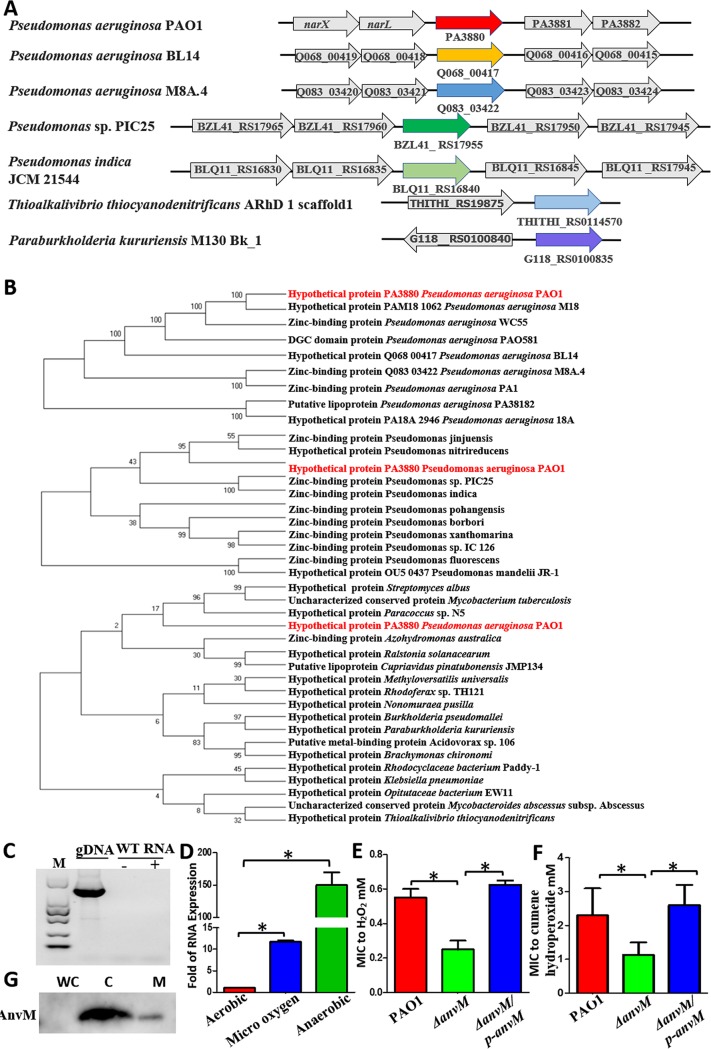
Prevalence of AnvM-like protein. (A) The location of *anvM* in bacteria. (B) Phylogenetic tree of AnvM. Nine strains of P. aeruginosa, 11 strains of *Pseudomonas* spp., and 19 genera were included in the phylogenetic tree. The phylogenetic relations were inferred using the neighbor-joining method, and evolutionary distances were computed using the Poisson correction method. Analyses were performed with MEGA7 software. (C) RT-PCR showed that *anvM* was not in the same operon as *narXL*. gDNA, genomic DNA; M, molecular mass marker. (D) Real-time PCR showed that *anvM* is highly activated under microaerobic and anaerobic conditions. Anaerobic culturing was performed in 100-ml hermetically sealed bottles containing an AnaeroPack-Anaero (MGC), and micro-oxygen culturing was performed in 100-ml hermetically sealed bottles containing an AnaeroPack-MicroAero (MGC). (E and F) The *anvM* deletion strain was more sensitive to H_2_O_2_ (E) and cumene hydroperoxide (CHP) (F) than the WT strain. *, *P* < 0.05; **, *P* < 0.01; ***, *P* < 0.001 (one-way ANOVA with Tukey *post hoc* test). Serial 2-fold dilutions were prepared in LB that ranged from 10 mM to 0.005 mM H_2_O_2_ and 30 mM to 0.015 mM CHP. A 0.2-ml volume of each dilution was distributed over a 96-well polystyrene microwell plate. A 0.5 McFarland standard suspension (∼1.5 × 10^8^ CFU/ml) was prepared from a fresh overnight culture of PAO1, strain Δ*anvM*, and strain Δ*anvM*/p-*anvM*, and 20 μl was added to each microwell in the 96-well plate in triplicate. Growth was monitored every 4 h for 24 h at 37°C using a Multiskan GO microplate spectrophotometer (Thermo Fisher Scientific, Vantaa, Finland). (G) Subcellular localization of AnvM. Western blot analysis showed that AnvM was located mostly on the cytoplasm (C) fraction and that a few AnvM proteins were located on the membrane (M) fraction of P. aeruginosa. The whole-cell lysates (WC) were used as controls.

The PA3880 gene encodes an unknown protein, and its biological function has not been reported. On the P. aeruginosa genome, the PA3880 gene is located downstream of the two-component system *narXL* and is followed by two other incompletely characterized genes, PA3881 and PA3882 (Fig. 1A). The distances between PA3880 and its flanking genes are 84 bp upstream and 171 bp downstream, so it is annotated as a single operon, which we named AnvM.

To explore whether AnvM was widely distributed among bacteria, we searched for its homologs in the National Center for Biotechnology Information (NCBI) database and performed a phylogenetic analysis. The majority of AnvM homologs in other bacterial species were located in independent operons (Fig. 1A) and were found in other strains of P. aeruginosa, other *Pseudomonas* species, and other genera (Fig. 1A; see also Fig. S1A to C in the supplemental material). The AnvM homologs were distributed in a wide spectrum of species in the kingdom that includes bacteria (Fig. 1B), suggesting that they may have originated from a common ancestor and may have similar functions.

10.1128/mBio.01362-19.1 FIG S1 Protein sequence alignments. (A) Multiple-sequence alignment of AnvM homologs in genomes of P. aeruginosa. (B) Multiple-sequence alignment of AnvM homologs in *Pseudomonas* species. (C) Multiple-sequence alignment of AnvM homologs in other genus. Download FIG S1, TIF file, 2.6 MB. Copyright © 2019 Zhang et al. 2019 Zhang et al. This content is distributed under the terms of the Creative Commons Attribution 4.0 International license.

Although *anvM* is not annotated in the same operon as *narXL*, it is closer to *narX* and *narL* (*narX*/*L*) (Fig. 1A), which led us to test whether *anvM* shares an operon with *narXL*. We performed a real-time PCR (RT-PCR) assay using total RNA from the wild-type (WT) strain, an upstream primer of *narX*, and a downstream primer of *anvM*. The result was negative, indicating that *anvM* was not in the same operon as *narXL* (Fig. 1C). We also tested whether nitrate, which modulates the expression of *narX*/*L*, can affect the expression of *anvM*. We found that *anvM* was positively regulated by nitrate (Fig. S2A). The *narX* promoter activity seen in the WT strain did not differ from that in a Δ*anvM* strain (Fig. S2B). We also tested for the interaction of AnvM and NarX/L using a bacterial two-hybrid assay; there was no clear interaction between them (Fig. S2C). We thus concluded that *anvM* was not genetically related to NarX/L.

10.1128/mBio.01362-19.2 FIG S2 AnvM did not interact with NarL, NarX, NarXL, LasR, and LasB. (A) Conditions leading to modulating expression of *narX*/*L* can also affect the expression of *anvM*. (B) Expression of *narXL*-lux in wild-type PAO1 and Δ*anvM* strains. (C) Bacterial two-hybrid assay revealed that AnvM did not interact with NarL, NarX, and NarXL. (D) Results of bacterial two-hybrid assay revealed that AnvM did not interact with LasR. (E) Bacterial two-hybrid assay revealed that AnvM did not interact with LasB. The recombinant strain harboring different proteins was separately streaked on nonselective and dually selective media (3-amino-1,2,4-triazole plus streptomycin). The strain expressing LGF2 and GAII 1P was used as a positive control. The experiments were repeated at least three times, and similar results were observed. Download FIG S2, TIF file, 2.7 MB. Copyright © 2019 Zhang et al. 2019 Zhang et al. This content is distributed under the terms of the Creative Commons Attribution 4.0 International license.

### The characteristics and subcellular localization of AnvM.

Given that the 44th cysteine of AnvM is the most sensitive cysteine in the P. aeruginosa proteome (15), we set out to characterize the function of AnvM and its Cys44. The AnvM protein is composed of 131 amino acids, including 11 cysteines and a DGC conservative sequence, which is predicted to be a binding site of zinc (Fig. S3). Cys44 was the only Cys site identified as a putative oxidative-element-sensing amino acid in our previous proteomic approach. To study its function, we searched for PA3880 (*anvM*) in the NCBI Gene Expression Omnibus (GEO) database. A previously identified gene microarray data set (GEO accession no. GSE6741) showed that the transcription level of *anvM* under anaerobic conditions was 100 times higher than that under normal aerobic conditions (18). Consistent with the microarray assay, our real-time quantitative PCR (RT-qPCR) analysis demonstrated the same results (Fig. 1C). We also confirmed the results of a previous study showing that microaerobiosis can affect the expression of *anvM* (18) (Fig. 1D). These results indicate that AnvM has an important function under anaerobic conditions.

10.1128/mBio.01362-19.3 FIG S3 Transcriptome analysis of strains PAO1 and Δ*anvM* and verification of *anvM* mutant and RNA-seq data. Distribution of differentially expressed genes (strain Δ*anvM* versus PAO1) within functional GO (Gene Ontology) categories. (A) Numbers of upregulated and downregulated DEGs under aerobic conditions. (B and C) Genes were upregulated (B) and downregulated (C) in strain Δ*anvM* under aerobic conditions. (D) Numbers of upregulated and downregulated DEGs under anaerobic conditions. (E and F) Genes were upregulated (E) and downregulated (F) in strain Δ*anvM* under anaerobic conditions. (G) Sequencing the PCR products of *anvM* mutant. (H) RT-qPCR was performed to measure the transcription level of *anvM*. Sequencing and RT-qPCR results validated the expectation that the mutant strain had been constructed. (I) According to RNA-seq assay, we observed abolished reads of the *anvM* gene in strain Δ*anvM* compared to the wild type (WT). (J) RT-qPCR validated the RNA-seq data. Download FIG S3, TIF file, 1.3 MB. Copyright © 2019 Zhang et al. 2019 Zhang et al. This content is distributed under the terms of the Creative Commons Attribution 4.0 International license.

As AnvM carries some of the most sensitive cysteines in P. aeruginosa (15), we tested whether this protein is involved in oxidative sensing and response. We constructed a deletion of *anvM* by gene exchange, resulting in an *anvM* deletion mutant. We measured the MIC of hydrogen peroxide (H_2_O_2_) and cumene hydroperoxide (CHP) in the WT strain, strain Δ*anvM*, and the corresponding complemented strain carrying the plasmid pAK1900-*anvM*. The Δ*anvM* strain was 2-fold more sensitive to H_2_O_2_ and CHP stress than the WT strain (Fig. 1E and F), suggesting that AnvM plays an important role in ROS defense.

To confirm the localization of AnvM in P. aeruginosa PAO1, we fused the full-length *anvM* gene in-frame with a FLAG tag, and this recombinant protein was expressed in PAO1. Cytoplasmic and membrane fractions of P. aeruginosa expressing AnvM-FLAG were isolated as described previously (19). The resulting fractions were separated by sodium dodecyl sulfate-polyacrylamide gel electrophoresis (SDS-PAGE), and the expression levels of the fusion protein were evaluated by Western blotting using anti-FLAG antibodies (Abs). The recombinant AnvM-FLAG fusion protein was present in both the cytoplasmic fraction (C) and the membrane fraction (M) (Fig. 1G).

### Transcriptomic analyses revealed the presence of AnvM regulons under aerobic and anaerobic conditions.

To further characterize the biological function of AnvM, we carried out global transcriptomic profiling of the wild-type (WT) P. aeruginosa strain and the Δ*anvM* strain. A transcriptome sequencing (RNA-seq) analysis was performed to profile the differentially expressed genes (DEG) in the Δ*anvM* and WT strains. Under aerobic conditions, the expression levels of 350 genes were upregulated while those of 340 genes were downregulated by at least 2-fold in the Δ*anvM* strain compared to the WT strain (Fig. S3A; see also Table S1C in the supplemental material). Under anaerobic conditions, 326 genes were upregulated and 421 genes were downregulated in strain Δ*anvM* (more than 2-fold expression) (Fig. S3D; see also Table S1D). A gene ontology (GO) enrichment analysis revealed genes involved in metabolism, oxidoreductase activity, transmembrane component, and transcription regulation under aerobic conditions (*P* < 0.05; Fig. S3B and C). The enriched genes were involved in metabolism, transcription regulation, transmembrane component, motility, response to oxidative stress, and iron ion binding (*P* < 0.05; Fig. S3E and F). AnvM was observed to play a critical role in regulating multiple biological pathways, including those for oxidoreductase activity, transmembrane components, transcription regulation, motility, iron binding, response to oxidative stress in the QS system, and metabolism. Many genes in the *anvM* regulon (DEG) were related to pathogenicity, such as those involved in alginate biosynthesis (*algE*, *algF*, *algJ*, and *algX*), the type III secretion system (*exsB*, *pscU*, *pscR*, and *pcrV*), motility (*fliF*), and the quorum sensing system (*pqsA*). Given that the T3SS of P. aeruginosa is stimulated under hypoxic conditions, *anvM* might play an important role in the activation and function of the type III secretion system (T3SS) (20). In sum, our transcriptomic profiling analysis characterized the *anvM* regulon in P. aeruginosa and indicated that AnvM functioned as a key regulator controlling multiple cellular functions.

10.1128/mBio.01362-19.9 TABLE S1 Bacterial strains, plasmids, and primers and RNA-seq data. (A) Bacterial strains and plasmids used in this study. (B) Primers used in this study. (C) Differential expression of 693 genes in Pseudomonas aeruginosa PAO1 under aerobic conditions. (D) Differential expression of 749 genes in Pseudomonas aeruginosa PAO1 under anaerobic conditions. Download Table S1, DOCX file, 0.1 MB. Copyright © 2019 Zhang et al. 2019 Zhang et al. This content is distributed under the terms of the Creative Commons Attribution 4.0 International license.

We verified the Δ*anvM* strain by sequencing the PCR products of strain Δ*anvM* using primers located upstream and downstream of *anvM* (Fig. S3G). The mutant strain was constructed. We tested the expression of *anvM* in both the WT and Δ*anvM* strains by RT-qPCR (Fig. S3H), and the results confirmed that expression of *anvM* was almost abolished in the mutant strain. This was consistent with the RNA-seq result, which showed abolished reads of the *anvM* in the Δ*anvM* strains compared to the WT strain (Fig. S3I). We also verified the 10 downstream genes of *anvM* using RT-qPCR (Fig. S3J), and the results indicated that the RNA-seq data were reliable.

### AnvM interacted with QS regulator MvfR to regulate bacterial pathogenicity.

**FIG 2:**
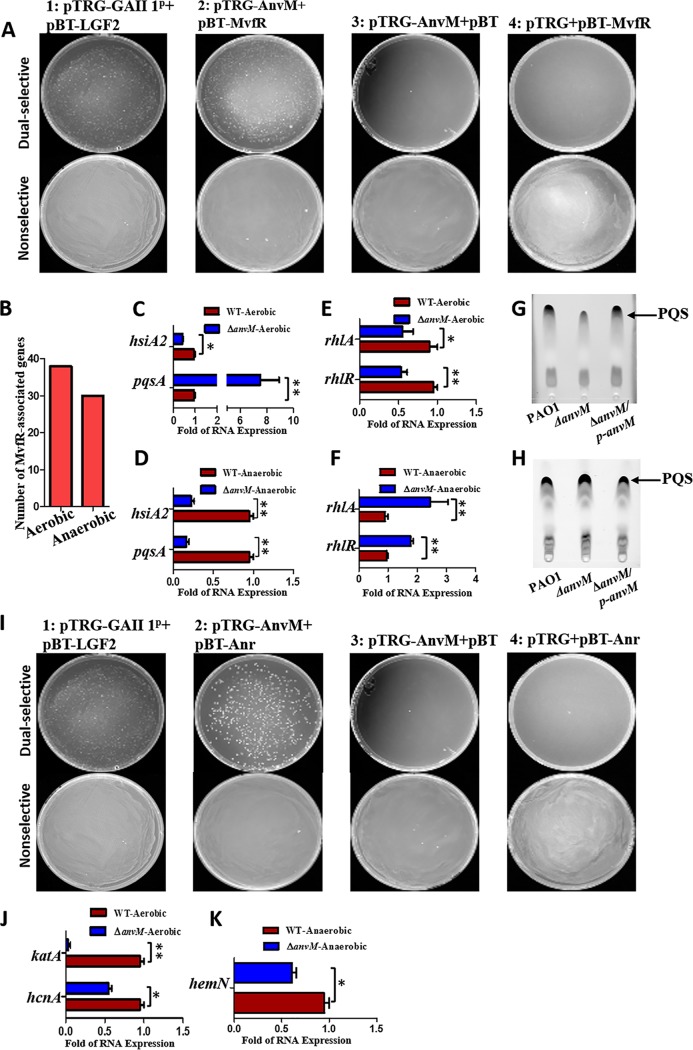
AnvM interacted with MvfR and Anr to modulate virulence and oxidative stress resistance. The strain expressing LGF^2^ and GAI11^P^ was used as a positive control. (A) Bacterial two-hybrid assay revealed an interaction between AnvM and MvfR. The recombinant strain harboring different proteins was separately streaked on nonselective and dually selective media (3-amino-1,2,4-triazole+streptomycin). (B) Statistics of MvfR-regulated genes in RNA-seq results determined for strain Δ*anvM* versus PAO1. (C) mRNA expression levels of MvfR-regulated genes under the aerobic condition. AnvM suppressed expression of the *pqsA* gene and promoted expression of the *hsiA* gene as determined using RT-qPCR analysis. (D) RT-qPCR was performed to measure the transcription levels of the *pqsA* and *hisA* genes and showed that AnvM facilitated the expression of the *pqsA* and *hisA* genes under anaerobic conditions. (E) RT-qPCR was performed to measure the transcription levels of the *rhlR* and *rhlA* genes and showed that AnvM negatively regulated expression of the *rhlR* and *rhlA* genes under aerobic conditions. (F) RT-qPCR was performed to measure the transcription levels of the *rhlR* and *rhlA* genes and showed that AnvM positively regulated of the *rhlR* and *rhlA* genes under anaerobic conditions. (G) The level of production of PQS was lower in strain Δ*anvM* under aerobic conditions. Left to right lines represent the wild type PAO1, strain Δ*anvM* and the Δ*anvM* complemented strain, respectively. (H) The production of PQS was higher in strain Δ*anvM* under anaerobic conditions. Lanes 1 to 3 (left to right) represent wild-type PAO1, strain Δ*anvM*, and the Δ*anvM* complemented strain, respectively. (I) Bacterial two-hybrid assay revealed an interaction between AnvM and Anr. The recombinant strain harboring different proteins was separately streaked on nonselective and dually selective media. (J) mRNA expression levels of Anr-regulated genes under aerobic conditions. AnvM promoted expression of the *katA* and *hcnA* genes as determined using RT-qPCR analysis. (K) RT-qPCR was performed to measure the transcription levels of the *pqsA* and *hcnA* genes and showed that AnvM facilitated the expression of *hemN* gene under anaerobic conditions. The experiments were repeated at least three times, and similar results were observed. *, *P* < 0.05; **, *P* < 0.01; ***, *P* < 0.001 (comparing the Δ*anvM* strain to the wild-type strain using Student's *t* test). Data are representative of results from three independent experiments.

To further study how AnvM modulates P. aeruginosa pathogenicity, we exploited the glutathione *S*-transferase (GST) pulldown assay to screen potential proteins interacting with AnvM (Table S2A). This assay identified MvfR as a potential binding partner; mass spectrometry (MS) confirmed this finding. Given that AnvM regulated the *pqs* system in our RNA-seq experiment, we focused on MvfR because it is a key regulator for this system and a primary virulence factor in P. aeruginosa. A recent study also showed that AnvM is significantly induced by a natural quorum sensing inhibitor, protoanemonin (21), strongly suggesting that AnvM might interact with MvfR. To determine their potential interactions, we performed a bacterial two-hybrid assay by transforming pTRG-AnvM and pBT-MvfR into a BacterioMatch II validation reporter cell (Agilent) to detect the expression levels of the recombinant strains. Strain 1 expressing LGF2 and GAL11^p^ was the positive control that grew on both nonselective and dually selective media. Recombinant strain 2 (bearing pTRG-AnvM and pBT-MvfR) also was separately streaked on both nonselective and dually selective media (Fig. 2A), with results indicating a direct interaction between AnvM and MvfR.

10.1128/mBio.01362-19.10 TABLE S2 LC-MS analysis and expression of MvfR as well as Anr-associated genes. (A) LC-MS analysis of compounds bound to AnvM. (B) Expression of MvfR-associated genes in PAO1 and strain Δ*anvM* under aerobic conditions. (C) Expression of MvfR-associated genes in PAO1 and strain Δ*anvM* under anaerobic conditions. (D) Expression of Anr-associated genes in PAO1 and the PA3880 mutant under anaerobic conditions. Download Table S2, DOCX file, 0.03 MB. Copyright © 2019 Zhang et al. 2019 Zhang et al. This content is distributed under the terms of the Creative Commons Attribution 4.0 International license.

Among the differentially expressed genes analyzed in strain Δ*anvM* via RNA-seq (Tables S2B and C), we found that 37 MvfR-regulated genes were expressed under aerobic conditions and that 29 MvfR-regulated genes were expressed under anaerobic conditions (22) (Fig. 2B). The RT-qPCR verification showed that the level of *pqsA* expression was ∼7-fold higher in strain Δ*anvM* than in the WT strain under aerobic conditions. The expression level of *pqsA* was 4-fold lower in the Δ*anvM* strain than in the WT strain under anaerobic conditions. However, the expression level of the *hsiA* gene was >2-fold lower in strain Δ*anvM* than in the WT strain under both sets of growth conditions (Fig. 2C and D).

To further explore the link between AnvM and the QS system, we tested the expression levels of other QS-associated genes in the Δ*anvM* strain by RT-qPCR. The expression levels of both *rhlR* and *rhlA* were ∼2-fold lower in strain Δ*anvM* than in the WT strain under aerobic conditions (Fig. 2E). However, their expression levels were approximately 2-fold to 3-fold higher in strain Δ*anvM* than in the WT strain grown in anaerobic conditions (Fig. 2F). We further measured the levels of QS molecules, including PQS, C12-HSL, and C4-HSL, in the WT, Δ*anvM*, and complemented strains. The results showed that the level of PQS production was lower in strain Δ*anvM* than in the other two strains under aerobic conditions (Fig. 2G) but that there was no difference between the levels of C12-HSL and C4-HSL production (Fig. S4A and B). PQS production was very low under anaerobic growth conditions (Fig. S4C) but was higher in the Δ*anvM* strain than in the WT under microaerobic conditions (Fig. 2H).

10.1128/mBio.01362-19.4 FIG S4 Production of C12-HSL, C4-HSL, and PQS. (A) Relative amounts of C12-HSL measured by the use of pKD-*lasI* plus pMCSG19-*lasR* in a DH5α system. (B) Relative amounts of C4-HSL measured by the use of pKD-*rhlI* plus pMCSG19-*rhlR* in a DH5α system. (C) Production of PQS under anaerobic conditions. Download FIG S4, TIF file, 0.4 MB. Copyright © 2019 Zhang et al. 2019 Zhang et al. This content is distributed under the terms of the Creative Commons Attribution 4.0 International license.

### AnvM interacted with the anaerobic regulator *anr* to modulate oxidative stress resistance and virulence.

Although a conserved Anr-binding motif was found in the promoter region of *anvM*, an electrophoretic mobility shift assay (EMSA) showed no interaction between them (Fig. S5) (23). The AnvM-GST pulldown assay indicated that AnvM might directly bind to Anr, a protein that senses oxygen availability (24). We speculated that AnvM interacts with Anr to regulate anaerobic metabolism, oxidative stress response, and virulence. To investigate this speculation, we performed a bacterial two-hybrid assay to confirm the direct interaction between AnvM and Anr (Fig. 2I). In the AnvM regulon from the RNA-seq analysis, 34 of the genes were Anr-regulated genes (25, 26) (Table S2D). In particular, under aerobic conditions, the expression levels of *hcnA* and *katA* showed 4-fold and 2-fold reductions in strain Δ*anvM* compared to the WT strain (Fig. 2J). The deletion of *anvM* led to an ∼2-fold reduction of *hemN* expression (Fig. 2K).

10.1128/mBio.01362-19.5 FIG S5 Anr did not bind to the promoter of *anvM*. PCR products containing the Anr-box sequence were added to the EMSA reaction mixtures at a concentration of 50 nM each. Anr protein was added to reaction buffer in the lanes in 0, 0.5, 1.0, and 2.0 μM amounts. Download FIG S5, TIF file, 0.5 MB. Copyright © 2019 Zhang et al. 2019 Zhang et al. This content is distributed under the terms of the Creative Commons Attribution 4.0 International license.

### *anvM* deficiency decreased P. aeruginosa virulence.

**FIG 3:**
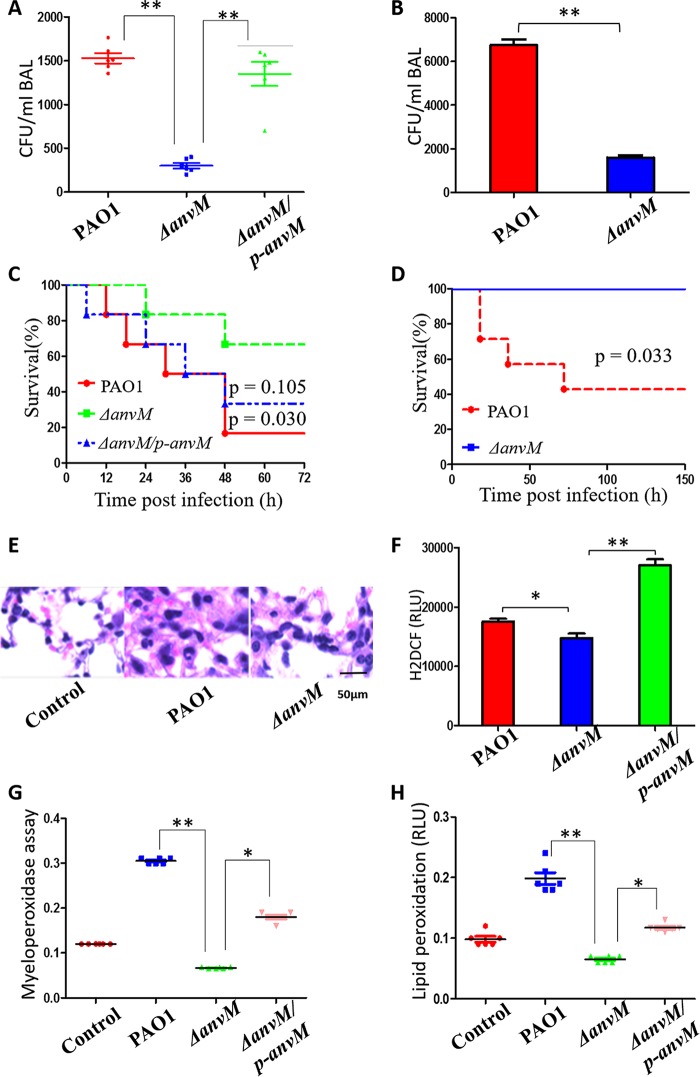
*AnvM* deficiency decreased bacterial virulence and host immune response. (A) Bacterial counts in the lung tissue of the mice infected with the WT strain or strain Δ*anvM* or the corresponding complemented strain (6 mice/group). (B) Bacterial counts in the BAL fluid of the mice infected with either the WT strain or strain Δ*anvM*. (C) Survival rate of these infected mice. Kaplan-Meier survival curves were obtained. *P* = 0.030 (PAO1) or 0.105 (*strain ΔanvM*/*p-anvM*) (log rank test; 95% confidence interval, 1.19 h to 31.8 h [PAO1] 0.7426 h to 23.63 h [strain *ΔanvM*/*p-anvM*]). (D) Preinfection of the *anvM* deletion strain led to protection of mice against P. aeruginosa. Kaplan-Meier survival curves were obtained. *P* = 0.033 (log rank test; 95% confidence interval, 1.19 h to 64.3 h). (E) Less-severe lung injury in strain Δ*anvM*-infected mice than in WT-infected mice. (F) H_2_DCF assay (RLU, relative light units) determining the oxidative stress-generated free radical. (G) Myeloperoxidase assay for determining the oxidative burst in primary alveolar macrophages from infected or control mice. (H) Lipid peroxidation assay in tissues of mice infected by with the WT strain, strain Δ*anvM*, and the corresponding complemented strain. *, *P* < 0.05; **, *P* < 0.01; ***, *P* < 0.001 (one-way ANOVA with Tukey *post hoc* test).

Given that several virulence genes were downregulated via strain Δ*anvM*, we attempted to use a mouse model to test the virulence of the WT strain, strain Δ*anvM*, and the corresponding complemented strain. We intranasally inoculated the bacterial strains into C57BL/6J mice at a multiplicity of infection (MOI) of 5 × 10^6^ CFU/mouse. After 5 days, the Δ*anvM* strain displayed bacterial counts that were only 1/8 the levels of those seen with the WT strain in the infected lung tissue (Fig. 3A) and was complemented by reintroducing the *anvM* gene into the Δ*anvM* strain. The same pattern was also observed in the bronchoalveolar lavage fluid (BALF) (Fig. 3B). Less than 20% of the mice infected with WT PAO1 were alive at 48 h postinfection, which is significantly different from the survival rate of 70% seen with the mice infected with the Δ*anvM* strain (Fig. 3C). More interestingly, we preinfected (vaccinated) mice using either the WT strain or strain Δ*anvM* at reduced doses and then challenged these mice with the WT PAO1 strain. None of the mice that had been preinfected with the Δ*anvM* strain died, while only 30% of mice preinfected with the WT were alive 5 days after the second infection (Fig. 3D). These results strongly indicate that the Δ*anvM* strain is a potential candidate for use as a live attenuated vaccine for preventing P. aeruginosa infection.

### *anvM* deficiency decreased the innate host immune response.

To determine the functional role of AnvM in influencing the host immune response, we used a mouse model to determine differences in host-pathogen interactions among the WT strain, strain Δ*anvM*, and the corresponding complemented strain. We observed a less severe level of lung injury in strain Δ*anvM*-infected mice than in WT-infected mice (Fig. 3E). H_2_DCF fluorescent and myeloperoxidase (MPO) assays demonstrated that alveolar macrophages (AMs) of strain Δ*anvM*-infected mice exhibited an approximately 2-fold decrease in oxidative stress at 24 h postinfection compared to WT-infected mice (Fig. 3F and G). We also examined lipid peroxidation, which was found to be decreased in strain Δ*anvM*-infected mouse lungs at 24 h postinfection compared to the WT-infected mice (Fig. 3H). These AnvM-controlled phenotypes were complemented by reintroducing *anvM* in strain Δ*anvM*. These results demonstrate that AnvM played important roles in impacting the host immune response.

### *anvM* deficiency decreased bacterial phagocytosis by host alveolar macrophages.

To determine the role of AnvM in the interaction between P. aeruginosa and AM, we first analyzed bacterial clearance and phagocytosis of the WT strain, strain Δ*anvM*, and the corresponding complemented strain. The Δ*anvM* strain displayed 3-fold-higher clearance and 3-fold-lower phagocytosis levels than the other two strains (Fig. S6A and B), which is consistent with other phenotypes in terms of pathology and host immune response. We further investigated the interaction between AnvM and cells of the MH-S macrophage cell line by measuring levels of lipid peroxidation, MPO, and phagocytosis (Fig. S6E and F; see also Fig. S7A and B). Notably, we found that *anvM* can be related to inducing autophagy, as its deletion reduced levels of formation of LC-3B puncta (Fig. S7C). Given that AM produces ROS stress, we further tested the function of Cys44 in AnvM-AM interaction. The complementation strain with AnvM-C44S (Cys44 was mutated to Ser) had significantly reduced levels of phagocytosis and ROS production in AM, suggesting that the Cys44 is a critical site for the full function of AnvM (Fig. S6C and D). These results indicate that AnvM is a key gene in the interaction between P. aeruginosa and host cells.

10.1128/mBio.01362-19.6 FIG S6 AnvM deficiency decreased bacterial phagocytosis by host alveolar macrophages. (A and B) AM clearance (A) and phagocytosis (B) of the WT strain or the Δ*anvM* strain or the corresponding complemented strain. (C and D) AM phagocytosis (C) and NBT assay (D) of *anvM*/p-*anvM* and *anvM*/p-*anvM*-C44S strains. (E) Myeloperoxidase (MPO) activity assay. MH-S cells were infected by different bacteria for 3 h (bacterium-to-cell ratio [MOI], 10:1), and cell culture supernatant was collected to detect myeloperoxidase (MPO) activity using a colorimetric kit (Abcam) according to the manufacturer's guidelines. (F) Lipid peroxidation malondialdehyde (MDA) assay. MH-S cells were infected as described for panel A and collected to measure lipid peroxidation malondialdehyde (MDA) levels in cell lysates using a colorimetric kit (Abcam) according to the manufacturer’s instructions. *, *P* < 0.05; **, *P* < 0.01; ***, *P* < 0.001 (one-way ANOVA with Tukey *post hoc* test). Download FIG S6, TIF file, 0.6 MB. Copyright © 2019 Zhang et al. 2019 Zhang et al. This content is distributed under the terms of the Creative Commons Attribution 4.0 International license.

10.1128/mBio.01362-19.7 FIG S7 Phagocytosis assay. (A and B) MH-S cells were seeded in 6-well plates and grown overnight. The cells were treated with serum-free medium for 1 h at 37°C and then infected with PAO1, strain Δ*anvM*, and strain Δ*anvM*/p-*anvM* (10:1 MOI). The wells were washed with PBS two times and treated with 100 μg/ml polymyxin B for 1 h to kill any remaining extracellular bacteria. The cells were again washed in PBS twice and lysed in 0.2% Triton X-100 (Sigma Aldrich). The bacterial CFUs were counted by plating samples onto LB agar plates (panel A, images; panel B, quantification). (C) MH-S cells were infected by different bacteria for 3 h (bacterium-to-cell ratio, MOI 10:1) and washed with PBS three times. The cells were then fixed by the use of 4% paraformaldehyde for 10 min and permeated with Triton X-100. The cells were then incubated with primary anti-LC3B Abs (NOVOS), washed with PBS 5 times, and reacted with secondary antibodies conjugated with Alex Fluor (Invitrogen). DAPI (4′,6-diamidino-2-phenylindole) (Sigma-Aldrich; catalog no. D9542) was used to stain the nucleus, and cells were imaged using an LSM500 fluorescence microscope to evaluate puncta of LC3B (bar, 10 μm). Download FIG S7, TIF file, 1.4 MB. Copyright © 2019 Zhang et al. 2019 Zhang et al. This content is distributed under the terms of the Creative Commons Attribution 4.0 International license.

### AnvM elicited a specific immune pathway in host cells.

**FIG 4:**
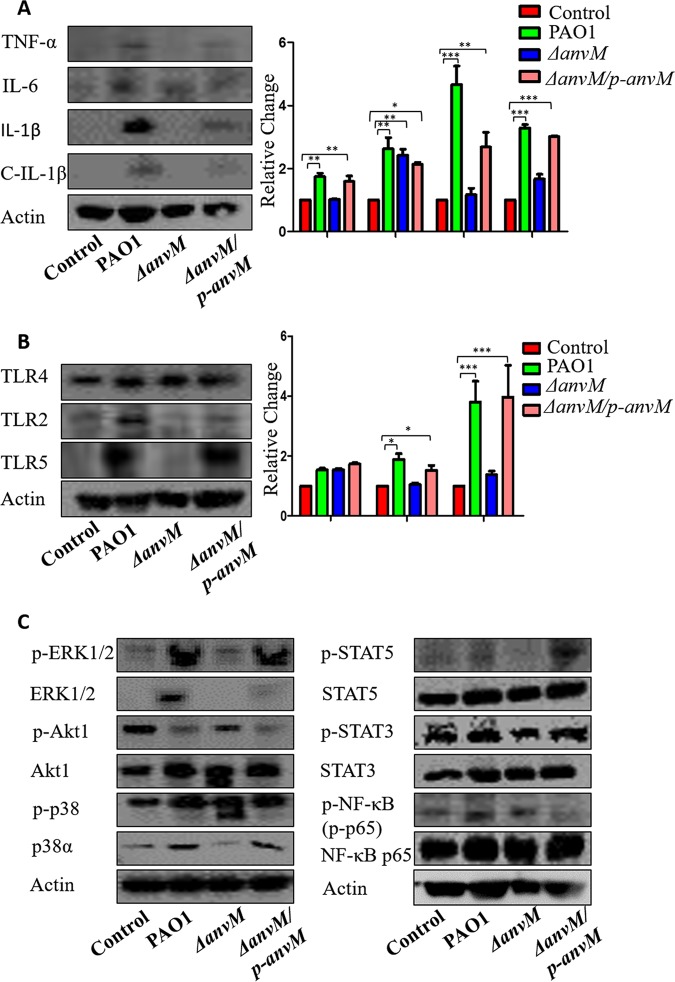
AnvM-dependent immune pathway in host cells. Western blotting for inflammatory cytokines (A), cleaved IL-1β (C-IL-1β) for TLRs as immune activators (B), and various cell signaling pathways (C) detected in lung tissue homogenates of C57B/L6J mice at 24 h postinfection of the WT strain, strain Δ*anvM*, and the corresponding complemented strain. Gel data were quantified using densitometry with Quantity One, and data are representative of results from three independent experiments. See Fig. S2 for quantification of panel C data. *, *P* < 0.05; **, *P* < 0.01; ***, *P* < 0.001 (one-way ANOVA with Tukey *post hoc* test).

To evaluate the profile of inflammatory cytokines, we performed immunoblotting to determine the expression patterns of several inflammatory cytokines in mouse lung samples. Tumor necrosis factor alpha (TNF-α) and interleukin-1β (IL-1β) levels were highly induced following infection with the WT strain compared to infection with strain Δ*anvM* (Fig. 4A). IL-6 levels were also elevated after PAO1 infection, but there were no significant differences among the WT strain and strain Δ*anvM* and its complementary group (Fig. 4A), indicating that *anvM* might play a role in regulating the expression of proinflammatory cytokines TNF-α and IL-1β.

Pattern recognition receptors (PRR), such as Toll-like receptor 2 (TLR2), TLR4, and TLR5, participate in recognizing invading pathogens and contributing to the activation of inflammatory cytokines through different pathways (27, 28). Our data showed that *anvM* deficiency downregulated the levels of TLR2 and TLR5 compared to the WT strain and complementary strain results (Fig. 4B).

As TLR2 and TLR5 enhance the expression of cytokines via the p38 and NF-κB pathways (29, 30), we used immunoblotting to detect the expression of p38, p-p38, p65(NF-κB), and p-p65. A quantitative analysis revealed that *anvM* deficiency reduced the protein level of p38 whereas the levels of p-p38, p65, and p-p65 were not significantly changed among the 3 strains (Fig. 4C; see also Fig. S8D and F), indicating that AnvM regulates the expression of TNF-α and IL-1β via the TLR2/TLR5/p38 pathway.

10.1128/mBio.01362-19.8 FIG S8 Gel data (Fig. 4C) were quantified using densitometry with Quantity One (three independent experiments). *, *P* < 0.05; **, *P* < 0.01; ***, *P* < 0.001 (one-way ANOVA with Tukey *post hoc* test). Download FIG S8, TIF file, 0.9 MB. Copyright © 2019 Zhang et al. 2019 Zhang et al. This content is distributed under the terms of the Creative Commons Attribution 4.0 International license.

We also detected the expression of other potential immune effectors and inflammatory responders (31–33), including extracellular signal-regulated kinase 1 (ERK1)/ERK2 (ERK1/2), Akt, STAT3, and STAT5. We found that only ERK1/2 and p-ERK1/2 were upregulated by AnvM (Fig. S8A to C and E), indicating that ERK1/2 and its phosphorylation were also involved in the immune response to AnvM-mediated virulence. However, the changes corresponding to Akt and pAkt were not oriented in the same direction as the Akt increase but did match the pAkt decrease in strain Δ*anvM* compared to the WT strain. The pSTAT5 level was significantly increased in strain Δ*anvM* compared to the WT strain. Taken together, our data suggest that TLR2-STAT5-ERK1/2 could be associated with the immune defense against Δ*anvM* mutation.

### AnvM directly bound to TLR2 and TLR5 and regulated genes associated with flagella and LPS.

**FIG 5:**
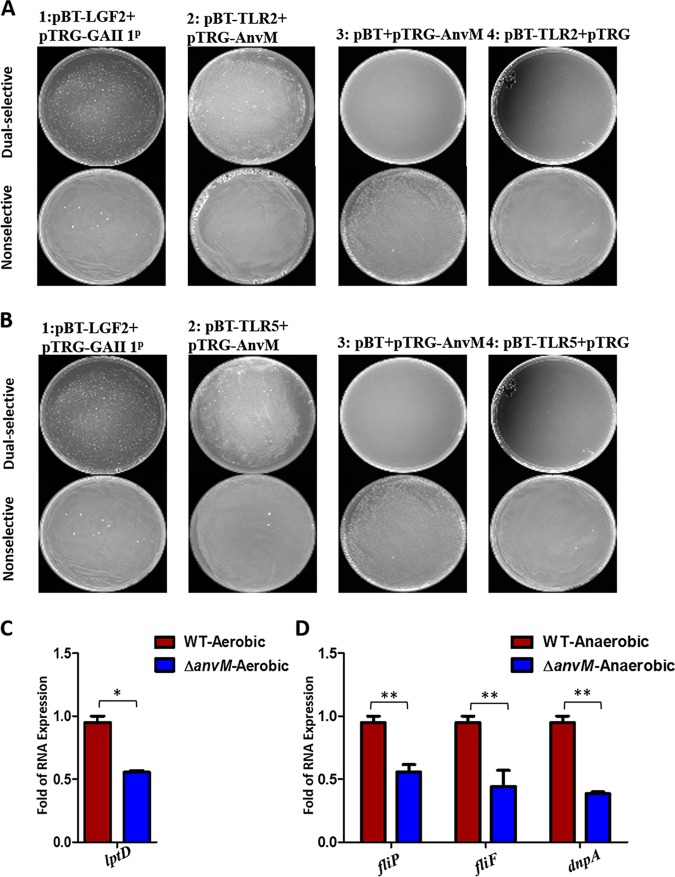
AnvM directly bound to TLR2/5 and regulated genes associated with flagella as well as LPS. (A and B) A bacterial two-hybrid experiment demonstrated an interaction between AnvM and TLR2 or TLR5. The recombinant strain harboring different proteins was separately streaked on nonselective and dually selective media. The strain expressing LGF2 and GAI11^P^ was used as a positive control. (C) RT-qPCR was performed to measure the transcription level of *lptD* under aerobic conditions. (D) RT-qPCR revealed that AnvM spurred expression of *flip*, *fliF*, and *dnpA*. The experiments were repeated at least three times, and similar results were observed. *, *P* < 0.05; **, *P* < 0.01; ***, *P* < 0.001 (comparing the Δ*anvM* strain with the wild-type strain using Student's *t* test). Data are representative of results from three independent experiments.

As AnvM positively regulated the expression of TLR2 and TLR5 in the host (Fig. 4B), we posited that AnvM directly bound to TLR2 and TLR5. Using a bacterial two-hybrid system, we found that two versions of strain 2 (the first bearing pTRG-AnvM and pBT-TLR2 and the second bearing pTRG-AnvM and pBT-TLR5) grew on a dually selective medium, indicating that AnvM interacted with TLR2 and TLR5 (Fig. 5A and B). As TLR2 and TLR5 are induced by bacterial lipopolysaccharide (LPS) and flagella, respectively (23, 34–39), we proposed that AnvM regulates LPS and flagellum biosynthesis. We thus tested whether AnvM regulated the expression of LPS-associated genes (*lptD* and *dnpA*) and flagellum-associated genes (*fliP* and *fliF*) using RT-qPCR. As shown in Fig. 5C, the mRNA levels of *lptD* were 2-fold lower in strain Δ*anvM* than in the WT strain, suggesting that AnvM positively regulated *lptD* transcription under aerobic conditions. Finally, the expression levels of the *dnpA*, *flip*, and *fliF* genes were reduced by approximately 2-fold in strain Δ*anvM*, indicating that AnvM positively regulated *dnpA*, *flip*, and *fliF* under anaerobic conditions (Fig. 5D). These observations demonstrate that AnvM not only directly binds to TLR2 and TLR5 in hosts but also positively tunes genes associated with flagella and LPS.

## DISCUSSION

To explore whether AnvM was widely distributed in the *Bacteria* kingdom, a phylogenetic analysis was used. It revealed that AnvM homologs were widely distributed among bacteria. AnvM is composed of 131 amino acids, including a DGC conservative sequence that is predicted to be a binding site of zinc. To demonstrate this, we attempted to purify the AnvM protein and tested its binding affinity to zinc and other metal ions. Unfortunately, after many attempts, AnvM was always found to be present in the inclusion body of various Escherichia coli expression systems (data not shown). After dissolving the AnvM inclusion body, we did not detect a significant concentration of Zn or Cu or Fe in the AnvM protein. To further explore the AnvM-regulated pathogenic mechanism, we performed a pulldown assay to search for its binding proteins, which revealed MvfR, LasB, and Anr (see Table S2A in the supplemental material). A mass spectrometry analysis showed the presence of many unknown proteins along with a few known proteins, such as MvfR, LasB, and Anr and a group of metabolic enzymes. MvfR and LasB are involved in bacterial virulence, and Anr is a regulator of anaerobic metabolism. These proteins are closely related to the phenotypes of strain Δ*anvM*. Our bacterial two-hybrid analyses showed that AnvM interacted with MvfR and Anr (Fig. 2A and I) but not with LasB and LasR (the negative control) (see Fig. S2D and E in the supplemental material).

**FIG 6:**
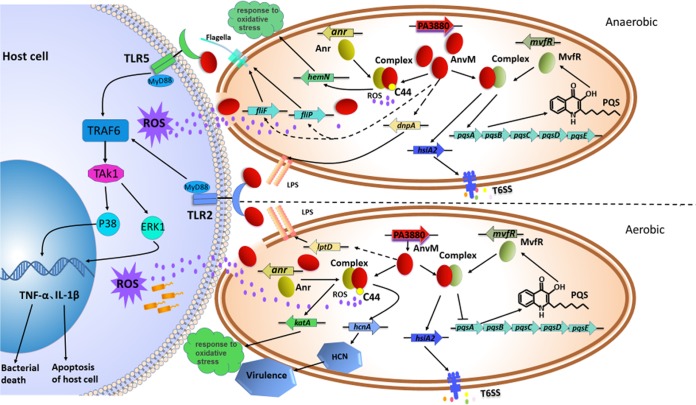
Model of AnvM-mediated regulation and its interaction with host cell. The regulatory pathways of AnvM are proposed based on our current observations and previous studies. In the present study, we demonstrated that AnvM interacted with MvfR and Anr. Under anaerobic conditions, AnvM is expressed at a high level and interacts with MvfR to promote the expression of *hsiA* and *pqsA*, thus activating the T6SS and QS system. *hemN* was positively regulated by AnvM after interacting with Anr, which senses oxygen levels and responds to oxidative stress. AnvM positively regulates *fliF*, *flip*, and *dnpA* to synthesize flagella as well as LPS, which leads to activation of TLR5 and TLR2 to turn on the pathways of p38 and ERK1. Under aerobic conditions, AnvM interacts with MvfR, which induces expression of *hsiA* to regulate the T6SS and represses expression of *pqsA* to regulate the QS system. *katA* and *hcnA* are positively regulated by the AnvM-Anr complex. AnvM positively regulates *lptD* to regulate LPS, which activates the p38 or ERK1 pathway of TLR2. In addition, Cys44 is a critical site for AnvM function, which senses ROS produced by the host immune system. AnvM also directly interacts with TLR2 and TLR5 to activate the pathways of p38 or ERK1, which causes host immune response. Solid arrows indicate positive regulation, and solid T-bars present negative regulation. Solid lines or dotted lines represent direct or indirect regulatory relationships that were established, respectively.

Although AnvM did not bind with a conserved Anr-binding motif in the promoter region of *anvM* (23), AnvM did interact with Anr, which could explain why *anvM* was not regulated by Anr (26). We found that the promoter regions of *hcnA* (encoding hydrogen cyanide synthase [40]), *katA* (encoding catalase A [41]), and *hemN* (encoding an oxygen-independent dehydrogenase) had an Anr-box in their promoters. We then tested whether AnvM regulates these 3 genes via Anr. The RT-qPCR results showed that AnvM regulated the expression level of the *hcnA* gene to affect virulence under aerobic conditions. Furthermore, AnvM positively regulated the expression levels of *katA* and *hemN*, indicating that AnvM binds to Anr, thus regulating oxidative stress resistance by tuning *katA* and *hemN* (Fig. 6).

Consistent with the RNA-seq analysis, the *in vivo* experiments demonstrated that the virulence and host immune response of the Δ*anvM* strain were both significantly compromised. Compared to the WT strain, the Δ*anvM* strain displayed reduced phagocytosis by AM, which strongly suggests that AnvM played an important role in ROS sensing and response. Given that the point mutation Cys44Ser suppressed the *in vivo* function of AnvM, Cys44 could represent a functional active site of AnvM in ROS sensing. AnvM might also directly influence the interaction between P. aeruginosa and macrophages.

To elucidate how the host immune system responds to the *anvM* gene, we examined the levels of several proinflammatory cytokines and noted that TNF-α and IL-1β were inhibited by *anvM* deficiency, while the IL-6 level was not altered. These results suggest that AnvM might participate in the regulation of TNF-α and IL-1β expression. We also observed that expression of TLR2 and TLR5 was activated by AnvM. Interestingly, this finding differs from some of our previous reports showing that TLR4, rather than TLR2, is mostly responsible for P. aeruginosa infection (27, 28). Although Akt1 and p38 are also involved in this virulence factor-mediated immunity, the TLR2/STAT5/ERK1/2 pathway are more intensely involved. Taken together, our findings suggest that AnvM regulates the expression of TNF-α and IL-1β via the TLR2/STAT5/ERK1/2 pathways, which is unique compared to some of our previous observations (29, 30).

We did not observe a growth defect of the Δ*anvM* strain compared to the WT strain under conditions of anaerobic growth (data not shown), suggesting that AnvM does not affect bacterial fitness. Further study of the mechanism of an AnvM-mediated immune response showed that AnvM directly interacted with TLR2 in hosts. We propose that AnvM is released when the host immune response breaks the bacterial cell wall and then binds to TLR2, which leads to activation of the STAT5 and ERK1 pathways, which eventually induce the inflammatory responses (Fig. 6). In addition, AnvM positively regulated *lptD*, *dnpA*, *flip*, and *fliF* (Fig. 5C and D; see also Fig. 6), which are involved in the biosynthesis of LPS and flagella that can induce TLR2 pathways in host cells.

As a newly identified member of the regulatory proteins, AnvM is required for effective bacterial infections by controlling hundreds of genes and is also important in regulating the interaction between bacteria and host immune systems. AnvM has profound and multilayered regulatory functions, such as binding to MvfR, Anr, TLR2, and TLR5. It could be a critical regulator of bacterial physiology and the host response and thus represents a potential therapeutic target. This work provides novel details about the bacterial response to oxidative stress, virulence, and the host response to inflammation and might offer new insight into the regulatory and functional aspects of the interaction between P. aeruginosa and host cells.

## MATERIALS AND METHODS

### Strains, plasmids, primers, and growth conditions.

The bacterial strains, plasmids, and primers used in this study are listed in Table S1A and B in the supplemental material. P. aeruginosa PAO1 and derivatives were grown at 37°C on lysogeny broth (LB) agar dishes or in broth with shaking at 220 rpm. Anaerobic culturing was performed in 100-ml hermetically sealed bottles containing an AnaeroPack-Anaero (MGC). Bottles were incubated at 37°C with shaking at 220 rpm. The oxygen initially present in the bottle was consumed by AnaeroPack-Anaero. Micro-oxygen culturing was performed in 100-ml hermetically sealed bottles containing an AnaeroPack-MicroAero (MGC). Bottles were incubated at 37°C with shaking at 220 rpm. The oxygen initially present in the bottle was consumed by AnaeroPack-MicroAero until the concentration of oxygen became 6% to 12% and the concentration of carbon dioxide became 5% to 8%. Antibiotics were used at the following concentrations: for E. coli, gentamicin (Gm) at 15 μg/ml, ampicillin at 100 μg/ml, and tetracycline at 10 μg/ml; for P. aeruginosa, gentamicin (Gm) at 50 μg/ml in LB; tetracycline at 150 μg/ml in LB or 300 μg/ml in *Pseudomonas* isolation agar (PIA), and carbenicillin at 500 μg/ml in LB.

### Construction of P. aeruginosa *anvM* deletion mutant.

For construction of gene knockout mutants, a SacB-based strategy was employed as described in a previous study (42) and the current study. PCRs were performed to amplify sequences 2 kb upstream and 2 kb downstream of the intended deletion (Table S1B). The two PCR products were cloned into vector pEX18Ap, yielding pEX18Ap-*anvM*. A 0.9-kb gentamicin resistance cassette cut from pPS858 with XbaI was cloned into pEX18Ap-*anvM*, yielding pEX18Ap-*anvM*-Gm. The resultant plasmids were electroporated into PAO1 with selection for gentamicin resistance. Colonies showing both gentamicin resistance and loss of sucrose (5%) susceptibility were selected on LB agar plates containing 50 μg/ml of gentamicin and 5% sucrose, which typically indicates a double-crossover event and thus the occurrence of gene replacement.

### Phylogenetic tree construction of AnvM-like proteins.

The distribution of AnvM homologs in microorganisms was studied using BLAST in NCBI. A total of 1,000 homologous sequences were selected with E value lower than the threshold of 5E−08. We chose 30 homologous among them. The distance tree was generated using BLAST pairwise alignments in NCBI. The phylogenetic tree of AnvM-like proteins was constructed using MEGA7 software. The phylogenetic trees were modified using online software (http://itol.embl.de/).

### Transcriptome analysis.

The wild-type and Δ*anvM* strains were cultured in LB medium until an optical density at 600 nm (OD_600_) of 0.6 was reached. Then, 2-ml volumes of bacterial cultures were collected by centrifugation (12,000 rpm, 4°C). RNA purification was conducted by the use of an RNeasy minikit (Qiagen). After removal of rRNA by the use of a MICROBExpress kit (Ambion), mRNA was used to generate the cDNA library according to the NEBNext Ultra II RNA library prep kit protocol (NEB), which was then sequenced using a HiSeq 2000 system (Illumina). Bacterial RNA-seq reads were mapped to the Pseudomonas aeruginosa PAO1 genome (NC_002516) by using STAR. Only the uniquely mapped reads were kept for the subsequent analyses. The gene differential expression analysis was performed using Cuffdiff software (version 2.0.0) (43 ). GO enrichment analyses were conducted on all differentially transcribed genes using DAVID (44 ). Each sample analysis was repeated twice. The RNA-seq data sets have been submitted to National Center for Biotechnology Information (NCBI).

### RT-PCR.

RT-PCR was performed by the use of a FastKing RT kit (Tiangen Biotech). We used 1 μg total RNA as the template and prepared the mixture according to the instruction of manufacturer. The mixed liquor was incubated for 15 min at 42°C and then for 3 min at 95°C.

### MIC measurements.

MICs of H_2_O_2_ and cumene hydroperoxide (CHP) were measured in LB medium by using a microdilution technique according to NCCLS guidelines. LB medium was used to grow P. aeruginosa in a 96-well plate. The MIC value was recorded as the lowest concentration at which there was no visible growth of P. aeruginosa.

### Cytoplasm and membrane protein purification.

The constructed plasmids were cultured in LB medium with ampicillin at 37°C. When the OD_600_ reached 0.6 to 0.8, the protein was induced with 0.5 mM isopropyl-β-d-1-thiogalactopyranoside (IPTG). The induced cultures were then grown at 16°C about 16 to 20 h. Bacterial cells were harvested by centrifugation (5,000 rpm for 20 min) at 4°C and washed with 1× phosphate-buffered saline (PBS). The acquired cell pellets were resuspended in buffer A (20 mM Tris-HCl [pH 8.0], 100 mM NaCl, 5 mM DNase I, 1 mM phenylmethylsulfonyl fluoride [PMSF], 2 mM MgCl_2_) to 20% (wt/vol), lysed by a single passage through a French press (JN-Mini, China) (at 500 lb/in^2^ once and 1,300 lb/in^2^ twice), and centrifuged at 16,800 rpm for 1 h at 4°C for collection of the supernatant. Subsequently, the supernatant was subjected to spinning at 38,000 rpm for 1 h at 4°C (cytoplasm protein), and the precipitant obtained was dissolved in buffer B (20 mM Tris-HCl [pH 8.0], 100 mM NaCl, 5% glycerol, 1% [mass/vol] n-dodecyl-d-maltoside [DDM]). Finally, the supernatant was collected after centrifugation at 38,000 rpm for 1.5 h at 4°C (membrane protein).

### Protein purification.

The constructed plasmids were cultured in LB medium with kanamycin at 37°C. When the OD_600_ reached 0.6 to 0.8, the protein was induced with 0.5 mM isopropyl-β-d-1-thiogalactopyranoside (IPTG). The induced cultures were then grown at 37°C for 3 to 4 h. The His-tagged protein was purified using nickel-nitrilotriacetic acid (Ni-NTA) beads. And the protein was identified by SDS-PAGE and immunoblotting.

### Electrophoretic mobility shift assay (EMSA).

Briefly, AnvM or Anr proteins were mixed with DNA probes (20) following the instructions provided with an EMSA kit (Thermo Fisher Scientific) and were incubated at room temperature for 20 min. The samples were then analyzed by 5% polyacrylamide gel electrophoresis in 0.5× Tris-borate-EDTA (TBE) buffer at 90 V for 90 min. The gels were then stained by the use of SYBR green EMSA nucleic acid gel stain and visualized using Bio-Rad Gel Doc XR+.

### GST pulldown assay.

The GST pulldown assay was performed as previously described (45) with minor modifications. For screening AnvM interaction partners in P. aeruginosa, stationary-phase P. aeruginosa cells grown in LB (250 ml) were collected and lysed by sonication in lysis buffer (10 ml PBS supplemented with 5 mM MgCl_2_, 150 U DNase I, 2 mM PMSF, 2 mM dithiothreitol [DTT], and 1% Triton X-100). Cleared cell lysates were incubated with 40 μg purified GST-AnvM on a rotator at 4°c overnight, and 40-μl volumes of prewashed glutathione-Sepharose beads (Novagen) were added to the reaction mixtures. After another 4 h of incubation at 4°c, the beads were washed five times with TEN buffer (100 mM Tris-Cl [pH 8.0], 10 mM EDTA, 300 mM NaCl). Proteins associated with beads were solubilized with SDS sample buffer, separated by SDS-PAGE, and detected by Coomassie blue staining (Bio-Rad). Gel slices containing individual protein bands were excised, digested with trypsin, and analyzed by matrix-assisted laser desorption ionization–mass spectrometry.

### LC-MS analysis of compounds bound to AnvM.

Bacterial cells were allowed to grow for ∼24 h in Trypticase soy broth (TSB) medium based on their growth status until the OD_600_ reached 1.9 to 2.0. The supernatant of the bacterial culture (5 ml) was extracted with the same volume of ethyl acetate. The ethyl acetate extracts were dried using a SpeedVac concentrator and redissolved in 50 μl of methanol before these were used in next steps. The binding of AnvM (100 μg) to this extract was performed in 500 μl of 10 mM HEPES (pH 7.4)–100 mM NaCl–10 μl of chemical extracts at 4°C for 6 h. As a control, the AnvM protein was replaced by GST in the binding experiment. The binding system was then filtered through a 3K centrifugal filter device (Amicon Ultra-15) to remove the protein. The filtered liquid was dried using the SpeedVac concentrator before addition of 50 μl of methanol. The dissolved part containing the chemicals was analyzed by ultraperformance liquid chromatography (UPLC) (ACQUITY; Waters, USA) coupled with a MicroTOF-MS system (Bruker Daltonics GmbH, Bremen, Germany). Reverse-phase chromatography was performed in BEH C_18_ columns (Waters, USA) (2.1 by 150 mm^2^, 1.7-μm pore size) at a flow rate of 250 μl min^−1^. Samples dissolved in acetonitrile were eluted by the use of a mobile-phase gradient (5% to 95% acetonitrile–0.1% formic acid–water) over 30 min. The raw liquid chromatography-mass spectrometry (LC-MS) data were then analyzed by using software provided by the Bruker Corporation.

### Bacterial two-hybrid assay.

Bacterial two-hybrid experiments were performed using a BacterioMatch II two-hybrid system vector kit (Agilent Technologies). The fragments of Anr and MvfR were cloned into pBT bait vector in order to create a fusion protein with λ repressor protein (λcI). The DNA fragment of AnvM was inserted into the vector pTRG in frame with the α-subunit of RNA polymerase. The pBT-derived plasmids and pTRG-derived plasmid were then cotransformed into the validation reporter strain. Then, the resulting strains, grown at 37°C in Super Optimal broth with catabolite repression (SOC) medium for 90 min, were harvested by centrifugation (5,000 × *g*, 2 min) and washed twice with M9 plus His-dropout broth (1× M9 minimal salts containing 1× His-dropout supplemented amino acids). The cells were spotted on the nonselective screening medium and selective screening medium and were finally incubated at 30°C for 2 to 4 days. The colonies grown on the selective screening medium were selected and streaked on the dual-screening medium for further verification. The resulting cotransformation containing pBT-LGF2 and pTRG-GAI11^P^ plasmids was used as the positive control.

### RNA isolation and real-time quantitative PCR.

For real-time quantitative PCR (RT-qPCR) analysis, all P. aeruginosa strains were grown at 37°C with shaking at 220 rpm until an OD_600_ of 0.6 was reached. To harvest the bacteria, the cultures were centrifuged at 8,000 rpm for 1 min. RNA purification was performed using an RNeasy minikit (Qiagen). RNA concentration was measured by the use of a NanoDrop 2000 spectrophotometer (Thermo Fisher). The cDNA synthesis was performed using a FastKing RT kit (Tiangen Biotech). RT-qPCR was performed by the use of a SuperReal Premix Plus (SYBR green) kit (Tiangen Biotech) according to the instructions of the manufacturer. Each reaction was performed in triplicate in 25-μl reaction volumes with 800 ng cDNA and 16S rRNA as internal controls. In each reaction mixture, 200 nM concentrations of primers (Table S1B) were used for RT-qPCR. The reactions were run at 42°C for 15 min and at 95°C for 3 min, and the reaction mixtures were kept at 4°C until needed. The fold change data represent the relative expression levels of mRNA, which can be estimated by the threshold cycle (2^−ΔΔ^*^CT^*^)^ values. All the reactions were conducted with three repeats.

### Bioassay of C4-HSL and C12-HSL activity.

The procedures were modified from those used in a previously reported study (46). We measured the levels of C4-HSL and C12-HSL activity with E. coli DH5α carrying pKD-*rhlI*-lux, pMCSG19-*rhlR*, pKD-*lasI*-lux, and pMCSG19-*lasR*. The reporter strains were cultured in LB broth (50 μg/ml kanamycin and 100 μg/ml ampicillin) at 37°C for 10 h and diluted to an OD_600_ of 0.05 in fresh LB (kanamycin and ampicillin). PAO1 (pAK1900), strain Δ*anvM* (pAK1900), and strain Δ*anvM* (pAK1900-*anvM*) were cultured in LB broth (100 μg/ml carbenicillin) at 37°C for 10 h and then diluted to an OD_600_ of 0.05 in fresh LB (carbenicillin). A 100-μl volume of diluted reporter culture was mixed with an equal volume of PAO1 (pAK1900) or of strain Δ*anvM* (pAK1900) or of strain Δ*anvM* (pAK1900-*anvM*) culture. Luminescence was measured after 4 h at 37°C with shaking by using a Synergy 2 plate reader (BioTek).

### Quantification of PQS.

PQS production was quantified with a method described previously (47). Overnight cultures were transferred into fresh LB broth (1:100 dilutions) and cultured at 37°C for 12 h. A 1-ml volume of acidified ethylacetate was added into 500 μl of each culture and mixed vigorously by using a vortex vibration meter for 2 min. Then, the mixture was centrifuged at 12,000 rpm for 10 min, and the top layer was transferred to a new tube and volatilized completely. A 50-μl volume of a mixture of acidified ethyl acetate and acetonitrile (1:1 [vol/vol]) was used to dissolve the solute. To separate the extracts described above, 5-μl extracts were loaded and methylene chloride-acetonitrile-dioxane (17:2:1) acted as a solution. Finally, the plate was illuminated with UV light. The signal intensity was measured by the use of Quantity One software (Bio-Rad).

### Mouse experiments.

WT C57BL/6J female mice (6 to 8 weeks of age) were obtained from The Jackson Laboratory. Mice were kept in a specific-pathogen-free facility at the University of North Dakota (UND). The animal procedures have been approved by University of North Dakota Institutional Animal Care and Use Committee (UND IACUC).

### Cells, bacterial strains, and infection.

Mouse alveolar macrophages (AMs) were isolated from bronchoalveolar lavage fluid (BALF). AM cells were grown in RPMI 1640 medium in a 37°C-5% CO_2_ incubator. The P. aeruginosa PAO1 WT strain was kindly provided by S. Lory (Harvard University) (26). The other bacterial strains and plasmids are listed in Table S1A. PAO1 and its derivatives were grown for ∼16 h on lysogeny broth (LB) agar or liquid medium at 37°C with shaking at 220 rpm and were pelleted by centrifugation at 5,000 rpm/4°C. Mammalian cells (in antibiotic-free medium) were infected with bacteria at a multiplicity of infection (MOI) of 20 for 1 h.

### Histological analysis.

Lung sections were fixed in 4% formalin for 24 h and then processed by hematoxylin and eosin staining in AML Laboratories (Baltimore, MD) and were observed using a Nikon 80i Eclipse (upright) microscope. The degree of cellular infiltration was scored using a previously described method. The index was calculated by multiplying severity by extent in 10 random areas, with a maximum passible score of 944.

### Bacterial burden assay.

AMs from BALF and from lung, spleen, liver, and kidney tissues were homogenized with PBS and spread on LB dishes for enumeration of bacterial numbers. The dishes were cultured in a 37°C incubator overnight, and colonies were counted. Triplicate experiments were done for each sample and control (50).

### NBT assay.

AMs from BALF were grown in a 96-well plate in serum-containing medium at 37°C for 4 h with nitroblue tetrazolium (NBT) dye (Sigma-Aldrich, St. Louis, MO) (1 μg/ml) added into each well. Cells were incubated for another 1 h or until color developed. NBT was reduced by ROS to the dark blue formation, which was dissolved in dimethyl sulfoxide (DMSO), and its absorbance was determined at 560 nm. Similarly, we measured H_2_DCF fluorescence to determine the extent of oxidation in AMs (48).

### Phagocytosis assay.

MH-S cells were seeded in 6-well plates and grown overnight. The cells were treated with the serum-free medium for 1 h at 37°C and then infected with PAO1, strain Δ*anvM*, and strain Δ*anvM*/p-*anvM* (10:1 MOI). The wells were washed with PBS two times and treated with 100 μg/ml polymyxin B for 1 h to kill any remaining extracellular bacteria. The cells were again washed in PBS twice and lysed in 0.2% Triton X-100 (Sigma-Aldrich). The bacterial CFU were counted by plating samples to LB agar plates. Furthermore, MH-S cells were infected by different bacteria for 3 h (bacterium-to-cell ratio, MOI 10:1) and washed using PBS for three times. The cells were then fixed in 4% paraformaldehyde for 10 min and permeated using Triton X-100. Then, the cells were incubated with primary anti-LC3B Abs (NOVOS), washed with PBS 5 times, and reacted with secondary antibodies conjugated with Alex Fluor (Invitrogen). DAPI (4′,6-diamidino-2-phenylindole) (Sigma-Aldrich; catalog no. D9542) was used to stain the nucleus, and cells were imaged using an LSM500 fluorescence microscope to evaluate puncta of LC3B.

### Lipid peroxidation assay.

Malondialdehyde (MDA) is an end product of the lipid peroxidation process and was measured in a colorimetric assay (Calbiochem, San Diego, CA) according to the manufacturer's instructions. Homogenized samples placed in 62.5 mM Tris-HCl (pH 6.8) supplemented with Complete mini protease inhibitor (Roche Diagnostics) in equal protein amounts were used in the assay.

### Myeloperoxidase (MPO) assay.

MPO assays were performed as described previously (49). Samples were homogenized in 50 mM hexadecyl trimethyl ammonium bromide (HTAB)–50 mM KH_2_PO_4_ (pH 6.0)–0.5 mM EDTA. Tissue (1 ml/100 mg) was applied, and the homogenate was centrifuged for 15 min at 12,000 rpm and 4°C. Supernatants were decanted, and 100 μl of reaction buffer (0.167 mg/ml O-dianisidine, 50 mM KH_2_PO_4_ [pH 6.0], 0.0005% mM H_2_O_2_) was added to 100 μl of sample. Absorbance was read at 460 nm at 2-min intervals.

### Immunoblotting.

Mouse monoclonal antibodies (STAT3, STAT5, ERK1/2, Akt, NF-κB, IL-6, actin, p-ERK1/2, and p-p38), rabbit polyclonal antibodies (cleaved IL-1β, TLR-2, TLR-4, TLR-5, p38, p-STAT3, p-STAT5, p-Akt1, and p-NF-κB), and goat polyclonal antibodies (TNF-α and IL-1β) were obtained from Santa Cruz Biotechnology (Santa Cruz, CA). The samples derived from cells and lung homogenates were lysed in radioimmunoprecipitation assay (RIPA) buffer, separated by electrophoresis on SDS-PAGE gels, and transferred to nitrocellulose transfer membranes (GE Amersham Biosciences, Pittsburgh, PA). Proteins were detected by Western blotting using primary Abs at a concentration of 1/200 and were incubated overnight (51). Labeling of the first Abs was performed using relevant secondary Abs conjugated to horseradish peroxidase (HRP) (Santa Cruz Biotechnology), which were detected using ECL regents (Santa Cruz Biotechnology) and quantified using Quantity One software (Bio-Rad).

### Statistical analysis.

The microarray analyses were repeated twice. All other experiments were repeated at least three times. Two-tailed Student's *t* tests or one-way analysis of variance (ANOVA) with Tukey *post hoc* tests was performed using Microsoft Office Excel 2011 or GraphPad Prism 5 software.

### Ethics statement.

All animal studies were carried out in accordance with the recommendations in the Guide for the Care and Use of Laboratory Animals of the National Institutes of Health. The protocols were approved by the University of North Dakota Institutional Animal Care and Use Committee and performed in accordance with the animal care and institutional guidelines. Dissections and injections of animals were performed under conditions of anesthesia that was induced and maintained with ketamine hydrochloride and xylazine, and all efforts were made to minimize suffering.
