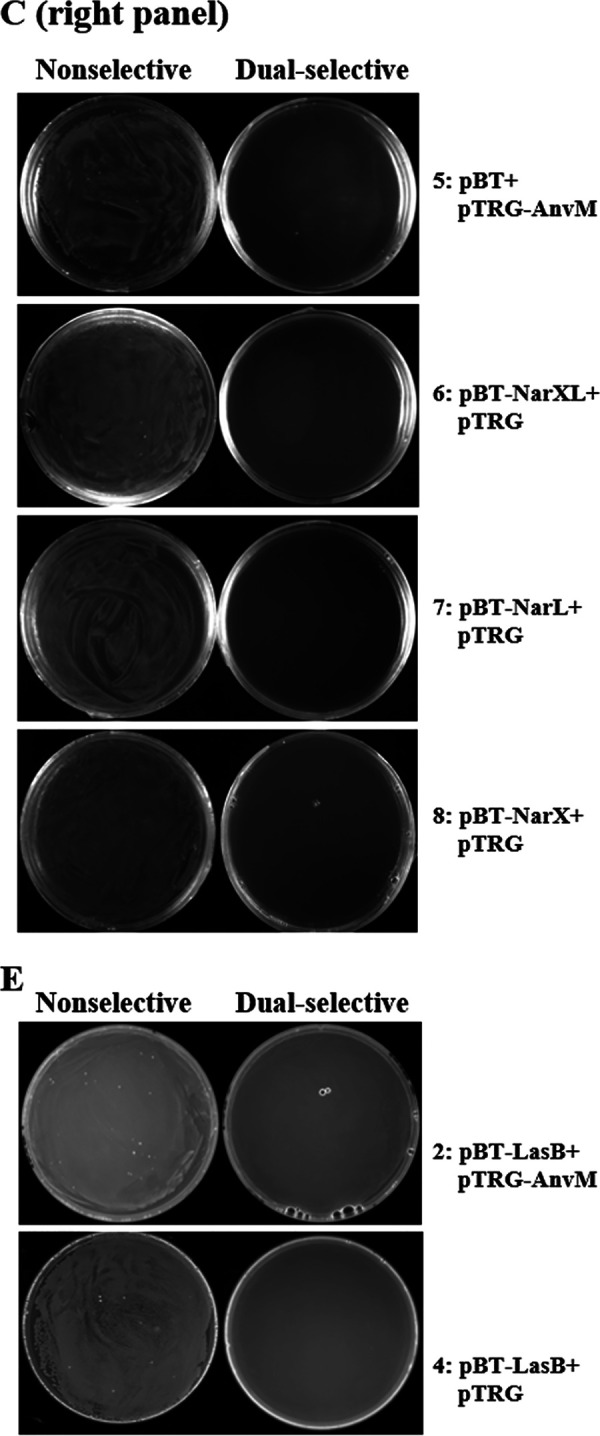# Correction for Zhang et al., “Pseudomonas aeruginosa Regulatory Protein AnvM Controls Pathogenicity in Anaerobic Environments and Impacts Host Defense”

**DOI:** 10.1128/mBio.02368-20

**Published:** 2020-10-13

**Authors:** Yingchao Zhang, Chuan-min Zhou, Qinqin Pu, Qun Wu, Shirui Tan, Xiaolong Shao, Weitong Zhang, Yingpeng Xie, Rongpeng Li, Xue-jie Yu, Rui Wang, Liang Zhang, Min Wu, Xin Deng

**Affiliations:** a Department of Biomedical Sciences, City University of Hong Kong, Hong Kong, People’s Republic of China; b Department of Biomedical Sciences, University of North Dakota, Grand Forks, North Dakota, USA; c State Key Laboratory of Virology, School of Health Sciences, Wuhan University, Wuhan, People’s Republic of China; d Key Laboratory of Biotechnology for Medicinal Plants of Jiangsu Province, Jiangsu Normal University, Xuzhou, Jiangsu, People’s Republic of China

## AUTHOR CORRECTION

Volume 10, no. 4, e01362-19, https://doi.org/10.1128/mBio.01362-19. In Fig. 2I, Fig. 5A and B, and Fig. S2C and E, the following incorrect images were inadvertent duplicates of each other. Figure 2I-1 and -3 and Fig. S2E-1 and -3 were unnecessary duplicates of Fig. 2A-1 and -3. Figure 5B-1 and -3 were unnecessary duplicates of Fig. 5A-1 and -3. Figure 5A-4 and B-4 were duplicates of Fig. 2A-3. Figure S2C-6 (left) and S2E-4 (left) and S2E-4 (right) were duplicates of Fig. S2C-1 (right), 2I-4 (down), and S2D-4 (right), respectively. The correct figures are shown below, which do not change results or conclusions in the paper. We apologize for any inconvenience to the readers.

**FIG 2I:**
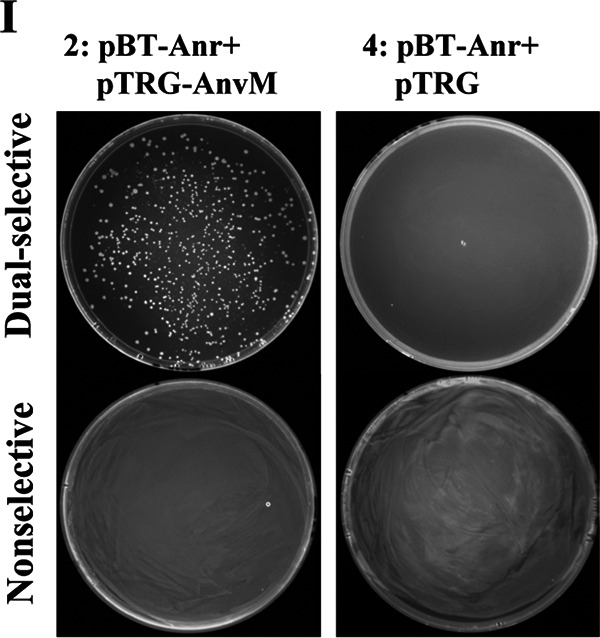


**FIG 5A/B:**
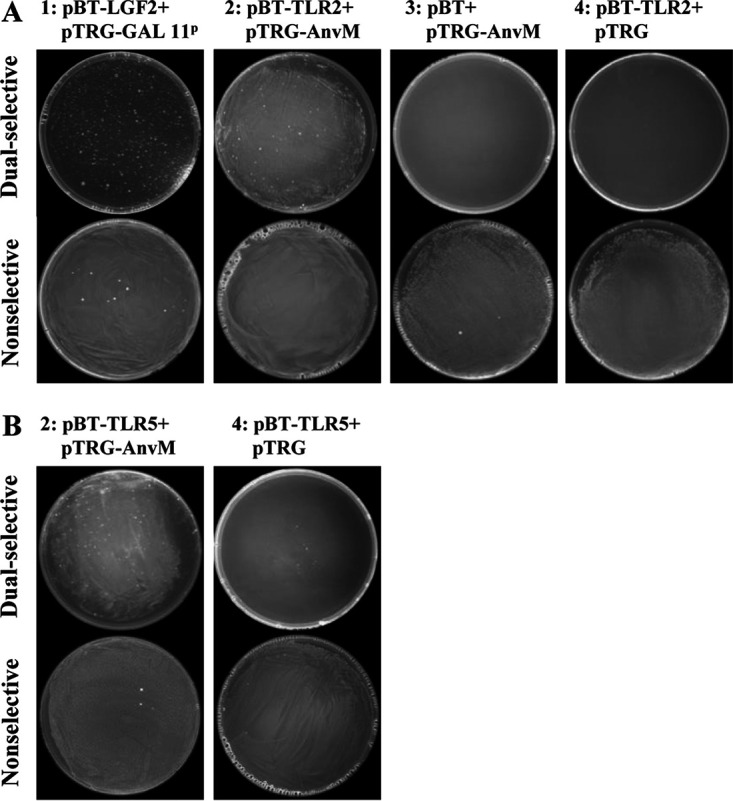


**FIG S2C/E:**