# Supplementary material for: Pseudomonas aeruginosa Regulatory Protein AnvM Controls Pathogenicity in Anaerobic Environments and Impacts Host Defense
Source: mBio. 2019 Jul 23;10(4):e01362-19. doi: 10.1128/mBio.01362-19 (PMC6650552; doi:10.1128/mBio.01362-19)
Supplement: TABLE S1 [file mBio.01362-19-st001.docx]

**Table S1A. Bacterial strains and plasmids used in this study.**

| **Strain or plasmid** | **Relevant characteristics** | **Source** |
| --- | --- | --- |
| ***E. coli*** |  |  |
| DH5α | *F^–^ φ80lacZ ΔM15 Δ(lacZYA-argF)U169 recA1 endA1 hsdR17(rk^–^, mk^+^)phoA supE44 thi-1 gyrA96 relA1 tonA* | Stratagene |
| BL21 star (DE3) | F^-^ *ompT hsdS*_B_ (rB^-^ m_B_^-^) *gal dcm met* (DE3) | Invitrogen |
| ***P. aeruginosa*** |  |  |
| PAO1 | Wild type | This lab |
| MPAO1 | Wild type | This lab |
| *anvM* deletion | clean deletion of anvM | This study |
| *anvM* deletion/complemented | deletion/complemented for control of polar effect | This study |
| **Plasmid** |  |  |
| pAK1900 | *E. coli–P. aeruginosa* shuttle cloning vector, Ap^r^ Cb^r^ | 1 |
| pEX18Ap | Gene replacement vector, *mob+sacB*, Ap | 2 |
| pPS858 | pBR322 derivative carrying a FRT-Gm cassette, Ap | 2 |
| pBT | p15A origin of replication, lac-UV5 promoter, λ cI open reading fram; Cmr | Agilent |
| pTRG | ColE1 origin of replication, lpp promoter, lac-UV5 promoter, RNAPα open reading frame; Tcr | Agilent |
| pBT-LGF2 | Interaction control plasmid encoding the dimerization domain (40 amino acid residues) of the Gal4 transcriptional activator protein; Cmr | Agilent |
| pTRG-GAII 1p | Interaction control plasmid encoding a domain (90 amino acid residues) of the mutant form of the GaII 1 protein; Tcr | Agilent |
| pET28a | T7 *lac* promoter-operator, N-terminal His tag, Kan^r^ | Novagen |
| pAK1900-*anvM* | pAK1900 derivative carrying *anvM* on a HindIII/BamHI fragment in same orientation as plac | This study |
| pAK1900-*anvM*-C44S | pAK1900-*anvM* derivative carrying serine substitution mutant at the site C44 | This study |
| pAK1900-*anvM*-Flag | Expression plasmid, pAK1900-*anvM*-Flag containing the entire *anvM* gene and the 3x flag sequence | This study |
| pEX18Ap-*anvM* | pEX18Ap derivative, for replacing MPAO1 *anvM* locus without a gentamicin resistance cassette | This study |
| pEX18Ap-*anvM*-Gm | pEX18Ap derivative, for replacing MPAO1 *anvM* locus with a gentamicin resistance cassette | This study |
| pTRG-*anvM* | pTRG plasmid containing the entire *anvM* gene | This study |
| pBT-*mvfR* | pBT plasmid containing the entire *mvfR* gene | This study |
| pBT-*anr* | pBT plasmid containing the entire *anr* gene | This study |
| pBT-*TLR2* | pBT plasmid containing the entire *TLR2* gene | This study |
| pBT-*TLR5* | pBT plasmid containing the entire *TLR5* gene | This study |
| pBT-*narL* | pBT plasmid containing the entire *narL* gene | This study |
| pBT-*narX* | pBT plasmid containing the entire *narX* gene | This study |
| pBT-*narXL* | pBT plasmid containing the entire *narX and narL* genes | This study |
| pBT-*lasR* | pBT plasmid containing the entire *lasR* gene | This study |
| pBT-*lasB* | pBT plasmid containing the entire *lasB* gene | This study |
| pET28a-*anr* | Protein expression construct, the entire gene of Anr cloned in pET28a vector | This study |
| pET28a-*mvfRC87* | pET28a-*mvfRC87* lacks the 87 N-terminal residues of the full-length protein, which include a predicted DNA-binding motif. | This study |

1. Poole, K., Neshat, S., Krebes, K. & Heinrichs, D.E. Cloning and nucleotide sequence analysis of the ferripyoverdine receptor gene fpvA of *Pseudomonas aeruginosa*. *J Bacteriol* 175, 4597-604 (1993).
2. Hoang, T.T., Karkhoff-Schweizer, R.R., Kutchma, A.J. & Schweizer, H.P. A broad-host-range Flp-FRT recombination system for site-specific excision of chromosomally-located DNA sequences: application for isolation of unmarked *Pseudomonas aeruginosa* mutants. *Gene* 212, 77-86 (1998).

**Table S1B. Primers used in this study.**

| **Primer** | **Sequence** |
| --- | --- |
| pEX-*anvM*-up-S | CTCGAATTCCGAGCGCTTCAACCAGAT |
| pEX-*anvM*-up-A | TCCTCTAGAGAAGGAAGCCGCCAGTATC |
| pEX-*anvM*-down-S | TCCTCTAGACAGCAGTGCGCTAGGATGT |
| pEX-*anvM*-down-A | TCGAAGCTTCCTTGGAGGCGCAGTAGG |
| pAK-*anvM*-F | CCCAAGCTTGATTCCGGTCAAGAAATCCA |
| pAK-*anvM*-R | CGGGATCCGGTACGGGCACGCTTACG |
| *anvM*-C44S-F | GCGGAGATGTCCAGCATCGCCGGAG |
| *anvM*-C44S-R | CTCCGGCGATGCTGGACATCTCCGC |
| PAK1900-*anvM*-Flag-F | ACACTATAGAATACTCAAGCTTATGATGTTCGAACGCAGCC |
| PAK1900-*anvM*-Flag-R | GAGCTCGGTACCCGGGGATCCTTACTTATCGTCGTCATCCTTGTAATCTTCGCAGCGGCGCTT |
| *TLR2*-NotI-F | TTGCGGCCGCAATGCCACATACTTTGTG |
| *TLR2*-BamHI-R | TTGGATCCCTAGGACTTTATCGCAG |
| *TLR5*-NotI-F | TTGCGGCCGCAATGGGAGACCACCTGGACCTT |
| *TLR5*-BamHI-R | TTGGATCCTTAGGAGATGGTTGC |
| *hsiA2*-RT-F | CCCATCTCCAGAACGACAGT |
| *hsiA2*-RT-R | GCTCAGTTGCCAGAAGAACC |
| *hcnA*-RT-F | GCAGACATGACCATCCACCT |
| *hcnA*-RT-R | CGGTTGCTTTCGGTTTCCA |
| *lptD*-RT-F | AGCACTACGTACGTCAACCA |
| *lptD*-RT-R | AACTCGGTGCCATAGGTGAA |
| *dnpA*-RT-F | CTGGGTTATTTCTGCCTGCA |
| *dnpA*-RT-R | AGCAGATTGTTCCAGGTCGA |
| *fliF*-RT-F | TACAGGATTCGGTGGGCTAC |
| *fliF*-RT-R | TTGACGATGTCCCAGAACCA |
| *fliP*-RT-F | AGGAGTATTCGGTCAGCCTG |
| *fliP*-RT-R | GAACATGGTCAGGAACAGCG |
| *pqsA*-RT-F | ATCCCGATACCGCCGTTTAT |
| *pqsA*-RT-R | GATTGATCACGGCGGGAATG |
| *katA*-RT-F: | GCATCGAGAACCTGACCAAC |
| *katA*-RT-R: | CTGCACGTACATCTTCCAGC |
| *hemN*-RT-F | AGGACTTCGACATGGAGGTG |
| *hemN*-RT-R: | GTCCGTAGATCAGGTCGAGG |
| 16SRNA-RT-F | TCTTCGGACCTCACGCTATC |
| 16SRNA-RT-R | CAATATTCCCCACTGCTGCC |
| *mvfR*-EcoRI-F | TCGAATTCCATGCCTATTCATAACCTG |
| *mvfR*-XhoI-R | TTCTCGAGCTACTCTGGTGCGGC |
| *lasB*-EcoRI-F | CGGAATTCATGAAGAAGGTTTCTACGC |
| *lasB*-XhoI-R | TTCTCGAGTTACAACGCGCTC |
| *anr*-NotI-F | TTGCGGCCGCAATGGCCGAAACCATCAAGG |
| *anr*-XhoI-R | TTCTCGAGTCAGCCTTCCAGCTG |
| pET28a-*mvfR*C87-F | AGCAAATGGGTCGCGGATCCATGCTCAACAAGGGTC |
| pET28a-*mvfR*C87-R | CGGAGCTCGAATTCGGATCCCTACTCTGGTGCG |
| pET28a-*anr*-F | TTGGATCCATGGCCGAAACCATCAAG |
| pET28a-*anr*-R | TACTCGAGTCAGCCTTCCAGCTG |
| *lasR*-EcoRI-F | TTGAATTCCATGGCCTTGGTTGACGG |
| *lasR*-BamHI-R | ATGGATCCTCAGAGAGTAATAAGACC |

**Table S1C. Differential expression of 693 genes in *Pseudomonas aeruginosa* PAO1 under the aerobic condition.**

| **Gene** | **Δ*anvM* VS WT log2(fold_change)** | **Function** |  |  |
| --- | --- | --- | --- | --- |
| *cobM* | 3.62403 | precorrin-3 methylase | | |
| PA0488 | 3.36086 | conserved hypothetical protein | | |
| *ligD* | 3.12056 | Multifunctional non-homologous end joining protein LigD | | |
| PA1379 | 3.01478 | probable short-chain dehydrogenase | | |
| *hcnA* | 2.86391 | hydrogen cyanide synthase HcnA | | |
| PA2855 | 2.76891 | hypothetical protein | |  |
| PA5059 | 2.76096 | probable transcriptional regulator | | |
| PA2324 | 2.7597 | hypothetical protein | |  |
| PA4593 | 2.75313 | probable permease of ABC transporter | | |
| PA1034 | 2.71278 | hypothetical protein | |  |
| *pvdQ* | 2.67237 | 3-oxo-C12-homoserine lactone acylase PvdQ | | |
| PA0237 | 2.65549 | probable oxidoreductase | | |
| PA0570 | 2.65406 | hypothetical protein | |  |
| PA1289 | 2.63804 | hypothetical protein | |  |
| PA3130 | 2.62641 | hypothetical protein | |  |
| PA2377 | 2.62074 | hypothetical protein | |  |
| PA3489 | 2.61517 | conserved hypothetical protein | | |
| PA2216 | 2.59381 | 2-keto-3-deoxy-D-arabinonate dehydratase | | |
| *lhpE* | 2.57133 | D-hydroxyproline dehydrogenase alpha-subunit, LphE | | |
| PA2029 | 2.50127 | hypothetical protein | |  |
| PA5159 | 2.45231 | multidrug resistance protein | | |
| PA1451 | 2.42217 | conserved hypothetical protein | | |
| PA4821 | 2.41459 | probable transporter | |  |
| PA4871 | 2.41136 | hypothetical protein | |  |
| PA4994 | 2.33984 | probable acyl-CoA dehydrogenase | | |
| PA5342 | 2.33136 | probable transcriptional regulator | | |
| *hasD* | 2.31681 | transport protein HasD | | |
| PA0648 | 2.29508 | hypothetical protein | |  |
| *erbR* | 2.28119 | response regulator ErbR | | |
| PA0781 | 2.27501 | hypothetical protein | |  |
| PA3320 | 2.27153 | hypothetical protein | |  |
| *pscL* | 2.27122 | type III export protein PscL | | |
| PA0701 | 2.26403 | probable transcriptional regulator | | |
| *lhpA* | 2.24935 | Hydroxyproline 2-epimerase, LhpA | | |
| PA0823 | 2.2485 | hypothetical protein | |  |
| PA1237 | 2.24367 | probable multidrug resistance efflux pump | | |
| PA5543 | 2.23353 | hypothetical protein | |  |
| PA2559a | 2.22247 |  |  |  |
| PA2181 | 2.21245 | hypothetical protein | |  |
| *hutR* | 2.2045 | HutR |  |  |
| PA1894 | 2.19781 | hypothetical protein | |  |
| PA2166 | 2.17814 | hypothetical protein | |  |
| *sphC* | 2.16554 | SphC |  |  |
| PA2723 | 2.15034 | hypothetical protein | |  |
| PA2922 | 2.14523 | probable hydrolase | |  |
| PA1916 | 2.14095 | probable amino acid permease | | |
| PA4145a | 2.13928 |  |  |  |
| *gltS* | 2.12048 | glutamate/sodium ion symporter, GltS | | |
| *bioD* | 2.11867 | dethiobiotin synthase | | |
| PA4834 | 2.11112 | putative nicotianamine synthase | | |
| PA1063 | 2.10404 | hypothetical protein | |  |
| *dguC* | 2.09854 | DguC |  |  |
| PA2102 | 2.08972 | hypothetical protein | |  |
| PA5457 | 2.07835 | methyltransferase | |  |
| *eraS* | 2.03445 | sensor kinase, EraS | |  |
| *vanA* | 2.0342 | vanillate O-demethylase oxygenase subunit | | |
| PA5311 | 2.00459 | probable major facilitator superfamily (MFS) transporter | | |
| PA4177 | 1.99914 | hypothetical protein | |  |
| PA1490 | 1.98808 | probable transcriptional regulator | | |
| *hptB* | 1.96632 | Histidine phosphotransfer protein HptB | | |
| PA3132 | 1.94656 | probable hydrolase | |  |
| PA1238 | 1.94188 | probable outer membrane component of multidrug efflux pump | | |
| PA2829 | 1.91495 | hypothetical protein | |  |
| PA3897 | 1.91223 | hypothetical protein | |  |
| *xylS* | 1.9098 | transcriptional regulator XylS | | |
| *tsi2* | 1.90678 | Tsi2 |  |  |
| *pscF* | 1.90443 | type III export protein PscF | | |
| PA0136 | 1.9008 | probable ATP-binding component of ABC transporter | | |
| PA2271 | 1.89778 | probable acetyltransferase | | |
| PA0014 | 1.88907 | hypothetical protein | |  |
| PA0184 | 1.88843 | probable ATP-binding component of ABC transporter | | |
| PA0236 | 1.8855 | probable transcriptional regulator | | |
| *dksA2* | 1.88372 | DksA2 |  |  |
| PA0881 | 1.88214 | hypothetical protein | |  |
| PA2881 | 1.88202 | probable two-component response regulator | | |
| PA3749 | 1.87089 | probable major facilitator superfamily (MFS) transporter | | |
| *purU2* | 1.87057 | formyltetrahydrofolate deformylase | | |
| *algK* | 1.86537 | alginate biosynthetic protein AlgK precursor | | |
| *mep72* | 1.86094 | Mep72 |  |  |
| PA0739 | 1.85414 | probable transcriptional regulator | | |
| PA3284 | 1.85117 | hypothetical protein | |  |
| PA3513 | 1.83474 | hypothetical protein | |  |
| *pheC* | 1.83248 | cyclohexadienyl dehydratase precursor | | |
| PA0829 | 1.82891 | probable hydrolase | |  |
| PA2196 | 1.82274 | TetR family transcriptional regulator | | |
| *tpm* | 1.82122 | thiopurine methyltransferase | | |
| PA3994 | 1.82004 | probable epoxide hydrolase | | |
| PA4574 | 1.80449 | conserved hypothetical protein | | |
| PA0565 | 1.78655 | conserved hypothetical protein | | |
| PA2472 | 1.78101 | probable major facilitator superfamily (MFS) transporter | | |
| PA2225 | 1.77687 | hypothetical protein | |  |
| PA1907 | 1.75905 | hypothetical protein | |  |
| PA2839 | 1.74822 | conserved hypothetical protein | | |
| PA0439 | 1.74701 | probable oxidoreductase | | |
| PA3505 | 1.74695 | hypothetical protein | |  |
| PA4644 | 1.74574 | hypothetical protein | |  |
| PA1508 | 1.73365 | hypothetical protein | |  |
| *hxcP* | 1.7241 | HxcP |  |  |
| PA5157 | 1.71798 | probable transcriptional regulator | | |
| PA0751 | 1.70932 | conserved hypothetical protein | | |
| PA3741 | 1.69874 | hypothetical protein | |  |
| PA0241 | 1.69199 | probable major facilitator superfamily (MFS) transporter | | |
| PA3717 | 1.67966 | probable peptidyl-prolyl cis-trans isomerase, FkbP-type | | |
| PA2590 | 1.67608 | hypothetical protein | |  |
| PA2916 | 1.67301 | hypothetical protein | |  |
| *ccoO2* | 1.67226 | Cytochrome c oxidase, cbb3-type, CcoO subunit | | |
| PA2339 | 1.66187 | probable binding-protein-dependent maltose/mannitol transport protein | | |
| *mreD* | 1.66065 | rod shape-determining protein MreD | | |
| PA1286 | 1.65864 | probable major facilitator superfamily (MFS) transporter | | |
| PA3089a | 1.64672 |  |  |  |
| PA3039 | 1.64353 | probable transporter | |  |
| *copS* | 1.64205 | two-component sensor, CopS | | |
| PA1558 | 1.63954 | hypothetical protein | |  |
| PA2285 | 1.62746 | hypothetical protein | |  |
| *algX* | 1.62231 | alginate biosynthesis protein AlgX | | |
| PA1352 | 1.61736 | conserved hypothetical protein | | |
| *rrmA* | 1.60889 | rRNA methyltransferase | | |
| PA1914 | 1.60225 | conserved hypothetical protein | | |
| PA4062 | 1.60204 | hypothetical protein | |  |
| PA0631 | 1.59952 | hypothetical protein | |  |
| PA3906 | 1.59465 | hypothetical protein | |  |
| PA2822 | 1.59418 | conserved hypothetical protein | | |
| *ccoQ2* | 1.57746 | Cytochrome c oxidase, cbb3-type, CcoQ subunit | | |
| PA1228 | 1.57398 | hypothetical protein | |  |
| PA2292 | 1.56493 | hypothetical protein | |  |
| *ccoN2* | 1.55475 | Cytochrome c oxidase, cbb3-type, CcoN subunit | | |
| PA3938 | 1.55305 | probable periplasmic taurine-binding protein precursor | | |
| *pcaH* | 1.55128 | protocatechuate 3,4-dioxygenase, beta subunit | | |
| PA3253 | 1.55045 | probable permease of ABC transporter | | |
| PA0532 | 1.5504 | hypothetical protein | |  |
| PA2655 | 1.54757 | hypothetical protein | |  |
| *arnB* | 1.54211 | ArnB |  |  |
| *vreI* | 1.54203 | ECF sigma factor, VreI | | |
| PA3462 | 1.5413 | probable sensor/response regulator hybrid | | |
| *femA* | 1.53616 | ferric-mycobactin receptor, FemA | | |
| PA1424a | 1.53082 |  |  |  |
| PA0054 | 1.53014 | conserved hypothetical protein | | |
| PA1603 | 1.52894 | probable transcriptional regulator | | |
| PA2105 | 1.52408 | probable acetyltransferase | | |
| *tssL1* | 1.51477 | TssL1 |  |  |
| PA5397 | 1.51295 | hypothetical protein | |  |
| PA1213 | 1.51208 | hypothetical protein | |  |
| PA4077 | 1.51092 | probable transcriptional regulator | | |
| PA2676 | 1.50803 | probable type II secretion system protein | | |
| PA3512 | 1.50011 | probable permease of ABC transporter | | |
| *wspD* | 1.49641 | hypothetical protein | |  |
| PA4818 | 1.49381 | conserved hypothetical protein | | |
| PA1232 | 1.48923 | hypothetical protein | |  |
| *pscK* | 1.48757 | type III export protein PscK | | |
| *xqhA* | 1.48266 | secretion protein XqhA | | |
| PA3493 | 1.47844 | conserved hypothetical protein | | |
| *lhpB* | 1.47832 | D-hydroxyproline dehydrogenase beta-subunit, LphB | | |
| PA2171 | 1.47816 | hypothetical protein | |  |
| *tadC* | 1.47637 | TadC |  |  |
| PA1923 | 1.46893 | hypothetical protein | |  |
| *soxR* | 1.46672 | SoxR |  |  |
| *hisM* | 1.46663 | histidine transport system permease HisM | | |
| PA0644 | 1.46576 | hypothetical protein | |  |
| *pcaD* | 1.46472 | beta-ketoadipate enol-lactone hydrolase | | |
| PA1918 | 1.46426 | hypothetical protein | |  |
| PA2715 | 1.46264 | probable ferredoxin | |  |
| PA1146 | 1.46051 | probable iron-containing alcohol dehydrogenase | | |
| PA0227 | 1.45734 | probable CoA transferase, subunit B | | |
| *algI* | 1.45725 | alginate o-acetyltransferase AlgI | | |
| *xcpW* | 1.45135 | general secretion pathway protein J | | |
| *tag* | 1.44986 | DNA-3-methyladenine glycosidase I | | |
| PA3869 | 1.44446 | hypothetical protein | |  |
| PA0193 | 1.44132 | hypothetical protein | |  |
| *cbiD* | 1.43774 | cobalamin biosynthetic protein CbiD | | |
| PA0277 | 1.43667 | conserved hypothetical protein | | |
| *vreR* | 1.43254 | sigma factor regulator, VreR | | |
| *nagE* | 1.43135 | N-Acetyl-D-Glucosamine phosphotransferase system transporter | | |
| PA2118a | 1.43092 |  |  |  |
| PA3342 | 1.42894 | hypothetical protein | |  |
| *algE* | 1.42447 | Alginate production outer membrane protein AlgE precursor | | |
| PA5402 | 1.4205 | hypothetical protein | |  |
| PA2077 | 1.41962 | oleate 10S-lipoxygenase | | |
| *hasS* | 1.41796 | HasS |  |  |
| PA1963 | 1.41634 | hypothetical protein | |  |
| *pvcD* | 1.41223 | paerucumarin biosynthesis protein PvcD | | |
| *sphD* | 1.41077 | SphD |  |  |
| *hsiF2* | 1.40742 | HsiF2 |  |  |
| PA1020 | 1.40054 | probable acyl-CoA dehydrogenase | | |
| *pvcB* | 1.39741 | paerucumarin biosynthesis protein PvcB | | |
| PA2600 | 1.3974 | hypothetical protein | |  |
| PA5392 | 1.38934 | conserved hypothetical protein | | |
| PA1965 | 1.38799 | hypothetical protein | |  |
| PA4424 | 1.3845 | conserved hypothetical protein | | |
| *pmrB* | 1.38351 | PmrB: two-component regulator system signal sensor kinase PmrB | | |
| *atuF* | 1.38083 | geranyl-CoA carboxylase, alpha-subunit (biotin-containing) | | |
| PA4121 | 1.37953 | conserved hypothetical protein | | |
| PA0828 | 1.37844 | probable transcriptional regulator | | |
| PA1166 | 1.37821 | hypothetical protein | |  |
| PA3224 | 1.37228 | hypothetical protein | |  |
| PA2299 | 1.36974 | probable transcriptional regulator | | |
| PA2335 | 1.36838 | probable TonB-dependent receptor | | |
| PA4126 | 1.36632 | probable major facilitator superfamily (MFS) transporter | | |
| PA0475 | 1.36298 | probable transcriptional regulator | | |
| PA1153 | 1.35998 | hypothetical protein | |  |
| PA0850 | 1.35815 | hypothetical protein | |  |
| PA2350 | 1.35707 | probable ATP-binding component of ABC transporter | | |
| PA1428a | 1.35408 |  |  |  |
| PA1217 | 1.35257 | probable 2-isopropylmalate synthase | | |
| PA2074 | 1.35044 | hypothetical protein | |  |
| *ligT* | *1.34493* | *2'-5' RNA ligase* | |  |
| PA1111 | 1.34066 | hypothetical protein | |  |
| PA1226 | 1.32592 | probable transcriptional regulator | | |
| PA3051 | 1.29126 | hypothetical protein | |  |
| PA1922 | 1.29113 | probable TonB-dependent receptor | | |
| PA1881 | 1.28558 | probable oxidoreductase | | |
| PA5031 | 1.28463 | probable short chain dehydrogenase | | |
| PA2439 | 1.28232 | hypothetical protein | |  |
| PA4894 | 1.28024 | hypothetical protein | |  |
| PA2125 | 1.2696 | probable aldehyde dehydrogenase | | |
| PA2325 | 1.26803 | hypothetical protein | |  |
| *ccoP2* | 1.26783 | Cytochrome c oxidase, cbb3-type, CcoP subunit | | |
| PA3681 | 1.26766 | hypothetical protein | |  |
| PA1391 | 1.26607 | probable glycosyl transferase | | |
| PA3072 | 1.26575 | hypothetical protein | |  |
| *exaC* | 1.26572 | NAD+ dependent aldehyde dehydrogenase ExaC | | |
| PA1387 | 1.25414 | hypothetical protein | |  |
| PA2636 | 1.2528 | hypothetical protein | |  |
| *tadD* | 1.25107 | TadD |  |  |
| PA2393 | 1.24534 | putative dipeptidase | |  |
| PA0203 | 1.24501 | probable binding protein component of ABC transporter | | |
| PA1214 | 1.24445 | hypothetical protein | |  |
| *gmk* | 1.24409 | guanylate kinase | |  |
| PA2568 | 1.24337 | hypothetical protein | |  |
| *pauA3* | 1.24196 | Glutamylpolyamine synthetase | | |
| *pqqD* | 1.23704 | pyrroloquinoline quinone biosynthesis protein D | | |
| *gcl* | 1.23638 | glyoxylate carboligase | | |
| PA3073 | 1.23634 | hypothetical protein | |  |
| *exsE* | 1.23443 | ExsE |  |  |
| PA5033 | 1.22424 | hypothetical protein | |  |
| PA0814 | 1.2213 | conserved hypothetical protein | | |
| *pscD* | 1.22104 | type III export protein PscD | | |
| PA3330 | 1.21892 | probable short chain dehydrogenase | | |
| PA5079 | 1.21386 | conserved hypothetical protein | | |
| PA1343 | 1.2113 | hypothetical protein | |  |
| PA1763 | 1.21059 | hypothetical protein | |  |
| PA4840 | 1.21021 | conserved hypothetical protein | | |
| PA2067 | 1.20927 | probable hydrolase | |  |
| PA1125 | 1.20786 | probable cobalamin biosynthetic protein | | |
| PA1425 | 1.20643 | probable ATP-binding component of ABC transporter | | |
| *nalC* | 1.20601 | NalC |  |  |
| *pslN* | 1.20254 | hypothetical protein | |  |
| PA2287 | 1.20107 | hypothetical protein | |  |
| PA2163 | 1.19924 | hypothetical protein | |  |
| *mexK* | 1.19777 | MexK |  |  |
| PA0117 | 1.19668 | probable short chain dehydrogenase | | |
| PA2164 | 1.18983 | probable glycosyl hydrolase | | |
| PA1621 | 1.18505 | probable hydrolase | |  |
| *pelA* | 1.18271 | PelA |  |  |
| PA4396 | 1.1787 | two-component response regulator | | |
| PA0446 | 1.17316 | conserved hypothetical protein | | |
| *mgtA* | 1.17277 | Mg(2+) transport ATPase, P-type 2 | | |
| PA2769 | 1.17123 | hypothetical protein | |  |
| PA0813 | 1.16677 | hypothetical protein | |  |
| PA3954 | 1.16673 | hypothetical protein | |  |
| PA2298 | 1.16538 | probable oxidoreductase | | |
| PA3965 | 1.16526 | probable transcriptional regulator | | |
| PA0989 | 1.16476 | hypothetical protein | |  |
| PA3825 | 1.16256 | Cyclic-guanylate-specific phosphodiesterase | | |
| PA4948 | 1.14981 | conserved hypothetical protein | | |
| PA0791 | 1.14597 | probable transcriptional regulator | | |
| *mexG* | 1.14369 | hypothetical protein | |  |
| *nfxB* | 1.14345 | transcriptional regulator NfxB | | |
| PA0760 | 1.143 | conserved hypothetical protein | | |
| PA0531 | 1.13976 | probable glutamine amidotransferase | | |
| *atuC* | 1.13918 | geranyl-CoA carboxylase, beta-subunit | | |
| PA3415 | 1.13469 | probable dihydrolipoamide acetyltransferase | | |
| PA4466 | 1.13231 | probable phosphoryl carrier protein | | |
| PA0028 | 1.13162 | hypothetical protein | |  |
| *carS* | 1.13027 | two-component sensor CarS | | |
| PA0757 | 1.12788 | probable two-component sensor | | |
| PA5030 | 1.1275 | probable major facilitator superfamily (MFS) transporter | | |
| PA1038 | 1.1271 | hypothetical protein | |  |
| *tse4* | 1.12557 | Tse4 |  |  |
| PA5314 | 1.12205 | hypothetical protein | |  |
| PA0941 | 1.11925 | hypothetical protein | |  |
| PA1652 | 1.1183 | hypothetical protein | |  |
| *rimJ* | 1.11329 | ribosomal protein alanine acetyltransferase | | |
| *dinB* | 1.11299 | DNA Polymerase IV, DinB | | |
| PA0322 | 1.11191 | probable transporter | |  |
| *opdH* | 1.11147 | cis-aconitate porin OpdH | | |
| PA1731 | 1.11068 | conserved hypothetical protein | | |
| PA1879 | 1.11052 | hypothetical protein | |  |
| PA0068 | 1.10628 | hypothetical protein | |  |
| PA1825 | 1.10594 | hypothetical protein | |  |
| PA3511 | 1.10291 | probable short-chain dehydrogenase | | |
| *plcN* | 1.10283 | non-hemolytic phospholipase C precursor | | |
| PA0223 | 1.10219 | probable dihydrodipicolinate synthetase | | |
| *sfa3* | 1.09849 | probable transcriptional regulator | | |
| PA1363 | 1.09826 | ECF sigma factor | |  |
| PA3679 | 1.0979 | hypothetical protein | |  |
| PA4317 | 1.09067 | hypothetical protein | |  |
| PA4886 | 1.0894 | probable two-component sensor | | |
| PA2455 | 1.08881 | hypothetical protein | |  |
| PA4004 | 1.08332 | conserved hypothetical protein | | |
| PA3317 | 1.08319 | hypothetical protein | |  |
| PA4636a | 1.08074 |  |  |  |
| PA0550 | 1.07936 | conserved hypothetical protein | | |
| PA3422 | 1.07334 | hypothetical protein | |  |
| *arnC* | 1.07228 | ArnC |  |  |
| PA2980 | 1.06928 | conserved hypothetical protein | | |
| PA0567 | 1.06907 | conserved hypothetical protein | | |
| PA2230 | 1.06535 | hypothetical protein | |  |
| PA0182 | 1.06393 | probable short-chain dehydrogenase | | |
| PA1331 | 1.06176 | conserved hypothetical protein | | |
| PA1044 | 1.06103 | hypothetical protein | |  |
| PA1067 | 1.05899 | probable transcriptional regulator | | |
| PA1189 | 1.05865 | conserved hypothetical protein | | |
| PA2198 | 1.05853 | hypothetical protein | |  |
| PA2599 | 1.05732 | conserved hypothetical protein | | |
| PA1235 | 1.0543 | probable transcriptional regulator | | |
| PA0884 | 1.05375 | probable C4-dicarboxylate-binding periplasmic protein | | |
| PA0160 | 1.05174 | hypothetical protein | |  |
| *hisH1* | 1.05119 | glutamine amidotransferase | | |
| PA3019 | 1.04632 | probable ATP-binding component of ABC transporter | | |
| PA2914 | 1.04615 | probable permease of ABC transporter | | |
| PA1993 | 1.04519 | probable major facilitator superfamily (MFS) transporter | | |
| PA2172 | 1.04351 | hypothetical protein | |  |
| PA4537 | 1.04348 | hypothetical protein | |  |
| PA5481 | 1.04263 | hypothetical protein | |  |
| *ccmA* | 1.04223 | heme exporter protein CcmA | | |
| PA1995 | 1.04164 | hypothetical protein | |  |
| PA2768 | 1.0412 | hypothetical protein | |  |
| PA3021 | 1.03962 | hypothetical protein | |  |
| PA2712 | 1.03676 | hypothetical protein | |  |
| PA2045 | 1.03569 | conserved hypothetical protein | | |
| PA0267 | 1.02759 | hypothetical protein | |  |
| *fepD* | 1.02758 | ferric enterobactin transport protein FepD | | |
| PA3782 | 1.02746 | probable transcriptional regulator | | |
| *trxB2* | 1.02608 | thioredoxin reductase 2 | | |
| *ureD* | 1.02266 | urease accessory protein | | |
| PA2351 | 1.02185 | probable permease of ABC transporter | | |
| PA1926 | 1.02183 | Uncharacterized protein | | |
| *thrH* | 1.02161 | homoserine kinase | |  |
| *napC* | 1.02076 | cytochrome c-type protein NapC | | |
| PA0098 | 1.01969 | hypothetical protein | |  |
| PA1638 | 1.01638 | conserved hypothetical protein | | |
| PA1692 | 1.01622 | probable translocation protein in type III secretion | | |
| *cobN* | 1.01422 | cobalamin biosynthetic protein CobN | | |
| *opmQ* | 1.01319 | probable outer membrane protein precursor | | |
| PA2720 | 1.01301 | hypothetical protein | |  |
| PA4353 | 1.01271 | conserved hypothetical protein | | |
| *fecI* | 1.00975 | FecI |  |  |
| PA1676 | 1.00696 | hypothetical protein | |  |
| PA3474 | 1.00391 | conserved hypothetical protein | | |
| PA2540 | 1.00166 | conserved hypothetical protein | | |
| PA2209 | 1.00138 | hypothetical protein | |  |
| PA0240 | 1.00075 | probable porin | |  |
| PA3368 | -1.00041 | probable acetyltransferase | | |
| PA2204 | -1.00115 | probable binding protein component of ABC transporter | | |
| PA2553 | -1.00191 | probable acyl-CoA thiolase | | |
| PA3882 | -1.00489 | hypothetical protein | |  |
| PA3413 | -1.01307 | conserved hypothetical protein | | |
| PA0737 | -1.01867 | hypothetical protein | |  |
| PA0166 | -1.019 | probable transporter | |  |
| *pobA* | -1.01964 | p-hydroxybenzoate hydroxylase | | |
| PA3365 | -1.02082 | probable chaperone | |  |
| *liuR* | -1.02497 | regulator of liu genes | |  |
| *mexF* | -1.03204 | Resistance-Nodulation-Cell Division (RND) multidrug efflux transporter MexF | | |
| PA5432 | -1.03771 | probable acetyltransferase | | |
| PA3239 | -1.03918 | conserved hypothetical protein | | |
| PA5514 | -1.04666 | probable beta-lactamase | | |
| PA2693 | -1.04753 | conserved hypothetical protein | | |
| PA3720 | -1.04878 | hypothetical protein | |  |
| *dht* | -1.0489 | dihydropyrimidinase | |  |
| PA2174 | -1.04912 | hypothetical protein | |  |
| PA3496 | -1.04936 | hypothetical protein | |  |
| PA0252 | -1.04945 | hypothetical protein | |  |
| PA0560 | -1.04985 | conserved hypothetical protein | | |
| PA3911 | -1.05376 | conserved hypothetical protein | | |
| *folE1* | -1.05425 | GTP cyclohydrolase I precursor | | |
| PA0507 | -1.05483 | probable acyl-CoA dehydrogenase | | |
| PA4343 | -1.05567 | probable major facilitator superfamily (MFS) transporter | | |
| PA4508 | -1.05575 | probable transcriptional regulator | | |
| PA1402 | -1.05714 | hypothetical protein | |  |
| PA1599 | -1.05728 | probable transcriptional regulator | | |
| PA2200 | -1.05926 | Cyclic-guanylate-specific phosphodiesterase | | |
| PA0984 | -1.06321 | colicin immunity protein | | |
| *ccmG* | -1.06632 | cytochrome C biogenesis protein CcmG | | |
| *aauR* | -1.06858 | AauR |  |  |
| PA0219 | -1.07026 | probable aldehyde dehydrogenase | | |
| PA1497 | -1.07403 | probable transporter | |  |
| PA5408 | -1.07574 | hypothetical protein | |  |
| *fosA* | -1.08092 | fosfomycin resistance protein, FosA | | |
| PA4153 | -1.08184 | 2,3-butanediol dehydrogenase | | |
| PA0714 | -1.08522 | hypothetical protein | |  |
| PA2210 | -1.08552 | probable major facilitator superfamily (MFS) transporter | | |
| *toxA* | -1.0861 | exotoxin A precursor | |  |
| *nrdJb* | -1.08959 | class II (cobalamin-dependent) ribonucleotide-diphosphate reductase subunit, NrdJb | | |
| *pqsB* | -1.09054 | PqsB |  |  |
| PA4531 | -1.09362 | hypothetical protein | |  |
| *pcaT* | -1.09365 | dicarboxylic acid transporter PcaT | | |
| PA2577 | -1.0973 | probable transcriptional regulator | | |
| *mexE* | -1.09826 | Resistance-Nodulation-Cell Division (RND) multidrug efflux membrane fusion protein MexE precursor | | |
| PA2499 | -1.09907 | probable deaminase | |  |
| PA0222 | -1.0992 | hypothetical protein | |  |
| *mdcE* | -1.09948 | malonate decarboxylase gamma subunit | | |
| PA4120 | -1.09952 | probable transcriptional regulator | | |
| *aphA* | -1.10049 | acetylpolyamine aminohydrolase | | |
| *liuB* | -1.10237 | methylcrotonyl-CoA carboxylase, beta-subunit | | |
| PA0797 | -1.11133 | probable transcriptional regulator | | |
| PA3378 | -1.11696 | conserved hypothetical protein | | |
| PA2450 | -1.11971 | hypothetical protein | |  |
| PA2213 | -1.12361 | probable porin | |  |
| *cupE1* | -1.12404 | Pilin subunit CupE1 | |  |
| *opdE* | -1.12485 | membrane protein OpdE | | |
| PA2162 | -1.1252 | probable glycosyl hydrolase | | |
| PA2326 | -1.12568 | hypothetical protein | |  |
| PA1507 | -1.12914 | probable transporter | |  |
| PA2155 | -1.13456 | probable phospholipase | | |
| PA1598 | -1.13622 | conserved hypothetical protein | | |
| PA1627 | -1.13747 | probable transcriptional regulator | | |
| *aroQ1* | -1.14196 | 3-dehydroquinate dehydratase | | |
| *tadA* | -1.14757 | TadA ATPase | |  |
| *acoB* | -1.14926 | acetoin catabolism protein AcoB | | |
| *fpvF* | -1.15351 | FpvF |  |  |
| PA1856 | -1.15946 | probable cytochrome oxidase subunit | | |
| P15 | -1.16008 | no |  |  |
| *pcaG* | -1.16457 | protocatechuate 3,4-dioxygenase, alpha subunit | | |
| *pcrG* | -1.16459 | regulator in type III secretion | | |
| *rgsA* | -1.16513 | no |  |  |
| *dppA1* | -1.16682 | probable binding protein component of ABC transporter | | |
| PA2318 | -1.16695 | hypothetical protein | |  |
| *potC* | -1.16844 | polyamine transport protein PotC | | |
| PA1233 | -1.16975 | hypothetical protein | |  |
| PA1316 | -1.16992 | probable major facilitator superfamily (MFS) transporter | | |
| PA2340 | -1.17022 | probable binding-protein-dependent maltose/mannitol transport protein | | |
| *fdnH* | -1.17172 | nitrate-inducible formate dehydrogenase, beta subunit | | |
| PA1541 | -1.17303 | probable drug efflux transporter | | |
| PA2094 | -1.17574 | probable transmembrane sensor | | |
| PA3912 | -1.17597 | conserved hypothetical protein | | |
| PA1499 | -1.18071 | conserved hypothetical protein | | |
| PA0939 | -1.18371 | hypothetical protein | |  |
| PA0742 | -1.18781 | hypothetical protein | |  |
| PA2674 | -1.18857 | probable type II secretion system protein | | |
| PA3281 | -1.19048 | hypothetical protein | |  |
| PA2658 | -1.19209 | hypothetical protein | |  |
| PA3780 | -1.19341 | hypothetical protein | |  |
| PA3500 | -1.19427 | conserved hypothetical protein | | |
| PA0800 | -1.19725 | hypothetical protein | |  |
| PA1410 | -1.19813 | probable periplasmic spermidine/putrescine-binding protein | | |
| PA1191 | -1.19941 | hypothetical protein | |  |
| PA3376 | -1.20079 | probable ATP-binding component of ABC transporter | | |
| PA4090 | -1.20709 | hypothetical protein | |  |
| PA3494 | -1.20853 | conserved hypothetical protein | | |
| *pvdN* | -1.20872 | PvdN |  |  |
| *tyrS* | -1.2104 | tyrosyl-tRNA synthetase | | |
| PA2114 | -1.21575 | probable major facilitator superfamily (MFS) transporter | | |
| *fliJ* | -1.21732 | flagellar protein FliJ | |  |
| PA4835 | -1.21809 | hypothetical protein | |  |
| *mtlD* | -1.21994 | mannitol dehydrogenase | | |
| PA0149 | -1.22678 | probable sigma-70 factor, ECF subfamily | | |
| *fdhA* | -1.23844 | glutathione-independent formaldehyde dehydrogenase | | |
| *popN* | -1.23947 | Type III secretion outer membrane protein PopN precursor | | |
| *ccoQ1* | -1.24033 | Cytochrome c oxidase, cbb3-type, CcoQ subunit | | |
| PA2838 | -1.24592 | probable transcriptional regulator | | |
| PA3967 | -1.24621 | hypothetical protein | |  |
| PA0719 | -1.24639 | hypothetical protein of bacteriophage Pf1 | | |
| PA0205 | -1.25444 | probable permease of ABC transporter | | |
| *algD* | -1.2554 | GDP-mannose 6-dehydrogenase AlgD | | |
| *fumC1* | -1.25548 | fumarate hydratase | |  |
| PA5431 | -1.25863 | probable transcriptional regulator | | |
| *pchF* | -1.26759 | pyochelin synthetase | |  |
| PA2835 | -1.26792 | probable major facilitator superfamily (MFS) transporter | | |
| PA2017 | -1.28158 | hypothetical protein | |  |
| PA4536 | -1.28391 | hypothetical protein | |  |
| PA2419 | -1.28503 | probable hydrolase | |  |
| *narL* | -1.28664 | two-component response regulator NarL | | |
| PA3773 | -1.28756 | hypothetical protein | |  |
| *flhB* | -1.29014 | flagellar biosynthetic protein FlhB | | |
| PA2062 | -1.29679 | probable pyridoxal-phosphate dependent enzyme | | |
| PA4836 | -1.3002 | hypothetical protein | |  |
| *pqsA* | -1.30026 | PqsA |  |  |
| PA5137 | -1.3161 | hypothetical protein | |  |
| PA3578 | -1.31944 | conserved hypothetical protein | | |
| *kdpE* | -1.31959 | two-component response regulator KdpE | | |
| PA3927 | -1.32666 | probable transcriptional regulator | | |
| PA4610 | -1.32688 | hypothetical protein | |  |
| PA1732 | -1.32689 | conserved hypothetical protein | | |
| *rhlA* | -1.32788 | rhamnosyltransferase chain A | | |
| PA3497 | -1.33035 | hypothetical protein | |  |
| PA1485 | -1.33054 | probable amino acid permease | | |
| PA3953 | -1.33166 | conserved hypothetical protein | | |
| PA4802 | -1.34451 | hypothetical protein | |  |
| *fprB* | -1.35966 | FprB |  |  |
| *azoR3* | -1.35967 | AzoR3, azoreductase 3 | | |
| *atuA* | -1.36329 | expressed protein with apparent function in citronellol catabolism | | |
| PA2837 | -1.3635 | probable outer membrane protein precursor | | |
| PA4979 | -1.36393 | probable acyl-CoA dehydrogenase | | |
| *folM* | -1.36524 | dihydromonapterin reductase, FolM | | |
| PA1265 | -1.36993 | hypothetical protein | |  |
| PA2097 | -1.37377 | probable flavin-binding monooxygenase | | |
| PA0151 | -1.37419 | probable TonB-dependent receptor | | |
| PA2112 | -1.37565 | conserved hypothetical protein | | |
| PA4681 | -1.37843 | hypothetical protein | |  |
| PA2022 | -1.37893 | probable nucleotide sugar dehydrogenase | | |
| *tpx* | -1.38449 | thiol peroxidase | |  |
| PA5486 | -1.38592 | conserved hypothetical protein | | |
| *betX* | -1.3921 | BetX |  |  |
| PA1280 | -1.39277 | hypothetical protein | |  |
| PA4940 | -1.39438 | conserved hypothetical protein | | |
| PA0138 | -1.39582 | probable permease of ABC transporter | | |
| *iscU* | -1.40211 | probable iron-binding protein IscU | | |
| PA1037 | -1.40406 | conserved hypothetical protein | | |
| PA1957 | -1.41147 | hypothetical protein | |  |
| *esrC* | -1.41291 | EsrC |  |  |
| PA2597 | -1.41347 | hypothetical protein | |  |
| *nrdD* | -1.41358 | class III (anaerobic) ribonucleoside-triphosphate reductase subunit, NrdD | | |
| PA5151 | -1.41378 | hypothetical protein | |  |
| *bauR* | -1.41835 | BauR |  |  |
| PA3509 | -1.41838 | probable hydrolase | |  |
| PA4114 | -1.42337 | lysine decarboxylase | |  |
| PA0734 | -1.4251 | hypothetical protein | |  |
| PA2607 | -1.426 | conserved hypothetical protein | | |
| PA1185 | -1.42904 | probable glutathione S-transferase | | |
| *sppB* | -1.43944 | ABC transporter permease, SppB | | |
| PA2937 | -1.44077 | hypothetical protein | |  |
| *foxI* | -1.44671 | ECF sigma factor FoxI | | |
| PA2124 | -1.44904 | probable dehydrogenase | | |
| PA5566 | -1.45077 | hypothetical protein | |  |
| PA0221 | -1.45171 | probable aminotransferase | | |
| PA2420 | -1.45349 | probable porin | |  |
| PA4128 | -1.4553 | conserved hypothetical protein | | |
| PA1285 | -1.4585 | probable transcriptional regulator | | |
| PA1112a | -1.47143 |  |  |  |
| *rhlC* | -1.47335 | rhamnosyltransferase 2 | | |
| *grxD* | -1.47637 | GrxD |  |  |
| PA4902 | -1.48046 | probable transcriptional regulator | | |
| *pauA* | -1.48715 | pimeloyl-CoA synthetase | | |
| *exbB1* | -1.50003 | transport protein ExbB | | |
| PA3178 | -1.50285 | hypothetical protein | |  |
| *fppA* | -1.50535 | Flp prepilin peptidase A, FppA | | |
| PA3865a | -1.51592 |  |  |  |
| *nagR* | -1.51824 | Transcriptional regulator of N-Acetylglucosamine catabolism operon | | |
| PA3196 | -1.51953 | hypothetical protein | |  |
| PA4193 | -1.535 | probable permease of ABC transporter | | |
| PA4849 | -1.53525 | hypothetical protein | |  |
| PA2662 | -1.54131 | conserved hypothetical protein | | |
| PA0226 | -1.56531 | probable CoA transferase, subunit A | | |
| *gidB* | -1.56724 | glucose inhibited division protein B | | |
| PA0200 | -1.58061 | hypothetical protein | |  |
| PA2055 | -1.58114 | probable major facilitator superfamily (MFS) transporter | | |
| *kdpC* | -1.58119 | potassium-transporting ATPase, C chain | | |
| PA2101 | -1.58549 | conserved hypothetical protein | | |
| PA2671 | -1.59298 | hypothetical protein | |  |
| PA0697 | -1.59824 | hypothetical protein | |  |
| *pvcC* | -1.60004 | paerucumarin biosynthesis protein PvcC | | |
| *arsB* | -1.60268 | ArsB protein | |  |
| PA4469 | -1.60327 | hypothetical protein | |  |
| PA0788a | -1.60498 |  |  |  |
| PA2661 | -1.61103 | hypothetical protein | |  |
| *hitA* | -1.61956 | ferric iron-binding periplasmic protein HitA | | |
| PA3734 | -1.62057 | hypothetical protein | |  |
| PA1406 | -1.62264 | hypothetical protein | |  |
| PA5540 | -1.62701 | hypothetical protein | |  |
| PA3400 | -1.63742 | hypothetical protein | |  |
| PA0107 | -1.63802 | conserved hypothetical protein | | |
| *nirB* | -1.64603 | assimilatory nitrite reductase large subunit | | |
| PA4861 | -1.64624 | probable ATP-binding component of ABC transporter | | |
| PA4155 | -1.6528 | hypothetical protein | |  |
| PA2437 | -1.65345 | hypothetical protein | |  |
| PA2374 | -1.66225 | hypothetical protein | |  |
| PA0258 | -1.67078 | hypothetical protein | |  |
| PA1977 | -1.68321 | hypothetical protein | |  |
| *foxR* | -1.68668 | Anti-sigma factor FoxR | | |
| *cdhC* | -1.68684 | CdhC, Carnitine dehydrogenase-related gene C | | |
| PA1026 | -1.69605 | hypothetical protein | |  |
| PA4591 | -1.70467 | hypothetical protein | |  |
| *fdx2* | -1.70915 | ferredoxin [2Fe-2S] | |  |
| *pchE* | -1.71633 | dihydroaeruginoic acid synthetase | | |
| PA0187 | -1.71683 | hypothetical protein | |  |
| *kynR* | -1.72261 | KynR |  |  |
| PA0918 | -1.72761 | cytochrome b561 | |  |
| PA4910 | -1.733 | branched chain amino acid ABC transporter ATP binding protein | | |
| *ampP* | -1.74674 | AmpP |  |  |
| *lhpP* | -1.74913 | ABC transporter periplasmic-binding protein, LhpP | | |
| PA2354 | -1.7576 | probable transcriptional regulator | | |
| PA0048 | -1.75818 | probable transcriptional regulator | | |
| *pfeR* | -1.76154 | two-component response regulator PfeR | | |
| *foxA* | -1.76572 | Ferrioxamine receptor FoxA | | |
| *femI* | -1.77965 | ECF sigma factor, FemI | | |
| *pcaK* | -1.78781 | 4-hydroxybenzoate transporter PcaK | | |
| *PA3881* | -1.79287 | hypothetical protein | |  |
| *nirQ* | -1.79385 | regulatory protein NirQ | | |
| PA3379 | -1.79603 | conserved hypothetical protein | | |
| PA2274 | -1.79971 | hypothetical protein | |  |
| *hemF* | -1.80544 | coproporphyrinogen III oxidase, aerobic | | |
| PA2048 | -1.81642 | hypothetical protein | |  |
| PA4680 | -1.81646 | hypothetical protein | |  |
| PA0749 | -1.81789 | hypothetical protein | |  |
| *tpbA* | -1.8191 | protein tyrosine phosphatase TpbA | | |
| PA0711 | -1.83282 | hypothetical protein | |  |
| PA5467 | -1.84048 | hypothetical protein | |  |
| PA2955 | -1.85178 | hypothetical protein | |  |
| *fecR* | -1.86132 | FecR |  |  |
| *pchR* | -1.86805 | transcriptional regulator PchR | | |
| PA3433 | -1.88507 | probable transcriptional regulator | | |
| PA0842 | -1.88536 | probable glycosyl transferase | | |
| PA4391 | -1.89012 | hypothetical protein | |  |
| PA0433 | -1.89532 | hypothetical protein | |  |
| PA2477 | -1.91068 | probable thiol:disulfide interchange protein | | |
| PA4828 | -1.91227 | conserved hypothetical protein | | |
| *fiuR* | -1.92788 | FiuR |  |  |
| PA3979 | -1.9295 | hypothetical protein | |  |
| *hsiA3* | -1.93266 | hypothetical protein | |  |
| *ptxR* | -1.93459 | transcriptional regulator PtxR | | |
| PA5183a | -1.94589 |  |  |  |
| PA4152 | -1.94662 | probable hydrolase | |  |
| PA2921 | -1.94663 | probable transcriptional regulator | | |
| PA0477 | -1.95153 | probable transcriptional regulator | | |
| *pscT* | -1.96983 | translocation protein in type III secretion | | |
| PA1329 | -1.97158 | conserved hypothetical protein | | |
| *pscG* | -1.98749 | type III export protein PscG | | |
| PA5032 | -1.99351 | probable transcriptional regulator | | |
| PA0124 | -1.99527 | hypothetical protein | |  |
| *cynT* | -1.99743 | carbonate dehydratase | | |
| *pchA* | -2.00594 | salicylate biosynthesis isochorismate synthase | | |
| PA0708 | -2.02294 | probable transcriptional regulator | | |
| PA5275 | -2.0259 | conserved hypothetical protein | | |
| PA0940 | -2.03752 | hypothetical protein | |  |
| PA3871 | -2.04917 | probable peptidyl-prolyl cis-trans isomerase, PpiC-type | | |
| PA2192 | -2.04924 | conserved hypothetical protein | | |
| *feoB* | -2.05757 | FeoB |  |  |
| PA0521 | -2.06873 | probable cytochrome c oxidase subunit | | |
| PA2681 | -2.0842 | probable transcriptional regulator | | |
| *shaE* | -2.09936 | ShaE |  |  |
| *sphA* | -2.11377 | SphA |  |  |
| PA2801 | -2.11496 | hypothetical protein | |  |
| PA2878 | -2.13188 | hypothetical protein | |  |
| *exaA* | -2.15769 | quinoprotein ethanol dehydrogenase | | |
| *hpaC* | -2.1844 | 4-hydroxyphenylacetate 3-monooxygenase small chain | | |
| PA3386 | -2.18947 | conserved hypothetical protein | | |
| PA1906 | -2.19795 | hypothetical protein | |  |
| PA3884 | -2.20281 | hypothetical protein | |  |
| PA1186 | -2.20655 | hypothetical protein | |  |
| PA2109 | -2.2072 | hypothetical protein | |  |
| *tonB2* | -2.21802 | TonB2 |  |  |
| PA1234 | -2.24568 | hypothetical protein | |  |
| PA2376 | -2.24725 | probable transcriptional regulator | | |
| PA2031 | -2.26613 | hypothetical protein | |  |
| PA1300 | -2.28723 | ECF sigma factor | |  |
| PA0613 | -2.33113 | hypothetical protein | |  |
| PA4883 | -2.33134 | hypothetical protein | |  |
| *pchG* | -2.3411 | pyochelin biosynthetic protein PchG | | |
| PA2004 | -2.34501 | conserved hypothetical protein | | |
| PA1385 | -2.34775 | probable glycosyl transferase | | |
| *moaA1* | -2.34891 | molybdopterin biosynthetic protein A1 | | |
| PA4171 | -2.37393 | probable protease | |  |
| *ppyR* | -2.3765 | psl and pyoverdine operon regulator, PpyR | | |
| *hxcR* | -2.40705 | HxcR |  |  |
| PA1937 | -2.40931 | conserved hypothetical protein | | |
| PA4167 | -2.43323 | probable oxidoreductase | | |
| PA0034 | -2.45794 | probable two-component response regulator | | |
| PA0273 | -2.47241 | probable major facilitator superfamily (MFS) transporter | | |
| PA2090 | -2.4968 | hypothetical protein | |  |
| *shaB* | -2.50827 | ShaB |  |  |
| *ampO* | -2.51205 | AmpO |  |  |
| *ppiC1* | -2.52897 | peptidyl-prolyl cis-trans isomerase C1 | | |
| *narI* | -2.53093 | respiratory nitrate reductase gamma chain | | |
| PA0882 | -2.53959 | hypothetical protein | |  |
| *pqsE* | -2.55153 | Quinolone signal response protein | | |
| *fepC* | -2.56055 | ferric enterobactin transport protein FepC | | |
| PA4038 | -2.56532 | hypothetical protein | |  |
| *narJ* | -2.632 | respiratory nitrate reductase delta chain | | |
| PA3733a | -2.80467 |  |  |  |
| *fptA* | -2.80867 | Fe(III)-pyochelin outer membrane receptor precursor | | |
| PA2179 | -2.82282 | hypothetical protein | |  |
| PA0525 | -2.82823 | probable dinitrification protein NorD | | |
| PA5217 | -2.82965 | probable binding protein component of ABC iron transporter | | |
| *tonB1* | -2.84864 | TonB1 |  |  |
| PA1221 | -2.91112 | hypothetical protein | |  |
| *narH* | -2.93199 | respiratory nitrate reductase beta chain | | |
| PA2762 | -2.98908 | hypothetical protein | |  |
| PA0753 | -3.07831 | hypothetical protein | |  |
| *fiuI* | -3.17203 | FiuI |  |  |
| *femR* | -3.19023 | sigma factor regulator, FemR | | |
| PA1131 | -3.22071 | probable major facilitator superfamily (MFS) transporter | | |
| *exbD1* | -3.25812 | transport protein ExbD | | |
| PA4806 | -3.50783 | probable transcriptional regulator | | |
| *pchD* | -3.65351 | pyochelin biosynthesis protein PchD | | |
| *norB* | -3.75142 | nitric-oxide reductase subunit B | | |
| *narG* | -3.79419 | respiratory nitrate reductase alpha chain | | |
| *fhp* | -3.93058 | flavohemoprotein | |  |
| *moaB1* | -3.97084 | molybdopterin biosynthetic protein B1 | | |
| *norC* | -4.0164 | nitric-oxide reductase subunit C | | |
| PA0929 | -4.15228 | two-component response regulator | | |
| *narK1* | -4.79884 | nitrite extrusion protein 1 | | |
| *moeA1* | -4.81557 | molybdenum cofactor biosynthetic protein A1 | | |
| *narK2* | -4.83938 | nitrite extrusion protein 2 | | |

**Table S1D. Differential expression of 749 genes in *Pseudomonas aeruginosa* PAO1 under the anaerobic condition.**

| **Gene** | **Δ*anvM* VS WT log2(fold_change)** | **Function** |  |
| --- | --- | --- | --- |
| PA2538 | 3.63779 | hypothetical protein | |
| PA5031 | 3.27429 | probable short chain dehydrogenase | |
| PA4222 | 3.22477 | probable ATP-binding component of ABC transporter | |
| PA1331 | 3.19655 | conserved hypothetical protein | |
| PA1313 | 3.05501 | probable major facilitator superfamily (MFS) transporter | |
| PA0589 | 3.05435 | conserved hypothetical protein | |
| PA4106 | 2.97704 | conserved hypothetical protein | |
| *pelD* | 2.97406 | PelD |  |
| PA4469 | 2.95009 | hypothetical protein | |
| PA2092 | 2.85304 | probable major facilitator superfamily (MFS) transporter | |
| PA2698 | 2.75249 | probable hydrolase | |
| *ohr* | 2.70673 | organic hydroperoxide resistance protein | |
| PA4508 | 2.69752 | probable transcriptional regulator | |
| *cupE1* | 2.67952 | Pilin subunit CupE1 | |
| PA0174 | 2.64732 | conserved hypothetical protein | |
| PA2339 | 2.60428 | probable binding-protein-dependent maltose/mannitol transport protein | |
| *lhpA* | 2.56903 | Hydroxyproline 2-epimerase, LhpA | |
| PA1489 | 2.5424 | hypothetical protein | |
| PA1034 | 2.53056 | hypothetical protein | |
| PA1866a | 2.51904 |  |  |
| PA2589 | 2.51686 | hypothetical protein | |
| *lhpC* | 2.51078 | delta1-pyrroline-4-hydroxy-2-carboxylate deaminase, LphC | |
| *lyxD* | 2.50815 | l-lyxonate dehydratase LyxD | |
| *xenB* | 2.48865 | xenobiotic reductase | |
| *fosA* | 2.42775 | fosfomycin resistance protein, FosA | |
| *rgsA* | 2.41473 | no |  |
| PA4537 | 2.40096 | hypothetical protein | |
| *hdhA* | 2.37189 | hydrazone dehydrogenase, HdhA | |
| *hsiB3* | 2.3536 | HsiB3 |  |
| *exaC* | 2.33523 | NAD+ dependent aldehyde dehydrogenase ExaC | |
| PA3383 | 2.3255 | binding protein component of ABC phosphonate transporter | |
| *lhpB* | 2.32308 | D-hydroxyproline dehydrogenase beta-subunit, LphB | |
| PA4037 | 2.31833 | probable ATP-binding component of ABC transporter | |
| *oprP* | 2.31022 | Phosphate-specific outer membrane porin OprP precursor | |
| *nosF* | 2.29433 | NosF protein | |
| *fepB* | 2.29275 | ferrienterobactin-binding periplasmic protein precursor FepB | |
| *pvdR* | 2.28678 | PvdR |  |
| PA5397 | 2.28573 | hypothetical protein | |
| *fpvC* | 2.28209 | FpvC |  |
| PA0209 | 2.27339 | conserved hypothetical protein | |
| *cobD* | 2.27282 | cobalamin biosynthetic protein CobD | |
| PA0702 | 2.26881 | hypothetical protein | |
| *cobQ* | 2.26473 | cobyric acid synthase | |
| PA5071 | 2.25889 | conserved hypothetical protein | |
| PA0385 | 2.25265 | hypothetical protein | |
| PA0241 | 2.24466 | probable major facilitator superfamily (MFS) transporter | |
| PA2066 | 2.23767 | hypothetical protein | |
| PA3252 | 2.23713 | probable permease of ABC transporter | |
| PA4644 | 2.2366 | hypothetical protein | |
| PA0752 | 2.23103 | conserved hypothetical protein | |
| PA5543 | 2.23082 | hypothetical protein | |
| PA1922 | 2.22248 | probable TonB-dependent receptor | |
| *pvcD* | 2.21327 | paerucumarin biosynthesis protein PvcD | |
| PA0957 | 2.18557 | hypothetical protein | |
| PA4909 | 2.17513 | probable ATP-binding component of ABC transporter | |
| PA1963 | 2.17221 | hypothetical protein | |
| PA3403 | 2.16952 | hypothetical protein | |
| PA1500 | 2.16803 | probable oxidoreductase | |
| PA3057 | 2.1419 | hypothetical protein | |
| PA3963a | 2.10692 |  |  |
| PA2448 | 2.09697 | hypothetical protein | |
| *flgF* | 2.04754 | flagellar basal-body rod protein FlgF | |
| PA0346 | 2.04721 | hypothetical protein | |
| PA1828 | 2.02943 | probable short-chain dehydrogenase | |
| PA0118 | 2.02735 | hypothetical protein | |
| PA4680 | 2.02372 | hypothetical protein | |
| PA3249 | 2.02032 | probable transcriptional regulator | |
| PA2747a | 2.00103 |  |  |
| PA4712 | 1.99669 | hypothetical protein | |
| PA0261 | 1.97179 | hypothetical protein | |
| PA2784 | 1.91606 | hypothetical protein | |
| PA0213 | 1.88819 | hypothetical protein | |
| PA3389 | 1.86936 | probable ring-cleaving dioxygenase | |
| *narI* | 1.86625 | respiratory nitrate reductase gamma chain | |
| *mdcD* | 1.86034 | malonate decarboxylase beta subunit | |
| PA3869 | 1.85721 | hypothetical protein | |
| *algE* | 1.85093 | Alginate production outer membrane protein AlgE precursor | |
| PA0881 | 1.83247 | hypothetical protein | |
| *tssG1* | 1.82085 | TssG1 |  |
| *fapB* | 1.81953 | FapB |  |
| PA2670 | 1.81523 | hypothetical protein | |
| *algF* | 1.81473 | alginate o-acetyltransferase AlgF | |
| PA4098 | 1.80562 | probable short-chain dehydrogenase | |
| *trx2* | 1.77394 | Trx2 |  |
| PA0540 | 1.7699 | hypothetical protein | |
| *rhlI* | 1.76341 | autoinducer synthesis protein RhlI | |
| PA3330 | 1.75952 | probable short chain dehydrogenase | |
| PA2677 | 1.75332 | probable type II secretion protein | |
| PA2325 | 1.75331 | hypothetical protein | |
| PA3938 | 1.73982 | probable periplasmic taurine-binding protein precursor | |
| PA0306a | 1.73872 |  |  |
| *ohrR* | 1.7367 | OhrR |  |
| PA3791 | 1.73531 | hypothetical protein | |
| PA1314 | 1.72813 | hypothetical protein | |
| *pvdT* | 1.72759 | PvdT |  |
| PA0701 | 1.72185 | probable transcriptional regulator | |
| PA2723 | 1.71992 | hypothetical protein | |
| *pqsL* | 1.71827 | probable FAD-dependent monooxygenase | |
| *arnD* | 1.7091 | ArnD |  |
| PA4800 | 1.70618 | hypothetical protein | |
| *eagT6* | 1.69342 | EagT6 |  |
| PA4466 | 1.6907 | probable phosphoryl carrier protein | |
| PA4862 | 1.69039 | probable ATP-binding component of ABC transporter | |
| PA3381 | 1.68775 | probable transcriptional regulator | |
| PA3334 | 1.68605 | probable acyl carrier protein | |
| *glpD* | 1.68596 | glycerol-3-phosphate dehydrogenase | |
| PA0602 | 1.67749 | probable binding protein component of ABC transporter | |
| PA2673 | 1.65281 | probable type II secretion system protein | |
| PA0613 | 1.65132 | hypothetical protein | |
| PA3668 | 1.65031 | conserved hypothetical protein | |
| PA4172 | 1.64827 | probable nuclease | |
| *alkB1* | 1.64653 | alkane-1-monooxygenase | |
| PA3774 | 1.64518 | histone deacetylase-like amidohydrolase | |
| PA0842 | 1.63612 | probable glycosyl transferase | |
| PA2453 | 1.63495 | hypothetical protein | |
| *cyoA* | 1.63469 | cytochrome o ubiquinol oxidase subunit II | |
| *hisP* | 1.63213 | histidine transport protein HisP | |
| PA4836 | 1.62332 | hypothetical protein | |
| PA3254 | 1.61478 | probable ATP-binding component of ABC transporter | |
| PA0753 | 1.61057 | hypothetical protein | |
| PA0190 | 1.60935 | probable acid phosphatase | |
| *fecI* | 1.59948 | FecI |  |
| PA2073 | 1.59766 | probable transporter (membrane subunit) | |
| PA0775 | 1.59331 | conserved hypothetical protein | |
| *pscU* | 1.59173 | translocation protein in type III secretion | |
| PA2307 | 1.58859 | probable permease of ABC transporter | |
| PA4121 | 1.58613 | conserved hypothetical protein | |
| PA4090 | 1.57558 | hypothetical protein | |
| PA2719 | 1.57094 | hypothetical protein | |
| PA2980 | 1.57072 | conserved hypothetical protein | |
| PA5467 | 1.5642 | hypothetical protein | |
| PA0720 | 1.56405 | helix destabilizing protein of bacteriophage Pf1 | |
| PA3773 | 1.54343 | hypothetical protein | |
| PA2116 | 1.53035 | conserved hypothetical protein | |
| PA2754a | 1.52858 |  |  |
| *sphA* | 1.51874 | SphA |  |
| PA2536 | 1.51862 | probable phosphatidate cytidylyltransferase | |
| PA4096 | 1.51538 | probable major facilitator superfamily (MFS) transporter | |
| *ambA* | 1.51478 | AmbA |  |
| PA1649 | 1.49697 | probable short-chain dehydrogenase | |
| PA2084 | 1.49516 | probable asparagine synthetase | |
| PA5404 | 1.49409 | hypothetical protein | |
| PA5282 | 1.47672 | probable major facilitator superfamily (MFS) transporter | |
| PA2314 | 1.47129 | probable major facilitator superfamily (MFS) transporter | |
| PA0270 | 1.46909 | hypothetical protein | |
| *folM* | 1.46731 | dihydromonapterin reductase, FolM | |
| PA3492 | 1.45155 | conserved hypothetical protein | |
| PA1238 | 1.45039 | probable outer membrane component of multidrug efflux pump | |
| *bfd* | 1.44822 | bacterioferritin-associated ferredoxin Bfd | |
| PA1020 | 1.44328 | probable acyl-CoA dehydrogenase | |
| PA4908 | 1.44222 | hypothetical protein | |
| PA2560 | 1.43674 | hypothetical protein | |
| PA4371 | 1.43544 | hypothetical protein | |
| PA3436 | 1.43536 | hypothetical protein | |
| *vgrG4a* | 1.43246 | VgrG4a |  |
| *arnA* | 1.42982 | ArnA |  |
| PA0640 | 1.41953 | probable bacteriophage protein | |
| *dksA2* | 1.41312 | DksA2 |  |
| *bswR* | 1.41008 | bacterial swarming regulator BswR | |
| PA0811 | 1.40561 | probable major facilitator superfamily (MFS) transporter | |
| PA0630 | 1.40433 | hypothetical protein | |
| *recD* | 1.39754 | exodeoxyribonuclease V alpha chain | |
| PA2165 | 1.39024 | probable glycogen synthase | |
| PA2804 | 1.38763 | hypothetical protein | |
| PA3462 | 1.37359 | probable sensor/response regulator hybrid | |
| PA2716 | 1.37301 | probable FMN oxidoreductase | |
| PA5180 | 1.36941 | conserved hypothetical protein | |
| PA4900 | 1.36837 | probable major facilitator superfamily (MFS) transporter | |
| PA5081 | 1.36464 | hypothetical protein | |
| PA0671 | 1.35983 | hypothetical protein | |
| *napC* | 1.35354 | cytochrome c-type protein NapC | |
| PA3808 | 1.34887 | conserved hypothetical protein | |
| PA1226 | 1.34644 | probable transcriptional regulator | |
| *pqqB* | 1.32944 | pyrroloquinoline quinone biosynthesis protein B | |
| PA2230 | 1.32942 | hypothetical protein | |
| PA2296 | 1.32849 | hypothetical protein | |
| *endA* | 1.32391 | DNA-specific endonuclease I | |
| PA2184 | 1.32307 | conserved hypothetical protein | |
| *hudR* | 1.32043 | HudR |  |
| PA3509 | 1.31864 | probable hydrolase | |
| *glpQ* | 1.31748 | glycerophosphoryl diester phosphodiesterase, periplasmic | |
| PA5030 | 1.31309 | probable major facilitator superfamily (MFS) transporter | |
| *ercS'* | 1.30773 | ErcS' |  |
| PA5459 | 1.30667 | putative methyltransferase | |
| PA3207 | 1.30097 | hypothetical protein | |
| *pfeR* | 1.30004 | two-component response regulator PfeR | |
| PA2881 | 1.29636 | probable two-component response regulator | |
| *narH* | 1.29565 | respiratory nitrate reductase beta chain | |
| *ccmG* | 1.2849 | cytochrome C biogenesis protein CcmG | |
| PA4605 | 1.28451 | conserved hypothetical protein | |
| PA2910 | 1.28439 | conserved hypothetical protein | |
| PA3378 | 1.28244 | conserved hypothetical protein | |
| PA2914 | 1.28198 | probable permease of ABC transporter | |
| PA0271 | 1.28156 | hypothetical protein | |
| PA1388 | 1.27995 | hypothetical protein | |
| PA2909 | 1.27862 | hypothetical protein | |
| PA1123 | 1.27697 | hypothetical protein | |
| PA3027 | 1.27469 | probable transcriptional regulator | |
| PA2807 | 1.27352 | hypothetical protein | |
| *cobH* | 1.27328 | precorrin isomerase CobH | |
| PA1993 | 1.27212 | probable major facilitator superfamily (MFS) transporter | |
| PA1859 | 1.26875 | probable transcriptional regulator | |
| PA2848 | 1.26759 | probable transcriptional regulator | |
| PA3089a | 1.26342 |  |  |
| PA1350 | 1.26328 | hypothetical protein | |
| PA5362 | 1.26245 | conserved hypothetical protein | |
| PA2823 | 1.26157 | conserved hypothetical protein | |
| PA3601 | 1.2605 | conserved hypothetical protein | |
| PA1038 | 1.25409 | hypothetical protein | |
| *rhlC* | 1.25222 | rhamnosyltransferase 2 | |
| PA0380 | 1.24742 | conserved hypothetical protein | |
| PA2192 | 1.24417 | conserved hypothetical protein | |
| PA2898 | 1.24156 | hypothetical protein | |
| PA0171 | 1.24058 | hypothetical protein | |
| PA2097 | 1.23703 | probable flavin-binding monooxygenase | |
| PA1398 | 1.23667 | hypothetical protein | |
| PA0054 | 1.2356 | conserved hypothetical protein | |
| *dsbM* | 1.23535 | DsbM |  |
| *lldA* | 1.2344 | L-lactate dehydrogenase | |
| PA3419 | 1.23416 | hypothetical protein | |
| PA1651 | 1.23344 | probable transporter | |
| PA1224 | 1.23243 | probable NAD(P)H dehydrogenase | |
| PA3788 | 1.23153 | hypothetical protein | |
| *ptpA* | 1.22746 | phosphotyrosine protein phosphatase | |
| PA1299 | 1.22662 | conserved hypothetical protein | |
| *exsB* | 1.2256 | exoenzyme S synthesis protein B | |
| PA2710 | 1.22456 | hypothetical protein | |
| PA3510 | 1.22424 | hypothetical protein | |
| PA1412 | 1.22414 | hypothetical protein | |
| PA0900 | 1.22337 | hypothetical protein | |
| PA1652 | 1.22301 | hypothetical protein | |
| PA2098 | 1.21822 | probable esterase/deacetylase | |
| PA3747 | 1.21635 | conserved hypothetical protein | |
| PA0322 | 1.2107 | probable transporter | |
| PA0544 | 1.2104 | hypothetical protein | |
| PA3922 | 1.20532 | conserved hypothetical protein | |
| PA1390 | 1.20377 | probable glycosyl transferase | |
| PA5535 | 1.20122 | conserved hypothetical protein | |
| PA2167 | 1.19965 | hypothetical protein | |
| PA4690a | 1.19772 |  |  |
| *crfX* | 1.19705 | CrfX protein | |
| PA2718 | 1.19061 | probable transcriptional regulator | |
| PA2216 | 1.18833 | 2-keto-3-deoxy-D-arabinonate dehydratase | |
| PA1170 | 1.18259 | conserved hypothetical protein | |
| PA4103 | 1.18057 | hypothetical protein | |
| PA3035 | 1.17782 | probable glutathione S-transferase | |
| PA4321 | 1.17761 | hypothetical protein | |
| *pslL* | 1.17721 | hypothetical protein | |
| PA5340 | 1.17585 | hypothetical protein | |
| *hxcV* | 1.17369 | HxcV |  |
| *lasB* | 1.16919 | elastase LasB | |
| PA4060 | 1.16799 | hypothetical protein | |
| *ampR* | 1.1671 | transcriptional regulator AmpR | |
| *hcnC* | 1.16629 | hydrogen cyanide synthase HcnC | |
| PA4338 | 1.16474 | hypothetical protein | |
| PA2845 | 1.16342 | hypothetical protein | |
| *cysC* | 1.16073 | adenosine 5'-phosphosulfate (APS) kinase | |
| PA3129 | 1.16054 | conserved hypothetical protein | |
| PA1039 | 1.15684 | conserved hypothetical protein | |
| PA0940 | 1.15615 | hypothetical protein | |
| *pqsA* | 1.15592 | PqsA |  |
| PA2317 | 1.1509 | probable oxidoreductase | |
| PA0954 | 1.14775 | probable acylphosphatase | |
| *rubA2* | 1.14589 | Rubredoxin 2 | |
| PA2901 | 1.13818 | hypothetical protein | |
| PA5507 | 1.13175 | hypothetical protein | |
| *chpC* | 1.13058 | probable chemotaxis protein | |
| PA3130 | 1.12881 | hypothetical protein | |
| PA1521 | 1.12794 | probable guanine deaminase | |
| *aroP2* | 1.12733 | aromatic amino acid transport protein AroP2 | |
| *hpcB* | 1.11774 | homoprotocatechuate 2,3-dioxygenase | |
| *rsmZ* | 1.11574 | no |  |
| *fhp* | 1.11387 | flavohemoprotein | |
| PA2163 | 1.11236 | hypothetical protein | |
| *algX* | 1.11067 | alginate biosynthesis protein AlgX | |
| PA2217 | 1.11002 | probable aldehyde dehydrogenase | |
| PA2602 | 1.1081 | 3-mercaptopropionate dioxygenase | |
| PA2799 | 1.10702 | hypothetical protein | |
| PA3036 | 1.10686 | hypothetical protein | |
| PA4698 | 1.1014 | hypothetical protein | |
| *nrdG* | 1.10014 | class III (anaerobic) ribonucleoside-triphosphate reductase activating protein, 'activase', NrdG | |
| PA1578 | 1.09861 | hypothetical protein | |
| PA1108 | 1.09584 | probable major facilitator superfamily (MFS) transporter | |
| *prtN* | 1.09561 | transcriptional regulator PrtN | |
| PA1573 | 1.08992 | conserved hypothetical protein | |
| PA0571 | 1.08756 | hypothetical protein | |
| PA4330 | 1.08696 | probable enoyl-CoA hydratase/isomerase | |
| PA2047 | 1.08554 | probable transcriptional regulator | |
| PA5547 | 1.08468 | conserved hypothetical protein | |
| PA2658 | 1.07998 | hypothetical protein | |
| *erbR* | 1.07976 | response regulator ErbR | |
| PA4319 | 1.07522 | conserved hypothetical protein | |
| *fdhA* | 1.06858 | glutathione-independent formaldehyde dehydrogenase | |
| PA3679 | 1.06778 | hypothetical protein | |
| PA4046 | 1.06632 | hypothetical protein | |
| PA4828 | 1.06627 | conserved hypothetical protein | |
| *alkB2* | 1.05922 | alkane-1-monooxygenase 2 | |
| *ppiA* | 1.05843 | peptidyl-prolyl cis-trans isomerase A | |
| PA1330 | 1.05703 | probable short-chain dehydrogenase | |
| PA3233 | 1.05286 | hypothetical protein | |
| PA3359 | 1.05005 | hypothetical protein | |
| PA3420 | 1.04307 | probable transcriptional regulator | |
| PA4534 | 1.04089 | hypothetical protein | |
| *rluA* | 1.03952 | pseudouridine synthase RluA | |
| PA3498 | 1.03762 | probable oxidoreductase | |
| *lhpH* | 1.03698 | LhpH |  |
| *glyA2* | 1.03147 | serine hydroxymethyltransferase | |
| PA1304 | 1.03053 | probable oligopeptidase | |
| PA3285 | 1.03026 | probable sigma-70 factor, ECF subfamily | |
| PA0046 | 1.02983 | hypothetical protein | |
| PA0192 | 1.02756 | probable TonB-dependent receptor | |
| PA1957 | 1.02402 | hypothetical protein | |
| *fleS* | 1.02372 | two-component sensor | |
| *pilR* | 1.02149 | two-component response regulator PilR | |
| PA3022 | 1.02105 | hypothetical protein | |
| *shaF* | 1.0206 | ShaF |  |
| *pchP* | 1.01988 | phosphorylcholine phosphatase | |
| PA3955 | 1.0197 | hypothetical protein | |
| PA3473 | 1.01893 | hypothetical protein | |
| PA2993 | 1.01596 | conserved hypothetical protein | |
| PA1923 | 1.01527 | hypothetical protein | |
| PA4840 | 1.01451 | conserved hypothetical protein | |
| PA2094 | 1.01276 | probable transmembrane sensor | |
| PA5021 | 1.00876 | probable sodium/hydrogen antiporter | |
| PA2218 | 1.00654 | hypothetical protein | |
| PA3579 | 1.00501 | probable carbohydrate kinase | |
| *cobJ* | 1.00062 | precorrin-3 methylase CobJ | |
| *nirN* | -1.00007 | NirN |  |
| *cupE6* | -1.00116 | Adhesin-like protein CupE6 | |
| PA1611 | -1.00248 | hybrid sensor kinase | |
| *hxcT* | -1.00492 | HxcT |  |
| PA1287 | -1.007 | probable glutathione peroxidase | |
| PA1306 | -1.00809 | probable HIT family protein | |
| *ureF* | -1.00897 | urease accessory protein UreF | |
| PA5393 | -1.01045 | conserved hypothetical protein | |
| *ilvA2* | -1.01055 | threonine dehydratase, biosynthetic | |
| *bfmR* | -1.01241 | BfmR |  |
| PA2421 | -1.01277 | hypothetical protein | |
| PA4131 | -1.01309 | probable iron-sulfur protein | |
| PA5478 | -1.01509 | conserved hypothetical protein | |
| PA0397 | -1.01554 | probable cation efflux system protein | |
| PA5233 | -1.02176 | hypothetical protein | |
| PA4816 | -1.02237 | hypothetical protein | |
| PA3075 | -1.02342 | hypothetical protein | |
| PA3433 | -1.02554 | probable transcriptional regulator | |
| *vgrG6* | -1.02653 | VgrG6 |  |
| PA2202 | -1.02953 | probable amino acid permease | |
| *xerD* | -1.03082 | integrase/recombinase XerD | |
| PA4955 | -1.0326 | hypothetical protein | |
| PA2124 | -1.03323 | probable dehydrogenase | |
| *pqsC* | -1.03515 | PqsC |  |
| PA1854 | -1.03541 | conserved hypothetical protein | |
| PA0657 | -1.03751 | probable ATPase | |
| *coxA* | -1.03901 | cytochrome c oxidase, subunit I | |
| PA0028 | -1.04416 | hypothetical protein | |
| PA3268 | -1.05172 | probable TonB-dependent receptor | |
| *cosX* | -1.05208 | CosX |  |
| PA0184 | -1.05215 | probable ATP-binding component of ABC transporter | |
| PA0769 | -1.06643 | hypothetical protein | |
| PA0240 | -1.06648 | probable porin | |
| *bamI* | -1.06732 | biofilm-associated metzincin Inhibitor, BamI | |
| PA2252 | -1.06772 | probable AGCS sodium/alanine/glycine symporter | |
| PA1646 | -1.0691 | probable chemotaxis transducer | |
| PA3377 | -1.07199 | conserved hypothetical protein | |
| PA2095 | -1.07207 | hypothetical protein | |
| PA1404 | -1.07218 | hypothetical protein | |
| PA1878 | -1.07223 | hypothetical protein | |
| PA0043 | -1.07239 | hypothetical protein | |
| *flhA* | -1.07402 | flagellar biosynthesis protein FlhA | |
| PA2489 | -1.07446 | probable transcriptional regulator | |
| PA0110 | -1.0764 | hypothetical protein | |
| PA1451 | -1.07675 | conserved hypothetical protein | |
| PA0701a | -1.07714 |  |  |
| *rnk* | -1.07752 | nucleoside diphosphate kinase regulator | |
| PA4620 | -1.07822 | hypothetical protein | |
| PA3513 | -1.08106 | hypothetical protein | |
| PA1648 | -1.08243 | probable oxidoreductase | |
| *rimI* | -1.08489 | peptide n-acetyltransferase RimI | |
| PA1052a | -1.0866 |  |  |
| *braD* | -1.08713 | branched-chain amino acid transport protein BraD | |
| PA5271 | -1.08817 | hypothetical protein | |
| PA4887 | -1.08896 | probable major facilitator superfamily (MFS) transporter | |
| PA0566 | -1.08988 | hypothetical protein | |
| PA1347 | -1.09205 | probable transcriptional regulator | |
| *azoR3* | -1.09229 | AzoR3, azoreductase 3 | |
| *rrmA* | -1.09353 | rRNA methyltransferase | |
| PA4143 | -1.09498 | probable toxin transporter | |
| PA2471 | -1.09548 | conserved hypothetical protein | |
| *oprN* | -1.09679 | Multidrug efflux outer membrane protein OprN precursor | |
| *vanA* | -1.09806 | vanillate O-demethylase oxygenase subunit | |
| PA2839 | -1.10038 | conserved hypothetical protein | |
| *atsA* | -1.10052 | arylsulfatase | |
| P15 | -1.10084 | no |  |
| PA2874 | -1.10408 | hypothetical protein | |
| *pykF* | -1.10508 | pyruvate kinase I | |
| PA2781 | -1.1059 | hypothetical protein | |
| PA0348 | -1.11396 | hypothetical protein | |
| *nppD* | -1.11402 | NppD |  |
| PA1740 | -1.11579 | hypothetical protein | |
| PA0222 | -1.11616 | hypothetical protein | |
| PA1364 | -1.11671 | probable transmembrane sensor | |
| PA0864 | -1.11673 | probable transcriptional regulator | |
| PA4150 | -1.1217 | probable dehydrogenase E1 component | |
| PA4111 | -1.12533 | hypothetical protein | |
| *cobO* | -1.12897 | cob(I)alamin adenosyltransferase | |
| PA4536 | -1.12925 | hypothetical protein | |
| *tpbA* | -1.13016 | protein tyrosine phosphatase TpbA | |
| *phoR* | -1.13082 | two-component sensor PhoR | |
| *pvdN* | -1.13133 | PvdN |  |
| PA4894 | -1.13388 | hypothetical protein | |
| PA0223 | -1.13509 | probable dihydrodipicolinate synthetase | |
| PA1929 | -1.13657 | hypothetical protein | |
| *cpo* | -1.13666 | chloroperoxidase precursor | |
| *lip3* | -1.13743 | Lip3 |  |
| *lhpP* | -1.13758 | ABC transporter periplasmic-binding protein, LhpP | |
| PA3449 | -1.13948 | conserved hypothetical protein | |
| PA0481 | -1.14481 | hypothetical protein | |
| PA2077 | -1.14561 | oleate 10S-lipoxygenase | |
| PA5520 | -1.14741 | hypothetical protein | |
| PA2706 | -1.15078 | hypothetical protein | |
| *pcaQ* | -1.15183 | transcriptional regulator PcaQ | |
| *hxcR* | -1.15277 | HxcR |  |
| PA0708 | -1.15307 | probable transcriptional regulator | |
| PA3675 | -1.15458 | hypothetical protein | |
| PA0738 | -1.15531 | conserved hypothetical protein | |
| PA0665 | -1.15541 | conserved hypothetical protein | |
| PA5310 | -1.15904 | conserved hypothetical protein | |
| *fliN* | -1.15945 | flagellar motor switch protein FliN | |
| PA2329 | -1.16163 | probable ATP-binding component of ABC transporter | |
| PA3203 | -1.16311 | hypothetical protein | |
| *opmE* | -1.16461 | OpmE |  |
| PA1870 | -1.16838 | hypothetical protein | |
| *pqqE* | -1.17152 | pyrroloquinoline quinone biosynthesis protein E | |
| PA3003 | -1.17282 | hypothetical protein | |
| *pvcB* | -1.17283 | paerucumarin biosynthesis protein PvcB | |
| PA4796 | -1.1735 | hypothetical protein | |
| *iciA* | -1.17392 | inhibitor of chromosome initiation IciA | |
| *mobA* | -1.1742 | molybdopterin-guanine dinucleotide biosynthesis protein MobA | |
| PA0735 | -1.17501 | hypothetical protein | |
| *tgpA* | -1.17584 | transglutaminase protein A, TgpA | |
| PA1265 | -1.17643 | hypothetical protein | |
| *rubA1* | -1.17693 | Rubredoxin 1 | |
| PA2179 | -1.18201 | hypothetical protein | |
| PA3944 | -1.1832 | conserved hypothetical protein | |
| *ligT* | -1.18433 | 2'-5' RNA ligase | |
| PA4396 | -1.18958 | two-component response regulator | |
| PA2447 | -1.19082 | probable transcriptional regulator | |
| PA3825 | -1.19261 | Cyclic-guanylate-specific phosphodiesterase | |
| *xcpP* | -1.19515 | secretion protein XcpP | |
| PA3368 | -1.19558 | probable acetyltransferase | |
| PA4803 | -1.19708 | hypothetical protein | |
| PA4145 | -1.20051 | probable transcriptional regulator | |
| PA2415 | -1.20255 | hypothetical protein | |
| PA0628 | -1.20461 | conserved hypothetical protein | |
| PA2711 | -1.20566 | probable periplasmic spermidine/putrescine-binding protein | |
| *czcS* | -1.20642 | CzcS |  |
| PA0443 | -1.20824 | probable transporter | |
| *foxI* | -1.21171 | ECF sigma factor FoxI | |
| PA2026 | -1.21181 | conserved hypothetical protein | |
| *nirL* | -1.21485 | heme d1 biosynthesis protein NirL | |
| *pcnA* | -1.21527 | nicotinamidase, PcnA | |
| PA1851 | -1.21817 | hypothetical protein | |
| PA5246 | -1.21878 | conserved hypothetical protein | |
| PA0951 | -1.221 | probable ribonuclease | |
| PA3897 | -1.22307 | hypothetical protein | |
| *qscR* | -1.2236 | quorum-sensing control repressor | |
| PA0167 | -1.22572 | probable transcriptional regulator | |
| PA1202 | -1.22809 | probable hydrolase | |
| *hpd* | *-1.2306* | *4-hydroxyphenylpyruvate dioxygenase* | |
| PA4383 | -1.23584 | conserved hypothetical protein | |
| PA5445 | -1.24642 | probable coenzyme A transferase | |
| PA0565 | -1.25249 | conserved hypothetical protein | |
| *cupB6* | -1.25609 | fimbrial subunit CupB6 | |
| PA2045 | -1.2568 | conserved hypothetical protein | |
| *lysP* | -1.25711 | lysine-specific permease | |
| PA2430 | -1.25715 | conserved hypothetical protein | |
| PA4167 | -1.25796 | probable oxidoreductase | |
| PA5381 | -1.26164 | hypothetical protein | |
| PA1215 | -1.26204 | hypothetical protein | |
| PA1146 | -1.26484 | probable iron-containing alcohol dehydrogenase | |
| PA4635 | -1.26603 | conserved hypothetical protein | |
| PA2137 | -1.26641 | hypothetical protein | |
| PA0021 | -1.27173 | conserved hypothetical protein | |
| PA0233 | -1.27582 | probable transcriptional regulator | |
| PA1322 | -1.27713 | probable TonB-dependent receptor | |
| *tse6* | -1.28934 | Tse6 |  |
| PA1618 | -1.2916 | conserved hypothetical protein | |
| PA4604 | -1.29612 | conserved hypothetical protein | |
| *wbpX* | -1.29692 | glycosyltransferase WbpX | |
| PA1516 | -1.29732 | hypothetical protein | |
| *dctQ* | -1.29795 | DctQ |  |
| PA1401 | -1.29998 | hypothetical protein | |
| PA2699 | -1.30236 | hypothetical protein | |
| *acsA* | -1.30827 | acetyl-coenzyme A synthetase | |
| PA1407 | -1.31109 | hypothetical protein | |
| PA1186 | -1.3111 | hypothetical protein | |
| PA0115 | -1.31904 | conserved hypothetical protein | |
| *bexR* | -1.32897 | bistable expression regulator, BexR | |
| *aruI* | -1.32928 | 2-ketoarginine decarboxylase, AruI | |
| *motD* | -1.33857 | MotD |  |
| PA2062 | -1.34562 | probable pyridoxal-phosphate dependent enzyme | |
| *wbpW* | -1.34629 | phosphomannose isomerase/GDP-mannose WbpW | |
| *czcC* | -1.34632 | outer membrane protein precursor CzcC | |
| PA1182 | -1.34679 | probable transcriptional regulator | |
| *atoB* | -1.34698 | acetyl-CoA acetyltransferase | |
| PA1235 | -1.3495 | probable transcriptional regulator | |
| *lapA* | -1.3505 | low-molecular-weight alkaline phosphatase A, LapA | |
| PA1791 | -1.35085 | hypothetical protein | |
| *soxB* | -1.35105 | sarcosine oxidase beta subunit | |
| PA4979 | -1.35841 | probable acyl-CoA dehydrogenase | |
| PA4610 | -1.36297 | hypothetical protein | |
| *bkdR* | -1.36382 | transcriptional regulator BkdR | |
| PA4039 | -1.36446 | hypothetical protein | |
| PA2431 | -1.36549 | hypothetical protein | |
| PA2063 | -1.36796 | hypothetical protein | |
| *cupA1* | -1.36976 | fimbrial subunit CupA1 | |
| *copR* | -1.37166 | two-component response regulator, CopR | |
| PA2554 | -1.37199 | probable short-chain dehydrogenase | |
| *cupA4* | -1.37429 | fimbrial subunit CupA4 | |
| PA4120 | -1.38178 | probable transcriptional regulator | |
| PA3304 | -1.38273 | conserved hypothetical protein | |
| *rocR* | -1.38543 | RocR |  |
| *glcF* | -1.38902 | glycolate oxidase subunit GlcF | |
| PA2056 | -1.39102 | probable transcriptional regulator | |
| PA2172 | -1.39148 | hypothetical protein | |
| *pelF* | -1.39233 | PelF |  |
| *yfiR* | -1.39573 | YfiR |  |
| PA2375 | -1.40389 | hypothetical protein | |
| PA4074 | -1.40466 | probable transcriptional regulator | |
| *choE* | -1.41316 | cholinesterase, ChoE | |
| *exoT* | -1.4136 | exoenzyme T | |
| *cdrB* | -1.41867 | cyclic diguanylate-regulated TPS partner B, CdrB | |
| *hsiG3* | -1.42308 | HsiG3 |  |
| PA0269 | -1.42329 | conserved hypothetical protein | |
| PA1897 | -1.42729 | hypothetical protein | |
| *aguR* | -1.4297 | transcriptional regulator AguR | |
| PA0237 | -1.4355 | probable oxidoreductase | |
| *triB* | -1.43731 | Resistance-Nodulation-Cell Division (RND) triclosan efflux membrane fusion protein, TriB | |
| *rmd* | -1.44004 | oxidoreductase Rmd | |
| *mmsB* | -1.44772 | 3-hydroxyisobutyrate dehydrogenase | |
| PA5284 | -1.44878 | hypothetical protein | |
| PA4223 | -1.44956 | probable ATP-binding component of ABC transporter | |
| PA1217 | -1.45245 | probable 2-isopropylmalate synthase | |
| PA1154 | -1.45299 | conserved hypothetical protein | |
| PA0007 | -1.45706 | hypothetical protein | |
| *pfeA* | -1.45786 | Ferric enterobactin receptor, outer membrane protein PfeA precursor | |
| PA0493 | -1.45816 | probable biotin-requiring enzyme | |
| PA3248 | -1.45938 | Uncharacterized protein | |
| PA5290 | -1.46165 | conserved hypothetical protein | |
| PA0808 | -1.46506 | hypothetical protein | |
| PA2625 | -1.46639 | conserved hypothetical protein | |
| PA0029 | -1.4715 | probable sulfate transporter | |
| *rhlA* | -1.47352 | rhamnosyltransferase chain A | |
| PA1295 | -1.4775 | conserved hypothetical protein | |
| PA3634 | -1.47808 | conserved hypothetical protein | |
| PA1876 | -1.47843 | probable ATP-binding/permease fusion ABC transporter | |
| PA1371 | -1.4842 | hypothetical protein | |
| *fliJ* | -1.48818 | flagellar protein FliJ | |
| *alg44* | -1.48837 | alginate biosynthesis protein Alg44 | |
| *siaD* | -1.48965 | SiaD |  |
| *magD* | -1.49034 | MagD |  |
| PA2076 | -1.4933 | probable transcriptional regulator | |
| PA3868 | -1.49584 | hypothetical protein | |
| PA1620 | -1.498 | hypothetical protein | |
| PA3898 | -1.4996 | probable transcriptional regulator | |
| PA4509 | -1.50274 | hypothetical protein | |
| *ctpL* | -1.50551 | CtpL |  |
| *xcpU* | -1.50879 | General secretion pathway outer membrane protein H precursor | |
| *cynS* | -1.51011 | cyanate lyase | |
| *radC* | -1.51779 | DNA repair protein RadC | |
| PA0747 | -1.51874 | probable aldehyde dehydrogenase | |
| PA1301 | -1.52048 | probable transmembrane sensor | |
| PA2752 | -1.52179 | conserved hypothetical protein | |
| PA3504 | -1.5254 | probable aldehyde dehydrogenase | |
| PA2301 | -1.53541 | hypothetical protein | |
| PA2263 | -1.53687 | probable 2-hydroxyacid dehydrogenase | |
| PA5466 | -1.54164 | hypothetical protein | |
| PA4142 | -1.54189 | probable secretion protein | |
| PA0515 | -1.54785 | probable transcriptional regulator | |
| PA1856 | -1.55129 | probable cytochrome oxidase subunit | |
| *mexG* | -1.55457 | hypothetical protein | |
| *cynT* | -1.55637 | carbonate dehydratase | |
| PA5281 | -1.55703 | probable hydrolase | |
| *eftM* | -1.56418 | SAM-dependent methyltransferase , EftM | |
| *opmQ* | -1.5718 | probable outer membrane protein precursor | |
| PA5530 | -1.57936 | C5-dicarboxylate transporter | |
| *hisM* | -1.57952 | histidine transport system permease HisM | |
| PA2441 | -1.58165 | hypothetical protein | |
| PA1473 | -1.58377 | hypothetical protein | |
| *potB* | -1.58834 | polyamine transport protein PotB | |
| PA1153 | -1.59402 | hypothetical protein | |
| PA0749 | -1.59672 | hypothetical protein | |
| PA4886 | -1.59768 | probable two-component sensor | |
| PA0756 | -1.59789 | probable two-component response regulator | |
| PA3229 | -1.60257 | hypothetical protein | |
| *aauR* | -1.60422 | AauR |  |
| PA3883 | -1.60511 | probable short-chain dehydrogenase | |
| PA5329 | -1.60525 | conserved hypothetical protein | |
| *lecA* | -1.61192 | LecA |  |
| PA1428 | -1.61893 | conserved hypothetical protein | |
| PA3232 | -1.62825 | probable nuclease | |
| PA2083 | -1.63328 | probable ring-hydroxylating dioxygenase subunit | |
| *lhpO* | -1.63366 | ABC transporter ATP-binding protein, LhpO | |
| *pvdH* | -1.63666 | L-2,4-diaminobutyrate:2-ketoglutarate 4-aminotransferase, PvdH | |
| PA5405 | -1.63691 | hypothetical protein | |
| PA2949 | -1.63705 | esterase |  |
| PA3600 | -1.65043 | conserved hypothetical protein | |
| PA0098 | -1.65787 | hypothetical protein | |
| PA1506 | -1.659 | hypothetical protein | |
| PA3180 | -1.65989 | hypothetical protein | |
| PA0226 | -1.6677 | probable CoA transferase, subunit A | |
| PA1507 | -1.67241 | probable transporter | |
| *tagF1* | -1.67433 | TagF1 |  |
| PA4155 | -1.68233 | hypothetical protein | |
| PA3750 | -1.69615 | hypothetical protein | |
| PA0564 | -1.69762 | probable transcriptional regulator | |
| PA2984 | -1.70263 | hypothetical protein | |
| *moaA1* | -1.70471 | molybdopterin biosynthetic protein A1 | |
| *napD* | -1.70692 | NapD protein of periplasmic nitrate reductase | |
| PA0986 | -1.70764 | conserved hypothetical protein | |
| *acoB* | -1.70999 | acetoin catabolism protein AcoB | |
| PA1067 | -1.71015 | probable transcriptional regulator | |
| PA5469 | -1.71086 | conserved hypothetical protein | |
| PA0146 | -1.71399 | conserved hypothetical protein | |
| *mifS* | -1.71579 | MifS |  |
| PA0952 | -1.71777 | hypothetical protein | |
| PA3589 | -1.71947 | probable acyl-CoA thiolase | |
| PA3508 | -1.72405 | probable transcriptional regulator | |
| PA4802 | -1.7288 | hypothetical protein | |
| PA4799 | -1.73446 | hypothetical protein | |
| *flgB* | -1.73567 | flagellar basal-body rod protein FlgB | |
| PA3215 | -1.73691 | probable transcriptional regulator | |
| PA0224 | -1.73778 | probable aldolase | |
| PA3590 | -1.73837 | probable hydroxyacyl-CoA dehydrogenase | |
| PA0014 | -1.75019 | hypothetical protein | |
| *pchD* | -1.75305 | pyochelin biosynthesis protein PchD | |
| PA3606 | -1.7574 | conserved hypothetical protein | |
| PA0639 | -1.76672 | conserved hypothetical protein | |
| PA3884 | -1.77258 | hypothetical protein | |
| *fpvF* | -1.77859 | FpvF |  |
| PA1419 | -1.78112 | probable transporter | |
| PA0987 | -1.78796 | conserved hypothetical protein | |
| PA3051 | -1.78818 | hypothetical protein | |
| *hsiJ3* | -1.79102 | HsiJ3 |  |
| *cbiD* | -1.7963 | cobalamin biosynthetic protein CbiD | |
| PA2596 | -1.79642 | conserved hypothetical protein | |
| *dhcA* | -1.79889 |  |  |
| *fha2* | -1.80598 | Fha2 |  |
| PA0321 | -1.80674 | acetylpolyamine amidohydrolase | |
| PA0930 | -1.81229 | two-component sensor | |
| PA0138 | -1.82049 | probable permease of ABC transporter | |
| PA0189 | -1.82099 | probable porin | |
| PA3838 | -1.83827 | probable ATP-binding component of ABC transporter | |
| PA0117 | -1.84406 | probable short chain dehydrogenase | |
| PA0914 | -1.84671 | hypothetical protein | |
| *exbB2* | -1.85618 | transport protein ExbB2 | |
| PA1924 | -1.87608 | hypothetical protein | |
| PA0364 | -1.87772 | probable oxidoreductase | |
| PA4088 | -1.87827 | probable aminotransferase | |
| PA1848 | -1.88683 | probable major facilitator superfamily (MFS) transporter | |
| *tli1* | -1.8885 | Tli1 |  |
| *flgD* | -1.89009 | flagellar basal-body rod modification protein FlgD | |
| PA3597 | -1.90194 | probable amino acid permease | |
| PA5481 | -1.91871 | hypothetical protein | |
| PA5273 | -1.91995 | hypothetical protein | |
| PA2548 | -1.921 | hypothetical protein | |
| *ansA* | -1.92314 | L-asparaginase I | |
| *dhcB* | -1.93478 |  |  |
| PA0137 | -1.94274 | probable permease of ABC transporter | |
| PA0532 | -1.94747 | hypothetical protein | |
| PA2704 | -1.95339 | probable transcriptional regulator | |
| *narX* | -1.96405 | two-component sensor NarX | |
| PA2680 | -1.96994 | probable quinone oxidoreductase | |
| PA4594 | -1.98409 | probable ATP-binding component of ABC transporter | |
| *hcp1* | -1.98445 | Hcp1 |  |
| PA2135 | -2.00204 | probable transporter | |
| PA4906 | -2.00523 | probable transcriptional regulator | |
| PA5116 | -2.00565 | probable transcriptional regulator | |
| PA4008 | -2.01126 | probable hydrolase | |
| *aphA* | -2.01289 | acetylpolyamine aminohydrolase | |
| PA3445 | -2.02344 | conserved hypothetical protein | |
| *exbB1* | -2.02887 | transport protein ExbB | |
| *shaB* | -2.03628 | ShaB |  |
| PA5185 | -2.0378 | conserved hypothetical protein | |
| PA3671 | -2.04705 | probable permease of ABC transporter | |
| PA0915 | -2.0474 | conserved hypothetical protein | |
| *cobM* | -2.05123 | precorrin-3 methylase | |
| PA4341 | -2.05658 | probable transcriptional regulator | |
| PA4136 | -2.07409 | probable major facilitator superfamily (MFS) transporter | |
| PA2175 | -2.08376 | hypothetical protein | |
| PA1286 | -2.08877 | probable major facilitator superfamily (MFS) transporter | |
| PA4192 | -2.10751 | probable ATP-binding component of ABC transporter | |
| PA1353 | -2.12021 | hypothetical protein | |
| *cupB2* | -2.12205 | chaperone CupB2 | |
| PA2747 | -2.1392 | hypothetical protein | |
| PA1362 | -2.15973 | hypothetical protein | |
| PA4149 | -2.17173 | conserved hypothetical protein | |
| PA5033 | -2.17565 | hypothetical protein | |
| *hxcS* | -2.2054 | HxcS |  |
| PA2487 | -2.23073 | hypothetical protein | |
| *phnC* | -2.24104 | ATP-binding component of ABC phosphonate transporter | |
| PA0734 | -2.25486 | hypothetical protein | |
| PA0256 | -2.25844 | hypothetical protein | |
| PA1485 | -2.26709 | probable amino acid permease | |
| *fliR* | -2.27685 | flagellar biosynthetic protein FliR | |
| PA2070 | -2.30276 | hypothetical protein | |
| *cysA* | -2.30986 | sulfate transport protein CysA | |
| PA1548 | -2.33118 | conserved hypothetical protein | |
| *agtA* | -2.38396 | AgtA |  |
| PA2282 | -2.39043 | hypothetical protein | |
| *pelB* | -2.39181 | PelB |  |
| PA4573 | -2.40197 | hypothetical protein | |
| PA3847 | -2.40924 | conserved hypothetical protein | |
| PA0874 | -2.41403 | hypothetical protein | |
| *agtD* | -2.41808 | AgtD |  |
| PA3596 | -2.43267 | probable methylated-DNA--protein-cysteine methyltransferase | |
| PA0563 | -2.44081 | conserved hypothetical protein | |
| *antR* | -2.45236 | AntR |  |
| PA3882 | -2.45373 | hypothetical protein | |
| PA4637a | -2.45425 |  |  |
| PA1138 | -2.48819 | probable transcriptional regulator | |
| PA0793 | -2.51633 | hypothetical protein | |
| *vgrG4b* | -2.5315 | VgrG4b |  |
| *ambD* | -2.53963 | AmbD |  |
| PA4978 | -2.55795 | hypothetical protein | |
| PA5352 | -2.55883 | conserved hypothetical protein | |
| *sppD* | -2.55917 | ABC transporter ATP-binding protein, SppD | |
| PA4926 | -2.5852 | conserved hypothetical protein | |
| PA5102 | -2.61315 | hypothetical protein | |
| PA1619 | -2.61885 | probable transcriptional regulator | |
| *lsfA* | -2.6426 | 1-Cys peroxiredoxin LsfA | |
| *grx* | -2.65371 |  |  |
| PA1185 | -2.66948 | probable glutathione S-transferase | |
| PA2490 | -2.67917 | conserved hypothetical protein | |
| PA1302 | -2.71107 | probable heme utilization protein precursor | |
| PA0440 | -2.74206 | probable oxidoreductase | |
| *tadC* | -2.78116 | TadC |  |
| *tssF1* | -2.81004 | TssF1 |  |
| PA0234 | -2.8361 | hypothetical protein | |
| *pvdQ* | -2.87263 | 3-oxo-C12-homoserine lactone acylase PvdQ | |
| *tsi4* | -2.88451 | Tsi4 |  |
| *qteE* | -2.9029 | quorum threshold expression element, QteE | |
| *hutR* | -2.91801 | HutR |  |
| PA5191 | -2.93795 | hypothetical protein | |
| PA3033 | -3.08932 | hypothetical protein | |
| PA3881 | -3.15272 | hypothetical protein | |
| *rocS1* | -3.24903 | two-component sensor RocS1 | |
| PA5444 | -3.37061 | conserved hypothetical protein | |
| *narL* | -3.41525 | two-component response regulator NarL | |
