# Supplementary material for: Pseudomonas aeruginosa Regulatory Protein AnvM Controls Pathogenicity in Anaerobic Environments and Impacts Host Defense
Source: mBio. 2019 Jul 23;10(4):e01362-19. doi: 10.1128/mBio.01362-19 (PMC6650552; doi:10.1128/mBio.01362-19)
Supplement: TABLE S2 [file mBio.01362-19-st002.docx]

**Table S2A. LC-MS analysis of compounds bound to AnvM.**

| **PA number** | **Gene name** | **Score** | **Description** | |  |  |
| --- | --- | --- | --- | --- | --- | --- |
| PA3165 | *hisC2* | 5.189 | Histidinol-phosphate aminotransferase | | | |
| PA3538 |  | 4.735 | Probable ATP-binding component of ABC transporter | | | |
| PA4842 |  | 4.08 | Uncharacterized protein | | |  |
| PA3169 |  | 4.008 | Methylthioribose-1-phosphate isomerase | | | |
| PA0421 |  | 3.62 | Uncharacterized protein | | |  |
| PA5076 |  | 3.604 | Probable binding protein component of ABC transporter | | | |
| PA4958 |  | 3.453 | Uncharacterized protein | | |  |
| PA3155 | *wbpE* | 3.444 | UDP-2-acetamido-2-deoxy-3-oxo-D-glucuronate aminotransferase | | | |
| PA0280 | *cysA* | 3.305 | Sulfate/thiosulfate import ATP-binding protein CysA | | | |
| PA2330 |  | 3.068 | Uncharacterized protein | | |  |
| PA0657 |  | 2.961 | Probable ATPase | |  |  |
| PA4322 |  | 2.895 | Uncharacterized protein | | |  |
| PA4224 | *pchG* | 2.851 | Pyochelin biosynthetic protein PchG | | | |
| PA0609 | *trpE* | 2.844 | Anthranilate synthase component 1 | | | |
| PA1003 | *mvfR* | 2.781 | Transcriptional regulator MvfR | | |  |
| PA0888 | *aotJ* | 2.337 | Arginine/ornithine binding protein AotJ | | | |
| PA2841 |  | 2.306 | Probable enoyl-CoA hydratase/isomerase | | | |
| PA0592 | *ksgA* | 2.227 | Ribosomal RNA small subunit methyltransferase A | | | |
| PA3070 |  | 2.015 | Uncharacterized protein | | |  |
| PA0936 | *lpxO2* | 1.968 | Lipopolysaccharide biosynthetic protein LpxO2 | | | |
| PA5332 | *crc* | 1.881 | Catabolite repression control protein | | | |
| PA3158 | *wbpB* | 1.77 | UDP-N-acetyl-2-amino-2-deoxy-D-glucuronate oxidase | | | |
| PA0662 | *argC* | 1.757 | N-acetyl-gamma-glutamyl-phosphate reductase | | | |
| PA3084 |  | 1.398 | Uncharacterized protein | | |  |
| PA1766 |  | 1.397 | Uncharacterized protein | | |  |
| PA1551 |  | 1.176 | Probable ferredoxin | |  |  |
| PA1832 |  | 1.15 | Probable protease | |  |  |
| PA4372 |  | 1.143 | Uncharacterized protein | | |  |
| PA4292 |  | 1.094 | Phosphate transporter | | |  |
| PA0902 |  | 1.093 | Uncharacterized protein | | |  |
| PA4356 | *xenB* | 1.034 | Xenobiotic reductase | | |  |
| PA3724 | *lasB* | 1.028 | Elastase |  |  |  |
| PA0314 |  | 0.945 | L-cysteine transporter of ABC system FliY | | | |
| PA3818 |  | 0.912 | Inositol-1-monophosphatase | | |  |
| PA1520 |  | 0.844 | Probable transcriptional regulator | | | |
| PA2740 | *pheS* | 0.771 | Phenylalanine--tRNA ligase alpha subunit | | | |
| PA5125 | *ntrC* | 0.763 | DNA-binding transcriptional regulator NtrC | | | |
| PA4787 |  | 0.757 | Probable transcriptional regulator | | | |
| PA2643 | *nuoH* | 0.753 | NADH-quinone oxidoreductase subunit H | | | |
| PA1642 | *selD* | 0.728 | Selenide, water dikinase | | |  |
| PA4548 |  | 0.668 | Glycine oxidase | |  |  |
| PA3799 |  | 0.646 | GTPase |  |  |  |
| PA1544 | *anr* | 0.584 | Transcriptional activator protein anr | | | |

**Table S2B. The expression of MvfR-associated genes in PAO1 and Δ*anvM* under the aerobic condition.**

| **PA number** | **Gene name** | **Fold change aerobic ΔPA3880 vs. aerobic WT (log2)** | **Description** | |  |
| --- | --- | --- | --- | --- | --- |
| PA2193 | *hcnA* | 2.86391 | hydrogen cyanide synthase HcnA+ | | |
| PA1556 | *ccoO2* | 1.67226 | Cytochrome c oxidase, cbb3-type, CcoO subunit+ | | |
| PA1914 |  | 1.60225 | conserved hypothetical protein+ | | |
| PA1910 | *femA* | 1.53616 | ferric-mycobactin receptor, FemA- | | |
| PA3098 | *xcpW* | 1.45135 | general secretion pathway protein + | | |
| PA1659 | *hsiF2* | 1.40742 | HsiF2+ |  |  |
| PA2299 |  | 1.36974 | probable transcriptional regulator+ | | |
| PA2067 |  | 1.20927 | probable hydrolase+ | |  |
| PA1130 | *nalC* | 1.20601 | NalC+ |  |  |
| PA4205 | *mexG* | 1.14369 | hypothetical protein+ | | |
| PA0567 |  | 1.06907 | conserved hypothetical protein+ | | |
| PA2172 |  | 1.04351 | hypothetical protein+ | | |
| PA5481 |  | 1.04263 | hypothetical protein+ | | |
| PA0849 | *trxB2* | 1.02608 | thioredoxin reductase 2+ | | |
| PA3403 |  | 0.917795 | hypothetical protein- | |  |
| PA3812 | *iscA* | -0.96685 | probable iron-binding protein IscA+ | | |
| PA1001 | *phnA* | -0.98483 | anthranilate synthase component I- | | |
| PA2204 |  | -1.00115 | probable binding protein component of ABC transporter- | | |
| PA0997 | *pqsB* | -1.09054 | PqsB- |  |  |
| PA4496 | *dppA1* | -1.16682 | probable binding protein component of ABC transporter- | | |
| PA4225 | *pchF* | -1.26759 | pyochelin synthetase- | | |
| PA0996 | *pqsA* | -1.30026 | PqsA- |  |  |
| PA3479 | *rhlA* | -1.32788 | rhamnosyltransferase chain A- | | |
| PA4940 |  | -1.39438 | conserved hypothetical protein- | | |
| PA3813 | *iscU* | -1.40211 | probable iron-binding protein IscU- | | |
| PA1130 | *rhlC* | -1.47335 | rhamnosyltransferase 2- | | |
| PA2662 |  | -1.54131 | conserved hypothetical protein+ | | |
| PA0200 |  | -1.58061 | hypothetical protein- | |  |
| PA4226 | *pchE* | -1.71633 | dihydroaeruginoic acid synthetase- | | |
| PA0048 |  | -1.75818 | probable transcriptional regulator+ | | |
| PA1912 | *femI* | -1.77965 | ECF sigma factor, FemI- | | |
| PA2274 |  | -1.79971 | hypothetical protein- | |  |
| PA2031 |  | -2.26613 | hypothetical protein- | |  |
| PA4224 | *pchG* | -2.3411 | pyochelin biosynthetic protein PchG- | | |
| PA2663 | *ppyR* | -2.3765 | psl and pyoverdine operon regulator+ | | |
| PA1000 | *pqsE* | -2.55153 | Quinolone signal response protein- | | |
| PA1911 | *femR* | -3.19023 | sigma factor regulator, FemR- | | |

**Table S2C.The expression of MvfR-associated genes in PAO1 and Δ*anvM* under the anaerobic condition.**

| **PA number** | **Gene name** | **Fold change anaerobic ΔPA3880 vs. anaerobic WT (log2)** | **Description** | |
| --- | --- | --- | --- | --- |
| PA0589 |  | 3.05435 | conserved hypothetical protein | |
| PA1275 | *cobD* | 2.27282 | cobalamin biosynthetic protein CobD | |
| PA1277 | *cobQ* | 2.26473 | cobyric acid synthase | |
| PA3403 |  | 2.16952 | hypothetical protein | |
| PA3476 | *rhlI* | 1.76341 | autoinducer synthesis protein RhlI | |
| PA4190 | *pqsL* | 1.71827 | probable FAD-dependent monooxygenase | |
| PA1130 | *rhlC* | 1.25222 | rhamnosyltransferase 2 | |
| PA2195 | *hcnC* | 1.16629 | hydrogen cyanide synthase HcnC | |
| PA0996 | *pqsA* | 1.15592 | PqsA |  |
| PA0849 | *trxB2* | 0.925445 | thioredoxin reductase 2 | |
| PA2274 |  | 0.911332 | hypothetical protein | |
| PA3102 | *xcpS* | -0.90816 | general secretion pathway protein F | |
| PA0283 | *sbp* | -0.95197 | sulfate-binding protein precursor | |
| PA1432 | *lasI* | -0.97965 | autoinducer synthesis protein LasI | |
| PA2204 |  | -0.98696 | probable binding protein component of ABC transporter | |
| PA0998 | *pqsC* | -1.03515 | PqsC |  |
| PA1272 | *cobO* | -1.12897 | cob(I)alamin adenosyltransferase | |
| PA2329 |  | -1.16163 | probable ATP-binding component of ABC transporter | |
| PA3104 | *xcpP* | -1.19515 | secretion protein XcpP | |
| PA2128 | *cupA1* | -1.36976 | fimbrial subunit CupA1 | |
| PA2131 | *cupA4* | -1.37429 | fimbrial subunit CupA4 | |
| PA2172 |  | -1.39148 | hypothetical protein | |
| PA3569 | *mmsB* | -1.44772 | 3-hydroxyisobutyrate dehydrogenase | |
| PA3479 | *rhlA* | -1.47352 | rhamnosyltransferase chain A | |
| PA3100 | *xcpU* | -1.50879 | General secretion pathway outer membrane protein H precursor | |
| PA4205 | *mexG* | -1.55457 | hypothetical protein | |
| PA5481 |  | -1.91871 | hypothetical protein | |
| PA0085 | *hcp1* | -1.98445 | Hcp1 |  |
| PA2747 |  | -2.1392 | hypothetical protein | |

**Table S2D. The expression of Anr-associated genes in PAO1 and PA3880 mutant under the anaerobic condition.**

| **PA number** | **Gene name** | **Fold change anaerobic ΔPA3880 vs. anaerobic WT**  **(log2)** | | **Description** | |  |
| --- | --- | --- | --- | --- | --- | --- |
| PA3394 | *nosF* | 2.29433 | NosF protein- | | |  |
| PA0613 |  | 1.65132 | hypothetical protein+ | | | |
| PA1317 | *cyoA* | 1.63469 | cytochrome o ubiquinol oxidase subunit II+ | | | |
| PA0630 |  | 1.40433 | hypothetical protein+ | | | |
| PA2165 |  | 1.39024 | probable glycogen synthase- | | | |
| PA3874 | *narH* | 1.29565 | respiratory nitrate reductase beta chain- | | | |
| PA1123 |  | 1.27697 | hypothetical protein- | | |  |
| PA0866 | *aroP2* | 1.12733 | aromatic amino acid transport protein AroP2+ | | | |
| PA1978 | *erbR* | 1.07976 | response regulator ErbR+ | | | |
| PA3233 |  | 1.05286 | hypothetical protein+ | | | |
| PA0509 | *nirN* | -1.00007 | NirN+ | |  |  |
| PA3268 |  | -1.05172 | probable TonB-dependent receptor- | | | |
| PA4620 |  | -1.07822 | hypothetical protein- | | |  |
| PA1052a |  | -1.0866 | + | |  |  |
| PA2839 |  | -1.10038 | conserved hypothetical protein+ | | | |
| PA1870 |  | -1.16838 | hypothetical protein+ | | | |
| PA0628 |  | -1.20461 | conserved hypothetical protein- | | | |
| PA0514 | *nirL* | -1.21485 | heme d1 biosynthesis protein NirL+ | | | |
| PA5445 |  | -1.24642 | probable coenzyme A transferase- | | | |
| PA0887 | *acsA* | -1.30827 | acetyl-coenzyme A synthetase- | | | |
| PA5416 | *soxB* | -1.35105 | sarcosine oxidase beta subunit- | | | |
| PA4610 |  | -1.36297 | hypothetical protein+ | | | |
| PA2131 | *cupA4* | -1.37429 | fimbrial subunit CupA4+ | | | |
| PA0515 |  | -1.54785 | probable transcriptional regulator+ | | | |
| PA5530 |  | -1.57936 | C5-dicarboxylate transporter- | | | |
| PA0226 |  | -1.6677 | probable CoA transferase, subunit A- | | | |
| PA4228 | *pchD* | -1.75305 | pyochelin biosynthesis protein PchD+ | | | |
| PA0639 |  | -1.76672 | conserved hypothetical protein- | | | |
| PA1665 | *fha2* | -1.80598 | Fha2+ | |  |  |
| PA3878 | *narX* | -1.96405 | two-component sensor NarX+ | | | |
| PA0198 | *exbB1* | -2.02887 | transport protein ExbB+ | | | |
| PA0280 | *cysA* | -2.30986 | sulfate transport protein CysA- | | | |
| PA2511 | *antR* | -2.45236 | AntR- | |  |  |
| PA3879 | *narL* | -3.41525 | two-component response regulator NarL+ | | | |
